# Fine mapping of heterozygous *IL6ST* nonsense variants underlying autosomal dominant hyper-IgE syndrome

**DOI:** 10.1172/jci.insight.190065

**Published:** 2025-06-17

**Authors:** Kosuke Ashihara, Takaki Asano, Kanako Takeuchi, Kosuke Noma, Miyuki Tsumura, Wenjie Wang, Wei-Te Lei, Hisao Higo, Toshio Kubo, Yoko Mizoguchi, Shuhei Karakawa, Aurélie Cobat, Clément Conil, Etsushi Toyofuku, Akimasa Sekine, Kohsuke Imai, Dusan Bogunovic, Jean-Laurent Casanova, Cheng-Lung Ku, Vivien Béziat, Satoshi Okada

**Affiliations:** 1 Department of Pediatrics, Hiroshima University Graduate School of Biomedical and Health Sciences, Hiroshima, Japan.; 2 Department of Radiation Biophysics, Research Institute for Radiation Biology and Medicine, Hiroshima University, Hiroshima, Japan.; 3 Department of Clinical Immunology, Children’s Hospital of Fudan University, National Children’s Medical Center, Shanghai, China.; 4 Laboratory of Human Immunology and Infectious Diseases, Graduate Institute of Clinical Medical Sciences, Chang Gung University, Taoyuan, Taiwan.; 5 Division of Immunology, Rheumatology, and Allergy, Department of Pediatrics, Hsinchu Municipal MacKay Children’s Hospital, Hsinchu, Taiwan.; 6 Department of Allergy and Respiratory Medicine, Okayama University Hospital, Okayama, Japan.; 7 Laboratory of Human Genetics of Infectious Diseases, Necker Branch, INSERM, Necker Hospital for Sick Children, Paris, France.; 8 Imagine Institute, University of Paris-Cité, Paris, France.; 9 St. Giles Laboratory of Human Genetics of Infectious Diseases, Rockefeller Branch, The Rockefeller University, New York, New York, USA.; 10 Department of Pediatrics and Developmental Biology, Tokyo Medical and Dental University (TMDU), Tokyo, Japan.; 11 Department of Rheumatology and Allergology, St. Marianna University School of Medicine, Kanagawa, Japan.; 12 Department of Respiratory Medicine, Kanagawa Cardiovascular and Respiratory Center, Kanagawa, Japan.; 13 Department of Pediatrics, National Defense Medical College, Saitama, Japan.; 14 Center for Genetic Errors of Immunity, Department of Pediatrics, Columbia University, New York, New York, USA.; 15 Center for Inborn Errors of Immunity, Icahn School of Medicine at Mount Sinai, New York, New York, USA.; 16 Department of Pediatrics, Necker Hospital for Sick Children, AP-HP, Paris, France.; 17 Howard Hughes Medical Institute, The Rockefeller University, New York, New York, USA.; 18 Center for Molecular and Clinical Immunology, Chang Gung University, Taoyuan, Taiwan.; 19 Division of Infectious Diseases, Department of Pediatrics, Chang Gung Memorial Hospital, Taoyuan, Taiwan.

**Keywords:** Genetics, Immunology, Cytokines, Innate immunity, Molecular genetics

## Abstract

Loss-of-function (LOF) variants in *IL6ST*, encoding GP130, can cause hyper-IgE syndrome (HIES). Monoallelic LOF variants in *IL6ST* lead to HIES when located in the intracellular domain downstream of box 1/2 and upstream of the STAT3 phosphorylation sites and the recycling motif, due to their dominant negative (DN) activity. In this region, 2 previously unreported *IL6ST* variants, p.K702Sfs7* and p.Y759Wfs26*, were identified in 2 families with autosomal dominant (AD) HIES. Both variants were LOF and exhibited DN effects, leading to the accumulation of mutant GP130 on the cell surface. The p.K702Sfs7* mutation was the most upstream N-terminal mutation linked to HIES caused by heterozygous *IL6ST* variants. Comprehensive screening of *IL6ST* mutants revealed that most premature terminations downstream of amino acid F641, at the end of the transmembrane domain, resulted in LOF and DN effects via GP130 accumulation on the cell surface. The absence of the recycling motif (positions 782–787) in surface-expressed LOF GP130 led to its accumulation, contributing to the DN effect. The importance of intracellular truncating *IL6ST* variants can possibly be predicted based on the location of the premature stop codon. GP130 accumulation on the cell surface is a characteristic and potentially diagnostic finding in patients with HIES with heterozygous *IL6ST* variants.

## Abstract 2

The inheritance patterns of IL6ST variants may depend on the stop codon position, with GP130 accumulation serving as a diagnostic marker for autosomal inheritance.

## Graphical abstract

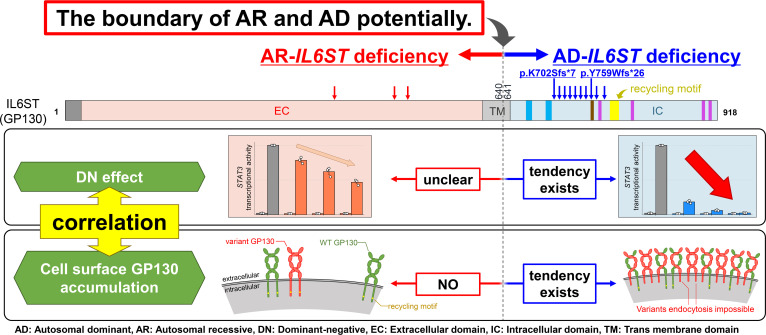

## Introduction

Hyper-IgE syndrome (HIES), initially described by Davis et al. in 1966 as Job’s syndrome, is characterized by recurrent cold staphylococcal abscesses (lacking inflammatory features), sinus and lung infections, and severe eczema, which appear during the neonatal period (1). Patients with HIES also present with other infection-related syndromes, including eosinophilia immediately before acute infection and chronic mucocutaneous candidiasis (CMC). Additionally, many patients with HIES exhibit skeletal abnormalities, such as characteristic facial features, retained primary teeth, craniosynostosis, scoliosis, and a proneness to fractures (2, 3). In 2007, heterozygous missense mutations in the signal transducer and activator of transcription 3 (*STAT3*) gene were identified as the first genetic etiology of HIES (4), with the basic molecular mechanism involving a dominant-negative (DN) effect on STAT3 (5). Since then, 10 genes (*STAT3*, *IL6R*, *ZNF341*, *ERBB2IP*, *TGFBR1/TGFBR2*, *SPINK5*, *PGM3*, *CARD11*, and *IL6ST*) have been classified as causative genes for HIES by the latest classification of the International Union of Immunological Societies (IUIS) in 2022 (6).

**Figure 1:**
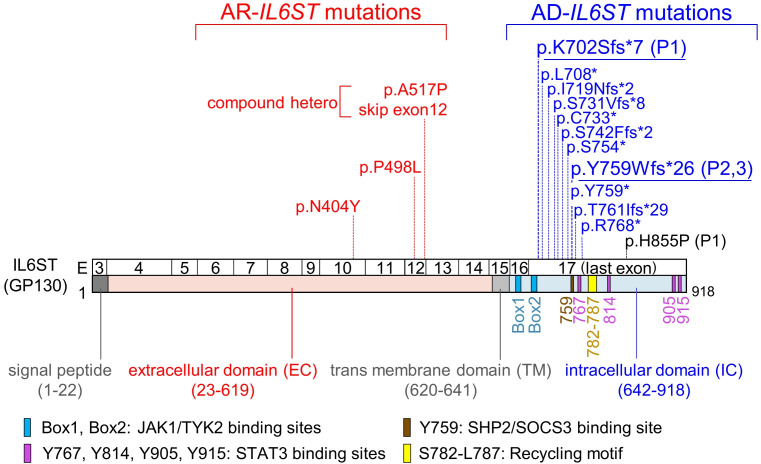
Previously reported *IL6ST* mutations. Schematic diagram of GP130 and reported mutations. The locations of all reported AR-*IL6ST* mutations and AD-*IL6ST* mutations in GP130 are shown. AR-*IL6ST* mutations are colored red, AD-*IL6ST* mutations are colored blue, and mutations we identified in this study (p.K702Sfs*7, p.Y759Wfs*26) are underlined. The gnomAD missense variant (p.H855P) found in P1 is shown in black. E, exon; EC, extracellular domain; TM, transmembrane domain; IC, intracellular domain.

The *IL6ST* gene was identified as encoding the GP130 protein, which mediates IL-6 signaling in conjunction with IL-6R (7, 8). It also mediates the effects of all other IL-6 family cytokines: IL-11, IL-27, IL-35, leukemia inhibitory factor (LIF), oncostatin M (OSM), cardiotrophin 1 (CT-1), cardiotrophin-like cytokine (CLC), and ciliary neurotrophic factor (CNTF) (9) (Supplemental Figure 1; supplemental material available online with this article; https://doi.org/10.1172/jci.insight.190065DS1). When ligands such as IL-6 bind to their receptors, the JAK1 kinase is activated and autophosphorylates itself; it subsequently phosphorylates STAT3 and, to a minor extent, STAT1, thereby initiating the signaling cascade. Patients with homozygous amorphic mutations in *IL6ST* (10) or *LIF* receptor (*LIFR*) (11) develop Stüve-Wiedemann syndrome. In addition, partial impairment of GP130 due to biallelic hypomorphic *IL6ST* mutations results in autosomal recessive (AR) HIES (12–14). On the other hand, autosomal dominant (AD) HIES due to heterozygous *IL6ST* mutations with a DN effect has been recently reported (15,16). Partial AR and AD GP130 deficiencies mostly impair IL-6 and IL-11 signaling, explaining why patients do not develop Stüve-Wiedemann syndrome (12–16). The GP130 protein is composed of a signal peptide, an extracellular domain (EC), a transmembrane domain (TM), and an intracellular domain (IC) (Figure 1). The IC contains JAK1/TYK2 binding sites (referred to as Box1 and Box2), a SHP2/SOCS3 binding site, 4 STAT3 binding sites, and a recycling motif crucial for endocytosis (17) (Figure 1). Notably, reported mutations associated with AR GP130 deficiency (AR-*IL6ST* mutations) are clustered within the EC, while mutations associated with AD GP130 deficiency (AD-*IL6ST* mutations), all of which are nonsense or frameshift mutations, are clustered in the IC (Figure 1). All mutations reported in patients with AD GP130 deficiency lack the recycling motif, leading to the accumulation of GP130 mutants on the cell surface in most cases (15, 16). This accumulation of GP130 is presumed to be responsible for the strong DN effect (15).

To the best of our knowledge, we herein report the first 3 cases of AD GP130 deficiency in East Asia, which occurred due to potentially novel DN mutations in *IL6ST*. These patients exhibited phenotypes similar to those previously reported (15, 16). The substantial advancements in comprehensive genetic analysis techniques have underscored the importance of determining the pathological relevance of identified variants. Based on the positional accumulation patterns of *IL6ST* mutations, we hypothesized that there is an amino acid (aa) that distinguishes between AR- and AD-*IL6ST* mutations. Identifying such an aa would facilitate the estimation of the functional importance of unknown variants in *IL6ST*, potentially contributing to accurate diagnosis in patients with HIES. Based on a series of experimental verifications, we identified the possible aa that determines the functional effect of unknown variants in *IL6ST*. This study also revealed that most reported AD-*IL6ST* mutations exhibit DN effects, with cell-surface GP130 accumulation. We also discuss the potential use of these findings for the rapid diagnosis of AD GP130 deficiency.

## Results

### Case reports.

We investigated 3 patients from 2 unrelated families (Table 1). P1, from family 1, was a 32-year-old Japanese man. He was born to nonconsanguineous Japanese parents and experienced delayed umbilical cord prolapse after birth. As a newborn, he had severe eczema. At age 1, he developed asthma and started inhalation therapy but was repeatedly hospitalized thereafter. When he was 26 years old, he presented with a cough and bloody sputum, and chest computed tomography (CT) revealed bronchiectasis. His serum IgE level was extremely high at 12,565 IU/mL, and he tested positive for anti–*Staphylococcus aureus* IgE antibodies. During the course of the disease, *Aspergillus* was detected in his sputum, and CT revealed mucoid impaction and eosinophilia. He was subsequently diagnosed with allergic bronchopulmonary aspergillosis (ABPA), which led to the initiation of steroid therapy. He was also treated for atopic dermatitis with mepolizumab (anti–IL-5 antibody) and benralizumab (anti–IL-5Rα antibody), but the treatments were not very effective. Currently, his symptoms have stabilized with the administration of dupilumab (anti–IL-4Rα/IL-13Rα antibody), and the use of steroids has been discontinued. In addition, he had retained 3 primary teeth and was also susceptible to bone fractures (multiple rib fractures), without characteristic facial features. The NIH HIES score for P1 was 39 (Table 1). His father, who has since died after suffering from prostate cancer, also exhibited HIES symptoms, such as atopic dermatitis and retained primary teeth. The sister of P1 had symptoms of thoracic scoliosis, palmoplantar pustulosis, and herpes labialis.

P2, in family 2, was a 33-year-old Japanese man. He was born to nonconsanguineous Japanese parents and had eczema since his childhood. High serum IgE levels were noted when he contracted cellulitis at age 8. The patient was started on prophylactic antimicrobial therapy for recurrent skin abscesses, lymphadenitis, and pneumonia without CRP elevation. He was diagnosed with ABPA and treated when he was 23 years old; after initial improvement, he experienced a relapse of ABPA when he was 26 years old and received treatment again. Otherwise, he had not been treated with biological products for atopic dermatitis. In addition, 1 retained deciduous tooth was observed, with no information on susceptibility to bone fracture or characteristic facial features. He never had abnormalities in immunoglobulins other than IgE. The NIH HIES score for P2 was 51 (Table 1). P3 (P2’s father), who was 61 years old, had atopic dermatitis since his neonatal period, high serum IgE levels, eosinophilia, 2 retained primary teeth, severe joint infections, and a history of asthma. The NIH HIES score for P3 was 38. (Table 1).

### Identification of previously unreported variants associated with AD GP130 deficiency.

**Figure 2:**
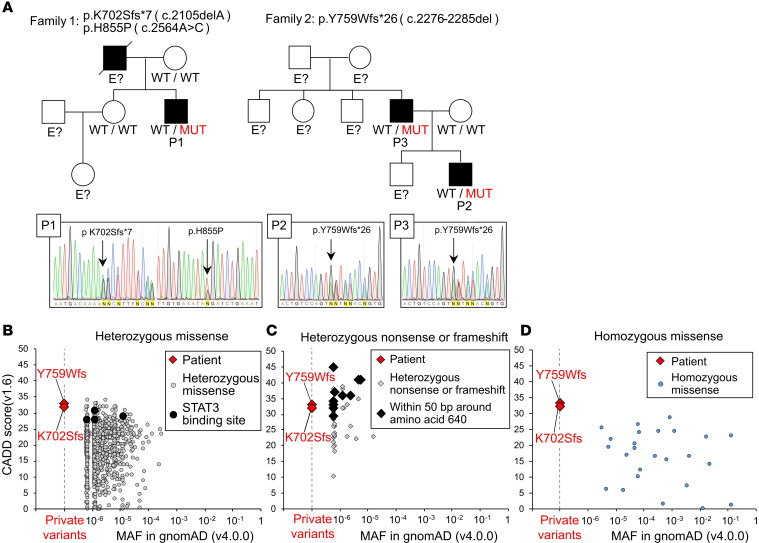
Familial cases of AD GP130 deficiency. (**A**) The mutations c.2105delA (p.K702Sfs*7) and c.2564A>C (p.H855P) were found in P1 of family 1, while the mutation c.2276-2285del (p.Y759Wfs*26) was present in P2 and P3 of family 2. Individuals of unknown genotype are labeled “E?”. (**B**–**D**) Predicted CADD scores (https://cadd.gs.washington.edu/) and global allele frequencies of *IL6ST* variants, as reported in the gnomAD database (v4.0.0). The red diamonds indicate the variants of our patient, whose CADD score was 32 or 33. The gray dots indicate heterozygous missense variants, with those at the STAT3 binding sites indicated by black dots (**B**). The gray diamonds indicate heterozygous nonsense or frameshift variants, with those around aa 640 (50 aa before and after) indicated by black diamonds (**C**). Blue dots indicate homozygous missense variants (**D**). MUT, mutant; MAF, minor allele frequency; CADD, combined annotation-dependent depletion.

Whole exome sequencing (WES) for P1 revealed 2 variants in *IL6ST*: a heterozygous single-nucleotide deletion, c.2105delA (p.K702Sfs*7, abbreviated as p.K702Sfs), and a heterozygous single-nucleotide substitution, c.2564A>C (p.H855P), in *IL6ST* (Figure 2A). The p.H855P variant has been listed in gnomAD v4.0.0 (minor allele frequency [MAF] = 4.46 × 10^–5^) and reported in 72 individuals in the heterozygous state, mostly of East Asian origin. Notably, the p.H855P variant was untranslated because it is located on the same allele, downstream of p.K702fs, according to the Sanger sequencing chromatogram (Supplemental Figure 2). The father of patient P1 had died, so Sanger sequencing was not performed. Sanger sequencing of his sister did not reveal the same *IL6ST* mutation as in him. Furthermore, she also never had any other previously reported pathogenic mutation in WES. WES for P2 revealed a heterozygous 10-bp deletion, c.2276-2285delATTCTACCGT (p.Y759Wfs*26, abbreviated as p.Y759Wfs), in *IL6ST* (Figure 2A). Sanger sequencing confirmed that the variant in P3 was the same as that in P2. Neither the p.K702Sfs nor the p.Y759Wfs variant is listed in databases such as gnomAD v4.0.0, UK Biobank and ClinVar. Importantly, the p.K702Sfs variant is located closest to the N-terminus among reported mutations associated with AD GP130 deficiency (Figure 1). On the other hand, p.Y759Wfs caused a frameshift starting from the SHP2/SOCS3 binding site. Both p.K702Sfs and p.Y759Wfs *IL6ST* variants were estimated to be pathogenic based on the combined annotation-dependent depletion (CADD) scores (18), which were 32 and 33, respectively (Figure 2, B–D). Notably, both variants were predicted to escape nonsense-mediated mRNA decay (NMD) (19) because of the premature stop codon in the last exon.

### Detailed analysis of blood cell subsets in patients with AD GP130 deficiency with previously unreported mutations.

**Figure 3:**
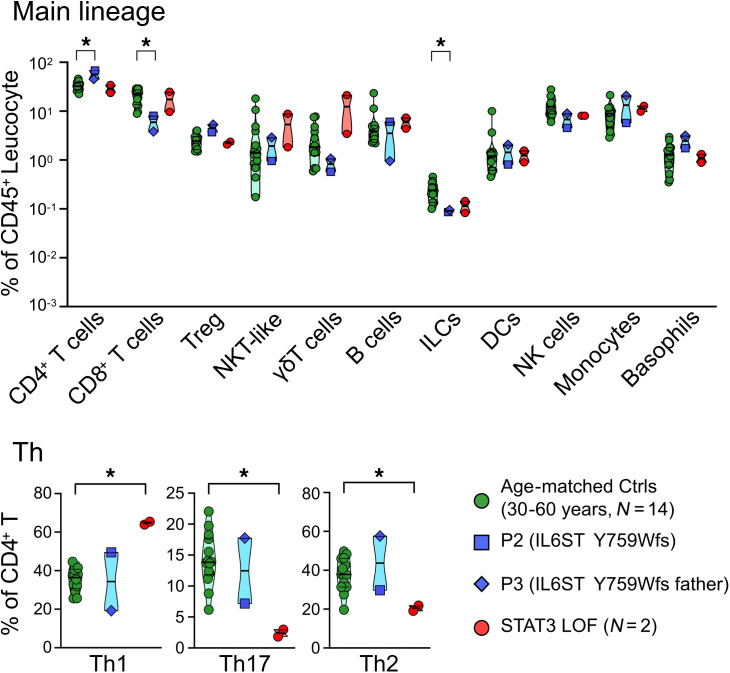
Deep immunophenotyping of patients with AD-*IL6ST* mutation Y759Wfs. This deep immunophenotyping for patients’ PBMCs was performed using Aurora (Cytek). Age-matched healthy controls (30–60 years, *n* = 14) are represented by green dots, P2 by blue squares, P3 by blue diamonds, and the patients with heterozygous DN *STAT3* mutation (p.V637M, p.Y657C) by red dots (*N* = 2). Statistical analysis was done with 2-tailed Mann-Whitney *U* test. *P < 0.05. ILC, innate lymphoid cell.

Immunophenotyping of P1 revealed a decrease in B cells, Th1 cells, and CD4^+^ and CD8^+^ memory T cells and an increase in Th2 cells (Supplemental Table 1) (20). Immunophenotyping of P2 and P3 revealed low levels of CD8^+^ T cells, innate lymphoid cells, CD4^+^ terminally differentiated effector memory cells re-expressing CD45RA (TEMRA), and circulating follicular helper T (cTfh) cells, as well as a decreasing trend in γδT cells, NK cells, CD4^+^ central memory cells, CD4^+^ effector memory cells, and class-switched memory B cells. Additionally, there was an increase in CD4^+^ T cells and CD4^+^ naive T cells (Figure 3 and Supplemental Figure 3). In our analysis, the immunological phenotypes of P2 and P3 were milder compared with patients with heterozygous DN *STAT3* mutations (p.V637M, p.Y657C), as they did not exhibit a reduction in Th17 or an increase in Th1.

### The p.K702Sfs and p.Y759Wfs variants are LOF variants in STAT3 signaling and accumulate on the cell surface.

**Figure 4:**
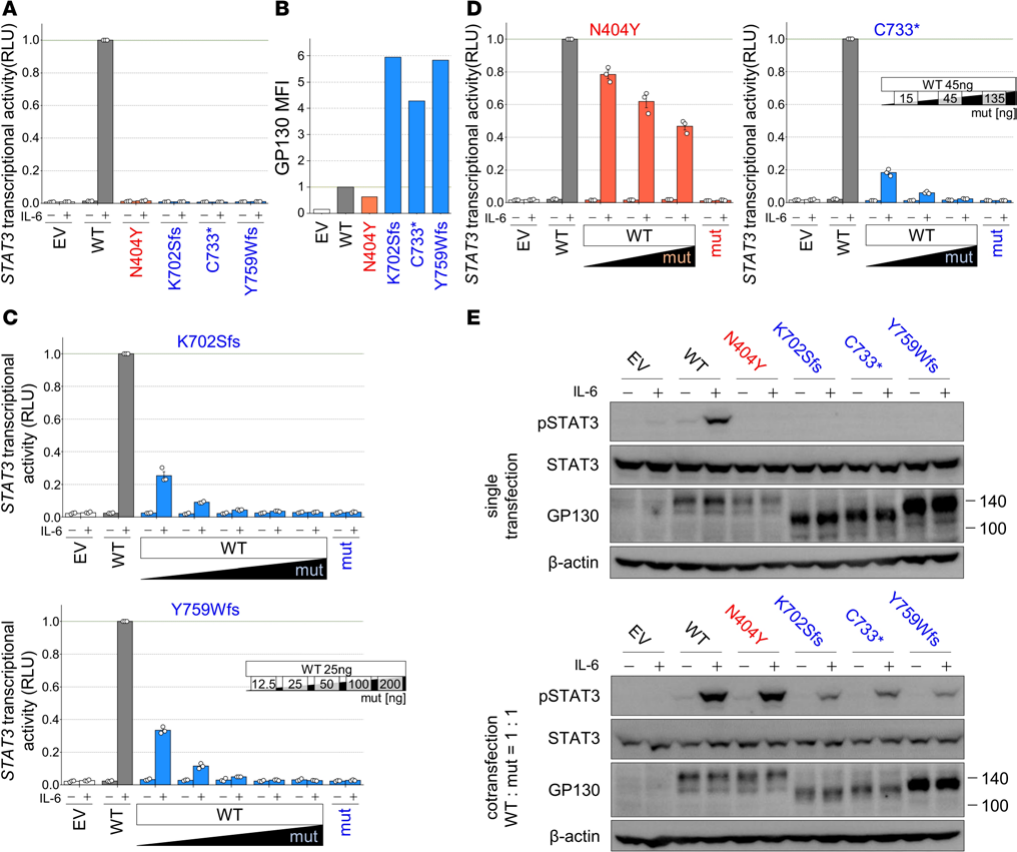
The 2 previously unreported variants were LOF variants and accumulate on the cell surface and have strong DN effects. (**A** and **C**–**D**) STAT3 transcriptional activity in GP130-KO-HEK293T cells transfected with WT and/or variants and stimulated with or without IL-6. In **C** and **D**, the amount of transfected DNA is shown on the right. RLU values were normalized to the poststimulation WT values as 1. Data are shown as mean ± SEM of technical triplicates. This experiment was independently performed 3 times, and a representative result is shown. Red, EC variants; blue, IC variants. (**B**) MFI of cell-surface GP130 expression in transfected GP130-KO-HEK293T cells. MFI values were normalized to the WT values as 1. This experiment was independently performed 3 times, and a representative result is shown. (**E**) Immunoblotting of pSTAT3, STAT3, and GP130 in GP130-KO-HEK293T cells transfected with WT and/or variants and stimulated with or without IL-6 (left, single; right, cotransfection at a 1:1 ratio). β-Actin was used as a loading control. This experiment was independently performed twice, and a representative result is shown. EV, empty vector; mut, mutant.

We conducted functional assessments to evaluate the STAT3 signaling capacity of the *IL6ST* variants using luciferase reporter assays. GP130-deficient HEK293T cells (abbreviated as GP130-KO-HEK293T cells) were cotransfected with plasmids encoding either EV, WT *IL6ST*, 2 of our previously unreported *IL6ST* variants (p.K702Sfs, p.Y759Wfs), a reported AR-*IL6ST* mutation (p.N404Y) (12), or a reported AD-*IL6ST* mutation (p.C733*) (15), along with reporter plasmids. In WT GP130-expressing cells, STAT3 transcriptional activity increased in a dose-dependent manner upon IL-6 stimulation (Supplemental Figure 4). In contrast to those expressing the WT, cells expressing the p.K702Sfs and p.Y759Wfs variants, along with known *IL6ST* mutants (p.N404Y and p.C733*), exhibited no activity (15, 16) (Figure 4A). The 2 previously unreported variants, as well as reported mutants that accumulated on the cell surface, lacked the recycling motif. To validate this phenomenon, we measured the cell-surface GP130 levels of the transfected GP130-KO-HEK293T cells by flow cytometry. Cell-surface GP130 expression was observed in WT GP130-expressing cells and was equivalent to that of the p.N404Y mutant. In contrast, the p.K702Sfs and p.Y759Wfs variants, as well as the p.C733* mutant, exhibited excessive cell-surface GP130 accumulation (Figure 4B). These findings demonstrate that the p.K702Sfs and p.Y759Wfs variants not only exhibit a complete loss of STAT3 transcriptional activity but also accumulate GP130 on the cell surface.

### The p.K702Sfs and p.Y759Wfs variants exhibit DN effects.

We further investigated whether the p.K702Sfs and p.Y759Wfs variants exhibit DN effects. GP130-KO-HEK293T cells were cotransfected with a constant amount of WT and various amounts of variant GP130-encoding plasmids and were subjected to a luciferase reporter assay. Both the p.K702Sfs and p.Y759Wfs variants exhibited strong DN effects, similar to those of the p.C733* DN mutants. In contrast, the p.N404Y mutant, which causes AR GP130 deficiency, did not show an obvious DN effect (Figure 4, C and D, and Supplemental Figure 5). These results are consistent with those described in previous reports (15, 16). We then confirmed whether these DN effects were related to impaired STAT3 phosphorylation. GP130-KO-HEK293T cells were transfected with WT or variant GP130-encoding plasmids at a 1:1 ratio. Proteins were then extracted and subjected to immunoblotting. Cells expressing the p.N404Y mutant showed a comparable level of STAT3 phosphorylation in response to IL-6 as those expressing the WT alone, whereas cells expressing the p.K702Sfs and p.Y759Wfs variants, along with the p.C733* mutants, exhibited decreased STAT3 phosphorylation. Therefore, both the p.K702Sfs and p.Y759Wfs variants have been confirmed to be pathogenic mutations with DN effects on STAT3 transcriptional activity and impaired STAT3 phosphorylation (Figure 4E).

### Cell-surface GP130 expression and the response to IL-6 in patient cells.

**Figure 5:**
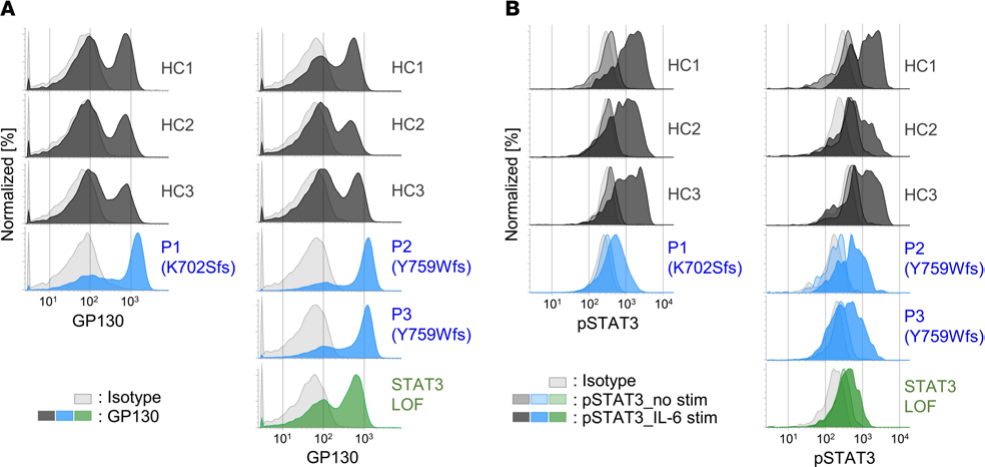
Patient PBMCs show cell-surface GP130 accumulation and impaired phosphorylation of STAT3. (**A**) Cell-surface GP130 expression in PBMCs from patients and healthy individuals. (**B**) PBMCs from patients and healthy individuals were cultured and stimulated with IL-6 or left unstimulated. The expression of phosphorylated STAT3 (pSTAT3) gated with CD14 in the collected cells after fixation and permeabilization is shown as a histogram. The horizontal axis of the histogram is plotted on a logarithmic scale. Each experiment was independently performed twice, and a representative result is shown. HC, healthy controls; LOF, loss of function.

We next assessed the accumulation of GP130 on the cell surface of patient peripheral blood mononuclear cells (PBMCs) (Supplemental Figure 6A). The PBMCs from P1, P2, and P3 exhibited greater GP130 accumulation than did those from healthy controls (HCs) (Figure 5A and Supplemental Figure 6B). After stimulation with IL-6, we observed impaired STAT3 phosphorylation in CD14^+^ cells among PBMCs from P1, P2, and P3. This impairment of STAT3 phosphorylation in CD14^+^ cells from P2 and P3 was less severe than that in CD14^+^ cells from a patient with HIES who carried a heterozygous DN mutation, p.V637M, in *STAT3* (Figure 5B and Supplemental Figure 6C). Therefore, we confirmed the cell surface accumulation of GP130 and impairment of STAT3 phosphorylation in the cells of patients with AD GP130 deficiency.

### Identification of the position distinguishing between AR- and AD-IL6ST mutations with a premature stop codon.

**Figure 6:**
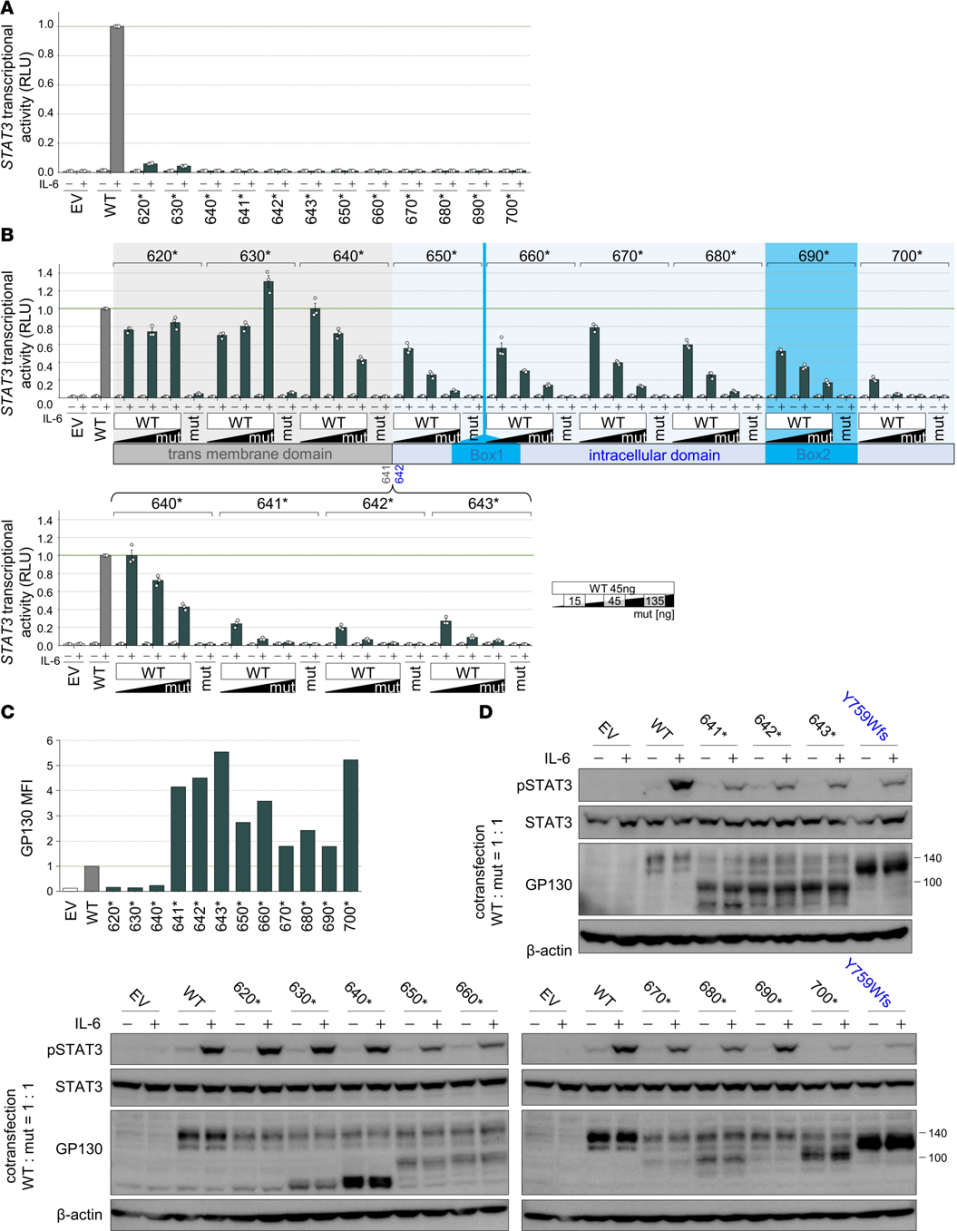
A C-terminal nonsense mutant from the aa F641 enhances the DN effect. (**A** and **B**) STAT3 transcriptional activity in GP130-KO-HEK293T cells transfected with WT and/or mutants and stimulated with or without IL-6. In **B**, the amount of transfected DNA is shown on the lower right. RLU values were normalized to the poststimulation WT values as 1. Data are shown as mean ± SEM of technical triplicates. This experiment was independently performed 3 times, and a representative result is shown. (**C**) MFI of cell-surface GP130 expression in transfected GP130-KO-HEK293T cells. MFI values were normalized to the WT values as 1. This experiment was independently performed 3 times, and a representative result is shown. (**D**) Immunoblotting of pSTAT3, STAT3, and GP130 in GP130-KO-HEK293T cells transfected with WT and mutants at a 1:1 ratio and stimulated with or without IL-6. β-Actin was used as a loading control. This experiment was independently performed twice, and a representative result is shown. EV, empty vector; mut, mutant.

Reported AR-*IL6ST* mutations cluster in the EC, while reported AD-*IL6ST* mutations with DN effects preferentially localize in the IC upstream of the recycling motif (Figure 1). Therefore, we hypothesized that there might be a boundary distinguishing between AR- and AD-*IL6ST* mutations. Since the majority of mutations identified in patients with GP130 deficiency are nonsense or frameshift mutations that create a premature stop codon, we systematically generated mutants with a stop codon every 10 aa in the hypothetical region (these mutants are abbreviated as, e.g., A620*→620*). We then performed luciferase reporter assays to evaluate the IL-6–induced STAT3 transcriptional activity of these mutants. Cells expressing the 620* or 630* mutants showed residual activity, while the other downstream mutants demonstrated complete loss of activity (Figure 6A). We next performed cotransfection with a constant amount of WT and various amounts of mutants to investigate whether these mutants have a DN effect. Clear DN effects were observed in cells expressing the mutant from 650* to the C-terminal region (Figure 6B). For further precise analysis, we created mutants with stop codons for every aa around C640. Clear DN effects were observed in cells expressing mutants on the C-terminal side of the 641* mutation by a reporter assay (Figure 6B and Supplemental Figure 7). Furthermore, flow cytometry and immunoblotting confirmed cell-surface GP130 accumulation (Figure 6C) and reductions in IL-6–induced STAT3 phosphorylation (Figure 6D) in these cells. The 640* mutation demonstrated a dose-dependent decrease in activity with an unclear DN effect, similar to a haploinsufficiency model like N404Y, whereas the 630* mutation did not show any DN effect. Further analysis revealed that mutations located N-terminal to 639* did not exhibit the same phenomenon observed with 640*, and accumulation of GP130 was not observed (Supplemental Figure 8, A–D). Taken together, these findings reveal strong DN effects, cell-surface GP130 accumulation, and impaired IL-6–induced STAT3 phosphorylation at the border between the 640* and 641* mutations, indicating that the boundary between AR- and AD-*IL6ST* mutations might exist in this region. Moreover, these results suggest that cell surface accumulation of mutant GP130, coupled with decreased IL-6–induced STAT3 transcriptional activity, may lead to strong DN effects.

### Accumulation of mutant GP130 on the cell surface enhances DN effects.

**Figure 7:**
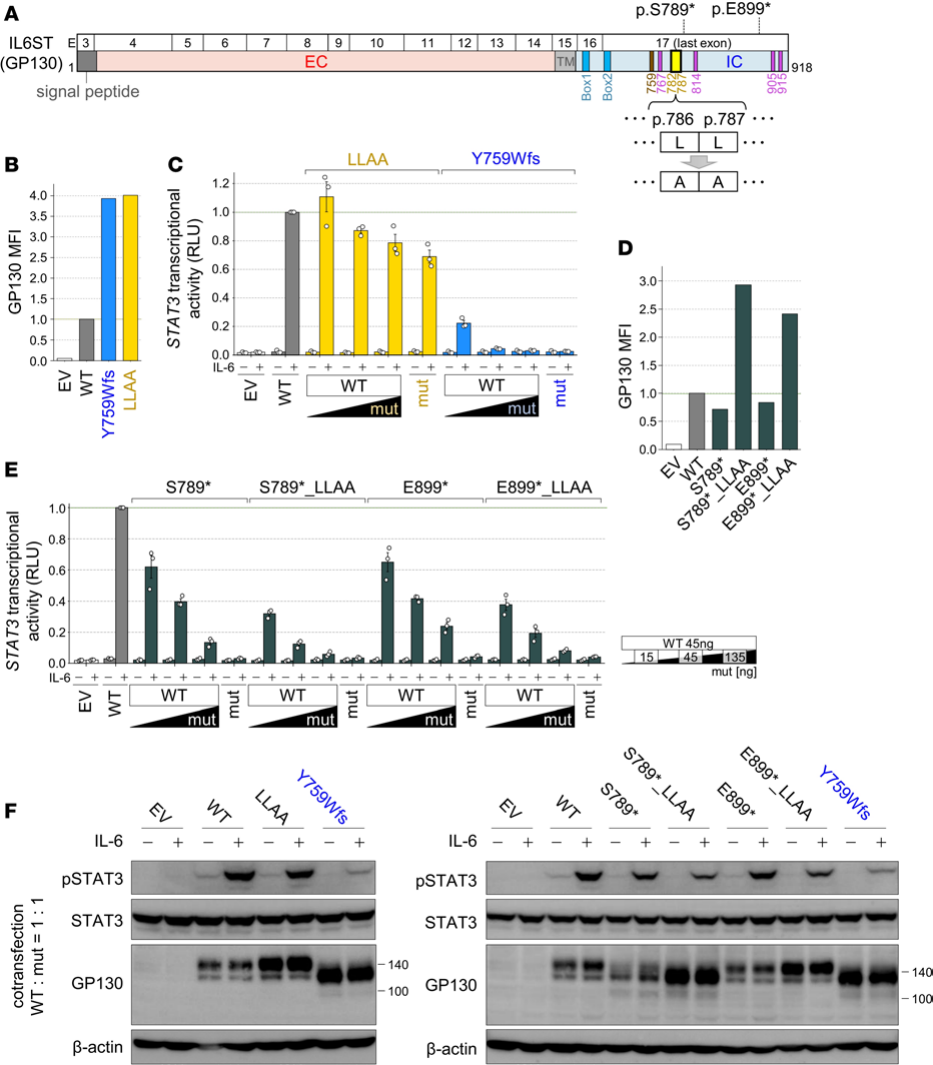
Absence of the dileucine motif causes GP130 to accumulate on the cell surface, enhancing the DN effect. (**A**) Schematic of GP130 and image of the position of the di-leucine motif and alanine substitution, with the 2 mutants described in gnomAD (p.S789*, p.E899*). (**B** and **D**) MFI of cell-surface GP130 expression in transfected GP130-KO-HEK293T cells. MFI values were normalized to the WT values as 1. This experiment was independently performed 3 times, and a representative result is shown, with Y759Wfs shown as a patient control. (**C** and **E**) STAT3 transcriptional activity in GP130-KO-HEK293T cells transfected with WT and mutants at various ratios and stimulated with or without IL-6. RLU values were normalized to the poststimulation WT values as 1. Data are shown as mean ± SEM of technical triplicates. This experiment was independently performed 3 times, and a representative result is shown. The amount of transduced DNA is shown on the right. (**F**) Immunoblotting of pSTAT3, STAT3, and GP130 in GP130-KO-HEK293T cells transfected with WT and mutants at a 1:1 ratio and stimulated with or without IL-6. β-Actin was used as a loading control. This experiment was independently performed twice, and a representative result is shown. E, exon; EV, empty vector; mut, mutant.

Two leucine residues (a di-leucine motif), at aa positions 786 and 787, within the recycling motif sequence have been reported to be crucial for GP130 endocytosis (17). We created variants in which the di-leucine motif of WT GP130 was replaced with alanine (LL786-787AA [LLAA]) and analyzed their function (Figure 7A). Cells expressing the LLAA variant, as well as the p.Y759Wfs mutant, exhibited the cell-surface GP130 accumulation (Figure 7B). IL-6–induced STAT3 transcriptional activity was not markedly affected in cells expressing the LLAA mutant, whereas the p.Y759Wfs mutant exerted a strong DN effect (Figure 7C and Supplemental Figure 7). This result suggests that the accumulation of functional GP130 itself does not impair IL-6–induced STAT3 transcriptional activity. Next, we substituted di-leucine motifs with alanines in 2 nonsense mutants, the p.S789* (with one STAT3 binding site) and p.E899* (with 2 STAT3 binding sites) variants (abbreviated as S789*_LLAA and E899*_LLAA, respectively), which are reported in gnomAD v4.0.0. In a previous report, due to the retention of 1 or 2 STAT3 binding sites and the recycling motif, these 2 variants were shown to be severe hypomorphs and DN but at weaker levels than LOF mutants lacking all 4 STAT3 binding sites and the recycling motif (15). Both S789*_LLAA and E899*_LLAA variant–expressing cells exhibited increased cell-surface GP130 accumulation (Figure 7D), and their DN effects were markedly enhanced compared with those of p.S789* and p.E899* variants, respectively (Figure 7E and Supplemental Figure 7). Additionally, a slight reduction in STAT3 phosphorylation was also observed in cells expressing the p.S789* and p.E899* variants by substitution of the di-leucine motif with alanines (Figure 7F). These results demonstrate that the accumulation of mutant GP130 leads to a stronger DN effect.

### Analysis of nonsense or frameshift variants listed in gnomAD and UK Biobank.

Since several nonsense or frameshift variants in the IC are registered in gnomAD and UK Biobank (Supplemental Table 2), these variants were also analyzed. Except for cells expressing the p.Q918* and p.*919Efs16 variants, which were hypomorphic, all variants were LOF for IL-6 signaling (Supplemental Figure 9A). For variants up to p.S731Vfs*8 lacking the recycling motif, DN effects were observed, except for p.H650Tfs*5 and p.H724Tfs*15 variants. Among the variants from p.S789* onward retaining the recycling motif, p.S789*, p.K849Tfs*2, and p.E899* exhibited DN effects equal to or greater than the pathogenic p.S731Vfs*8 (16), while no marked DN effects were observed in the others (Supplemental Figure 9B). For variants up to p.S731Vfs*8 lacking the recycling motif, p.H650Tfs*5, p.K716Rfs*72, p.H724Tfs*15, and p.S731Vfs*8 did not show clear cell-surface GP130 accumulation, but a correlation between the cell-surface GP130 accumulation and the DN effects was observed (Supplemental Figure 9C). Additionally, a reduction in STAT3 phosphorylation correlated with the DN effect (Supplemental Figure 9D). There was no mention of HIES symptoms in patients registered in the UK Biobank (p.R644*, p.S678*, p.M867Cfs*19, p.Q918*) (data not provided).

## Discussion

We herein report, to the best of our knowledge, the first 3 East Asian patients with AD HIES caused by potentially novel *IL6ST* DN mutations. Among reported AD-*IL6ST* mutations, the p.K702Sfs mutation found in P1 was located closest to the TM (15, 16). p.Y759Wfs represents a frameshift mutant in which the SHP2/SOCS3 binding site was removed. The clinical phenotype was similar to that of previously reported cases (15, 16). Notably, patients with the p.K702Sfs (P1) mutation were less susceptible to infections. However, it is worth mentioning that excessive cell-surface GP130 accumulation was also observed in these patients’ PBMCs. Although it was suggested that the immune phenotype might be correlated with cell-surface GP130 accumulation (16), the findings observed in patients with the p.K702Sfs mutation imply that the immune phenotype cannot be attributed solely to cell-surface GP130 accumulation.

We hypothesized the existence of a mutational boundary between AR and AD GP130 deficiency based on the distribution of these mutants. Our analysis of GP130-KO-HEK293T cells transfected with mutant GP130 revealed cell-surface GP130 accumulation and the emergence of DN effects with nonsense mutants positioned at the C-terminal side of F641. These results suggest that patients with a mutation in *IL6ST*, specifically a heterozygous nonsense or frameshift mutation positioned downstream from the aa F641, may exhibit AD HIES. This discovery potentially facilitates the evaluation of the functional relevance of identified nonsense or frameshift variants in *IL6ST* based on their position.

As described above, the majority of mutants located on the C-terminal side from the aa F641 exhibited both excessive cell-surface GP130 accumulation and DN effects. These results suggest that we can estimate the pathogenicity of previously unreported variants in *IL6ST* by simply analyzing cell-surface GP130 accumulation in patient PBMCs. In contrast, Arlabosse et al. intriguingly reported a unique mutant, p.S731Vfs*8, in which cell-surface GP130 accumulation was either normal or only slightly greater in patient PBMCs (16). The mechanism underlying the lack of strong accumulation in this mutant remains to be characterized. The di-leucine motif is a well-known aa sequence involved in the internalization of membrane proteins (17, 21, 22). Serines located upstream of the di-leucine motif are also known to be involved in endocytosis (17, 23, 24). Additionally, there are various types of recycling motifs that can lead to endocytosis (25–28). Interestingly it has also been reported that the di-leucine motif within a cleaved protein positioned just upstream of the cleavage point restores endocytic activity in other receptor proteins (29). Sequences that appear in the tail of the cleaved protein may contribute to restoring endocytosis and preventing the accumulation of cell-surface GP130. These mechanisms might explain the slightly weaker cell-surface GP130 accumulation in some variants (p.K716Rfs*72 and p.S731Vfs*8), including the disease-causing p.S731Vfs*8, which causes milder forms of HIES with considerable clinical heterogeneity among carriers. Therefore, while it may be challenging to rule out AD GP130 deficiency solely based on the absence of cell-surface GP130 accumulation in PBMCs, the detection of excessive cell-surface GP130 accumulation in patients with HIES strongly supports the diagnosis of AD GP130 deficiency.

Béziat et al. reported 2 potential mechanisms for the strong DN effect of mutant GP130 (15). (a) GP130, IL6R, and IL-6 form a 2:2:2 hexamer. Four combinations of dimers are formed by WT and mutant GP130 (WT/WT, WT/mut, mut/WT, and mut/mut). Among these combinations, 3 are considered LOF combinations, except WT/WT under heterozygous conditions. (b) Mutant GP130 accumulates on the cell surface due to the loss of recycling motifs. In this study, to investigate the latter mechanism, we generated several variants with impaired endocytosis activity. We observed that cell-surface GP130 accumulation in the S789*_LLAA and E899*_LLAA variants enhanced their DN effects compared with that of the same hypomorphic variants without mutations in the recycling motif. This finding demonstrates that the strength of the DN effect was related not only to the involvement of the variant GP130 in hexamer formation but also to the accumulation of the variant GP130 on the cell surface.

The results of transient expression experiments revealed that cell-surface GP130 accumulation occurs in nonsense mutants downstream of the aa F641. Therefore, this aa was suspected to be the boundary between AR- and AD-*IL6ST* mutations. However, this position is located more than 50 bases upstream from the last exon junction and is theoretically affected by NMD (19). It is surprising that 11 HIES-causing mutations, identified in 23 patients, were reported within 66 aa (aa 702–768), but none in the 61 between TM and Box2 (aa 641–702). It remains possible that variants resulting in the loss of Box2 (aa 641–700) do not underlie HIES or only underlie a mild form of the disease. Indeed, we found that a truncation at aa 700 was more dominant negative than the one at aa 690 (Figure 6B).

Additionally, gnomAD v4.0.0 and the UK Biobank include IC nonsense or frameshift variants. Based on our hypothesis, some of these variants may be pathogenic. In our experiments, mutations up to p.S731Vfs*8, which lack the recycling motif, exhibited DN effects and cell-surface GP130 accumulation in most cases, although the effects were somewhat milder in the p.K716Rfs*72 and p.S731Vfs*8 variants. In contrast, the p.H650Tfs*5 and p.H724Tfs*15 variants did not show GP130 accumulation or DN effects. In the p.H650Tfs*5 variant, despite the increased expression of intracellular GP130 detected by immunoblotting, no accumulation was observed on the cell surface, suggesting that it did not reach the cell surface through some mechanism. The protein of the p.H650Tfs*5 variant was smaller than that of the p.R644* variant, implying that it might be influenced by posttranslational modifications or other factors. In the p.K716Rfs*72 and p.S731Vfs*8 variants, protein expression was either reduced or slightly reduced compared with that of the WT, with mild GP130 accumulation and detectable DN effects. As mentioned in the previous paragraph, there may be some mechanisms that prevent GP130 accumulation. If there were a method to detect the ratio of WT and mutant GP130 on the cell surface, it might provide more valuable information. For the p.H724Tfs*15 variant, GP130 expression was barely detectable by immunoblotting, suggesting that it was either unstable or targeted for degradation. Further research on molecular mechanisms will be necessary to elucidate these findings. In any case, all of the variants showed a correlation between DN effects and cell-surface GP130 accumulation, reinforcing our hypothesis that pathogenicity can be determined by assessing cell-surface GP130 accumulation of patient cells. Additionally, among the mutations from p.S789* onward that retain the recycling motif, p.S789*, p.K849Tfs*2, and p.E899* showed a tendency toward DN effects, while no marked DN effects were observed in the others. For variants retaining the recycling motif and having some or all of the STAT3 binding sites remaining, the mechanism of residual activity is more complex. Indeed, the p.S731Vfs*8 mutation reported in patients with HIES is also listed in gnomAD v4.0.0 and was associated with a heterogenous phenotype among carriers (16). In addition, there are cases like P3 with long-undiagnosed conditions. Thus, individuals with DN variants after the recycling motif may exhibit HIES symptoms. Therefore, the pathogenicity of the variants cannot be simply dismissed based on their registration in a public database. For patients with mutations likely to cause AD GP130 deficiency, an accurate assessment of pathogenicity requires a detailed investigation of HIES symptoms and, ultimately, functional evaluation of the mutants using patient cells. Additionally, this study investigated only the response to IL-6, and further insights may be gained by examining responses to other cytokines.

In summary, we reported 2 previously unreported mutations associated with AD GP130 deficiency resulting in HIES. The p.K702Sfs mutation is located closer to the TM than any previously reported AD-*IL6ST* mutation that generates a premature stop codon. This patient exhibited phenotypes similar to those described earlier, albeit with milder infectious symptoms. We also identified an aa that could serve as the boundary distinguishing between AR- and AD-*IL6ST* mutations. Moreover, we demonstrated the potential utility of detecting cell-surface GP130 accumulation in diagnosing AD GP130 deficiency in patients with HIES with an unknown variant in *IL6ST*.

## Methods

### Sex as a biological variable.

Our study included both male and female participants. Sex was not considered as a biological variable.

### Genetic analysis.

Genomic DNA was extracted from whole blood samples collected from P1 (family 1) and P2 (family 2). WES was performed on an Illumina NextSeq 2000. The obtained fastq data were preprocessed for quality control and other steps and aligned to the hg19 reference human genome using BWA. Variant calling was performed using SAMtools, Genome Analysis Toolkit (GATK; MarkDuplicates, BQSR, ApplyBQSR, HaplotypeCaller), the resulting variant call format (VCF) files were annotated, and variant analysis was performed. Targeted Sanger sequencing of the mutations was performed for the patient’s family.

### Deep immunophenotyping analysis using the Aurora (Cytek).

Deep immunophenotyping using Aurora was performed on PBMCs obtained from patients P2 and P3. The gating strategy was well established in previous studies (30–32), and we adopted it. Details of the antibodies and reagents used are provided in Supplemental Table 3.

### Generation of cell lines and cell culture.

HEK293T cells derived from the human embryonic kidney 293 cell line. We generated GP130-KO-HEK293T cells using the CRISPR/Cas9 system, as described in a previous study (15). The generated cell line was tested for impaired GP130 expression. GP130-KO-HEK293T cells were cultured in DMEM supplemented with 10% FBS. PBMCs were obtained from whole blood samples of patients and HCs. PBMCs were cultured in RPMI 1640 supplemented with 20% FBS before the experiment.

### Plasmids and transient transfection for overexpression experiments.

Human *IL6ST* expression vectors (RC215123; OriGene) and a C-terminal Myc/DDK-tagged pCMV6 empty vector (EV) were used. The creation of constructs with all variant alleles was performed as follows: mutagenesis of the human *IL6ST* expression vectors was performed using paired primers, and ECOS-competent *E*. *coli* DH5α (NIPPON GENE) cells were transformed with the constructs, followed by culture in liquid LD broth (MilliporeSigma) and purification using the Plasmid DNA Maxiprep Kit (Thermo Fisher Scientific). A stop codon was inserted between the *IL6ST* cDNA and the Myc/DDK tag of these *IL6ST* expression vectors. The generated plasmids were transfected into GP130-KO-HEK293T cells using X-tremeGENE 9 DNA Transfection Reagent (Roche).

### Luciferase reporter assay.

GP130-KO-HEK293T cells were seeded in 96-well plates 24 hours before transfection with DMEM supplemented with 10% FBS. In the functional assay, the cells were transfected with the pGL4.47{luc2P/SIE/Hygro} (Promega) reporter plasmid (100 ng/well), the pRL-SV40 vector (40 ng/well), and either the WT or variant pCMV6-GP130 plasmid (100 ng/well) using X-tremeGENE 9 DNA Transfection Reagent (Roche). To evaluate the DN effect of variant GP130, GP130-KO-HEK293T cells were transfected with the pGL4.47{luc2P/SIE/Hygro} (Promega) reporter plasmid (100 ng/well), the pRL-SV40 vector (40 ng/well), a constant amount of the WT pCMV6-GP130 plasmid, and various concentrations of the variant pCMV6-GP130 plasmid (WT/mut = 25 ng/well: 12.5, 25, 50, 100, 200 ng/well, or WT/mut = 45 ng/well: 15, 45, 135 ng/well) using X-tremeGENE 9 DNA Transfection Reagent (Roche). EV was added to adjust the total amount of DNA transfected into the cells. Cells transfected with the pGL4.47{luc2P/SIE/Hygro}(Promega) reporter plasmid, the pRL-SV40 vector with the WT pCMV6-GP130 plasmid only, the variant pCMV6-GP130 plasmid only, or the EV only were also included as controls. After 24 hours of transfection, the medium was replaced, and the cells were cultured for another 24 hours in DMEM supplemented with 10% FBS with or without IL-6 (100 ng/mL). Luciferase assays were performed with a Dual-Glo luciferase assay system (Promega), and promoter activity in each well was evaluated by determining the ratio of firefly luciferase activity to Renilla luciferase activity (RLU). Values for all data points in graphs are reported in the Supporting Data Values file.

### Flow cytometry of GP130.

GP130-KO-HEK293T cells were seeded in 48-well plates 24 hours before transfection in DMEM supplemented with 10% FBS. The cells were transfected with the WT or variant pCMV6-GP130 plasmid (200 ng/well) using X-tremeGENE 9 DNA Transfection Reagent (Roche). Forty-eight hours after transfection, the cells were detached and collected using Cell Dissociation Buffer Enzyme-free PBS-based (Thermo Fisher Scientific), washed with FACS buffer (PBS[–]+2% FBS), and stained by incubating for 20 minutes at room temperature with Alexa Fluor 647 mouse anti–human CD130 antibody (564151; BD Biosciences) in the same buffer. PBMCs were stained in the same manner as described above. Cell fluorescence intensity data were acquired and analyzed by FACSVerse (BD Biosciences).

### Immunoblotting.

GP130-KO-HEK293T cells were seeded in 12-well plates 24 hours before transfection in DMEM supplemented with 10% FBS. Equal amounts of the WT and variant pCMV6-GP130 plasmids were transfected either alone or in combination (for a total of 1,500 ng/well) using X-tremeGENE 9 DNA Transfection Reagent (Roche). Twenty-four hours after transfection, IL-6 (50 ng/mL) was added to the medium, after which the cells were stimulated and incubated at 37°C for 15 minutes. The medium was then quickly removed, and PBS(–) + phosphatase inhibitor cocktail (100×, Thermo Fisher Scientific) + protease inhibitor cocktail (100×, Thermo Fisher Scientific) was added. Cells were collected from all wells by pipetting. RIPA buffer (MilliporeSigma) + Phosphatase Inhibitor Cocktail (100×, Thermo Fisher Scientific) + Protease Inhibitor Cocktail (100×, Thermo Fisher Scientific). The protein extract was dissolved in SIGMA buffer (MilliporeSigma), incubated at 4°C for 30 minutes, and centrifuged at 20,000*g* for 20 minutes to obtain the protein extract. Each protein extract was quantified using the BCA Protein Assay Kit (Thermo Fisher Scientific). Approximately 15 μg of protein was separated by SDS-PAGE and immunoblotted using antibodies against pSTAT3 (9145; Cell Signaling Technology), STAT3 (9139; Cell Signaling Technology), GP130 (E-8; Santa Cruz Biotechnology Inc.), and β-actin (A5316; MilliporeSigma).

### Phospho-flow cytometry of pSTAT3.

After thawing, the PBMCs were allowed to rest in RPMI supplemented with 20% FBS for 2 hours at 37°C. The cell suspension was centrifuged at 200*g* for 5 minutes, and FBS-free RPMI was added to reach a final concentration of 1.0 × 10^7^ cells/mL. Then, 100 μL of FITC mouse anti–human CD14 antibody (347493; BD Biosciences) was added, and IL-6 (50 ng/mL) was added simultaneously to the cells for stimulation, followed by incubation at 37°C for 15 minutes. After stimulation, fixation buffer (BD Biosciences) was added at room temperature for 10 minutes to fix the cells. Then, after centrifugation at 300*g* for 3 minutes and discarding the supernatant, Perm Buffer III (BD Biosciences) was added, and the mixture was incubated on ice for 15 minutes for permeabilization. The cells were then washed with FACS buffer and stained with Alexa Fluor 647 mouse anti-Stat3 (pY705) (557815; BD Biosciences) in the same buffer by incubation at room temperature for 20 minutes. Cell fluorescence intensity data were acquired and analyzed by FACSVerse (BD Biosciences).

### Statistics.

Experiments performed in technical triplicates are presented as mean values with data shown as mean ± SEM. When experiments were independently repeated, the number of replicates is indicated in the figure legends. Two-tailed Mann-Whitney *U* tests were used for single comparisons of independent groups. Statistical analyses were performed using GraphPad Prism software. A *P* value less than 0.05 was considered statistically significant.

### Study approval.

Informed consent was obtained from all patients involved in this study. The study was approved by the IRB of Hiroshima University (approval no. E2014-9126).

### Data availability.

The exome sequencing data generated and analyzed in this study are derived from patients and cannot be publicly shared due to ethical and privacy restrictions. Deidentified data may be made available upon reasonable request from the corresponding author, subject to relevant approvals. Values for all data points in graphs are reported in the Supporting Data Values file.

## Author contributions

KA, TA, and SO designed the study. KA, TA, and SO wrote the manuscript. KA, WW, and KT performed the experiments. KI, ET, AS, TK, and HH provided clinical input. WTL and CLK performed deep immunophenotyping analysis using the Aurora (Cytek). AC and CC conducted the analysis of the UK Biobank data. KN, MT, YM, SK, DB, JLC, and VB provided helpful discussions. All the authors critically reviewed the manuscript.

## Supplementary Material

Supplemental data

Unedited blot and gel images

Supporting data values

## Figures and Tables

**Table 1:**
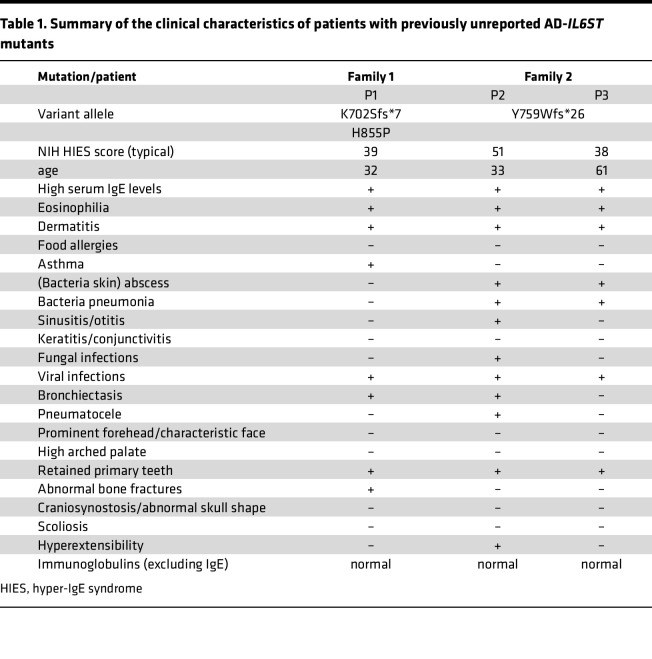
Summary of the clinical characteristics of patients with previously unreported AD-*IL6ST* mutants

## Version 1 06/17/2025

In-Press Preview

## Version 2 07/22/2025

Electronic publication
