# Supplementary material for: Fine mapping of heterozygous IL6ST nonsense variants underlying autosomal dominant hyper-IgE syndrome
Source: JCI Insight. 2025 Jun 17;10(14):e190065. doi: 10.1172/jci.insight.190065 (PMC12288962; doi:10.1172/jci.insight.190065)
Supplement: Supplemental data [file jciinsight-10-190065-s162.pdf]

Supplemental figures and tables

Figure S1. Schematic representation of GP130 and IL-6 family cytokine signaling.

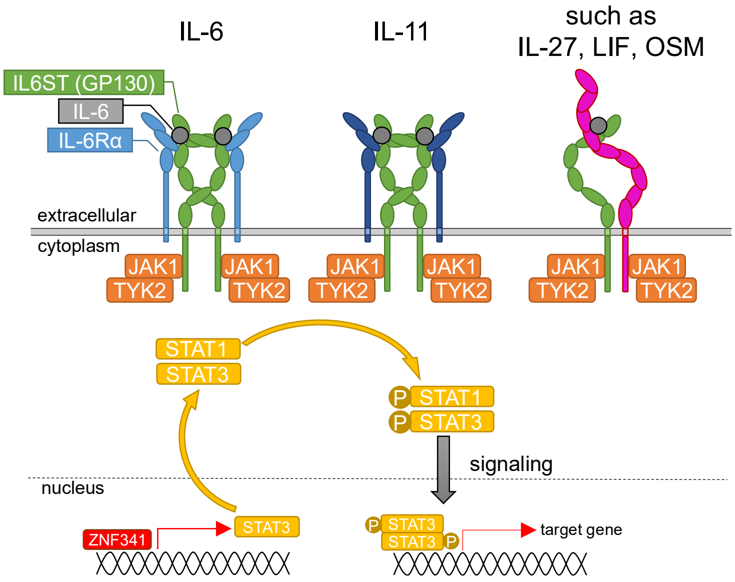

IL-6, the IL-6 receptor, and GP130 form a hexamer at the cell surface, with two molecules each. The same is true for IL-11, while IL-27, LIF, and OSM form a trimer with one molecule each. This leads to signaling to the nucleus through phosphorylation of Jaks and STATs, resulting in the expression of a variety of genes. STAT3 expression is regulated by ZNF341.

Figure S2. The p.H855P mutation is an allele identical to p.K702fs.

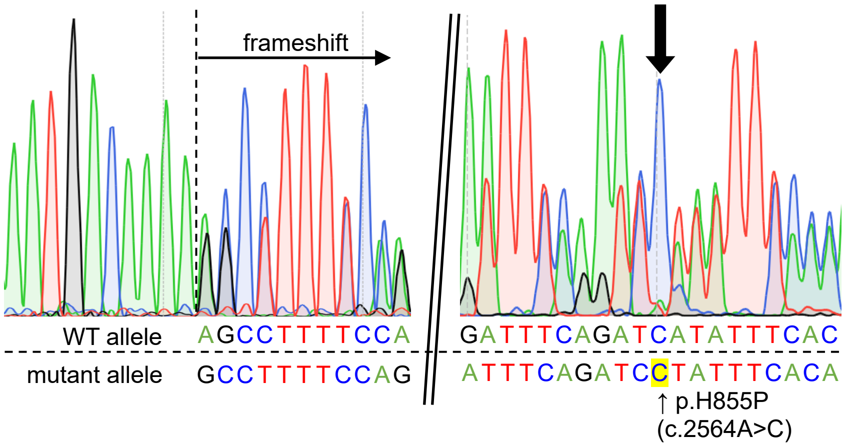

The waveform pattern of the Sanger sequence indicated that p.H855P and p.K702fs are located on the same allele. If they were on different alleles, the waveforms (black arrows) would be in the A and C heteropattern.

Figure S3. Deep immunophenotyping of patients with AD-*IL6ST* mutation Y759Wfs.

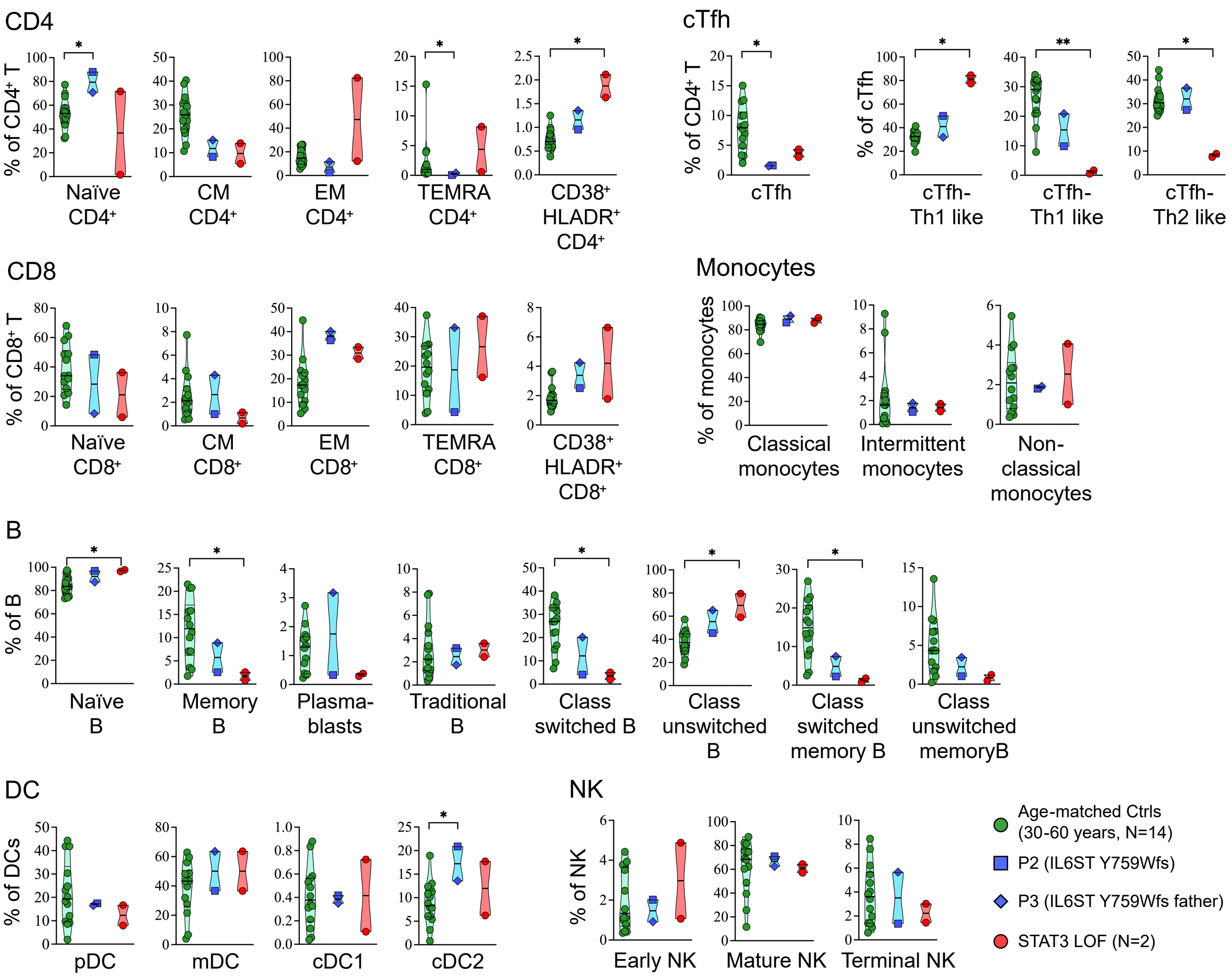

This deep immunophenotyping for patient PBMCs was performed using Aurora (Cytek). Age-matched healthy controls (30-60 years, N=14) are represented by green dots, P2 by blue squares, P3 by blue diamonds, and the patients with heterozygous DN *STAT3* mutation patients (p.V637M, p.Y657C) by red dots (N=2). Statistical analysis was done with two-tailed Mann–Whitney U test. *; P<0.05, **; P<0.01. CM, central memory; EM, effector memory; TEMRA, terminally differentiated effector memory T cells re-expressing CD45RA; cTfh, circulating follicular helper T; pDC/mDC/cDC, plasmacytoid/monocyte-derived/conventional dendritic cell.

Figure S4. Determination of the amount of WT plasmid to be used in the experiments.

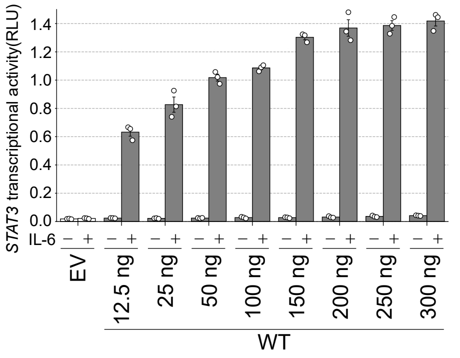

STAT3 transcriptional activity of GP130-KO-HEK293T cells transfected with several amounts of WT, and EV was added to adjust the total amount of DNA transfected into the cells. Bars and error bars are the means and SEM of technical triplicates. This experiment was independently performed three times, and a representative result is shown. EV, empty vector; mut, mutant.

Figure S5. The hetero 2 variants (K702fs, Y759Wfs) are less active than haploinsufficiency due to the DN effect.

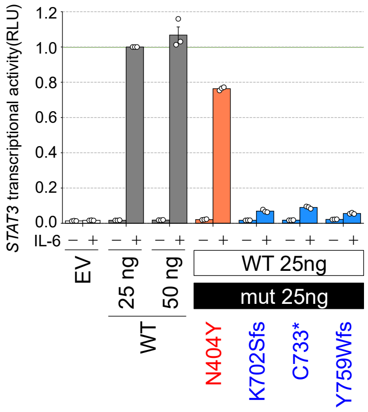

STAT3 transcriptional activity of GP130-KO-HEK293T cells transfected with either WT (25 ng or 50 ng) or WT and variants at a 1:1 ratio (WT:mut = 25 ng:25 ng) and stimulated with or without IL-6. RLU values were normalized to the post-stimulation WT (25 ng) values as 1. Bars and error bars are the means and SEM of technical triplicates. This experiment was independently performed three times, and a representative result is shown. EV, empty vector; mut, mutant.

Figure S6. Patient PBMCs show cell surface GP130 accumulation and impaired STAT3 phosphorylation.

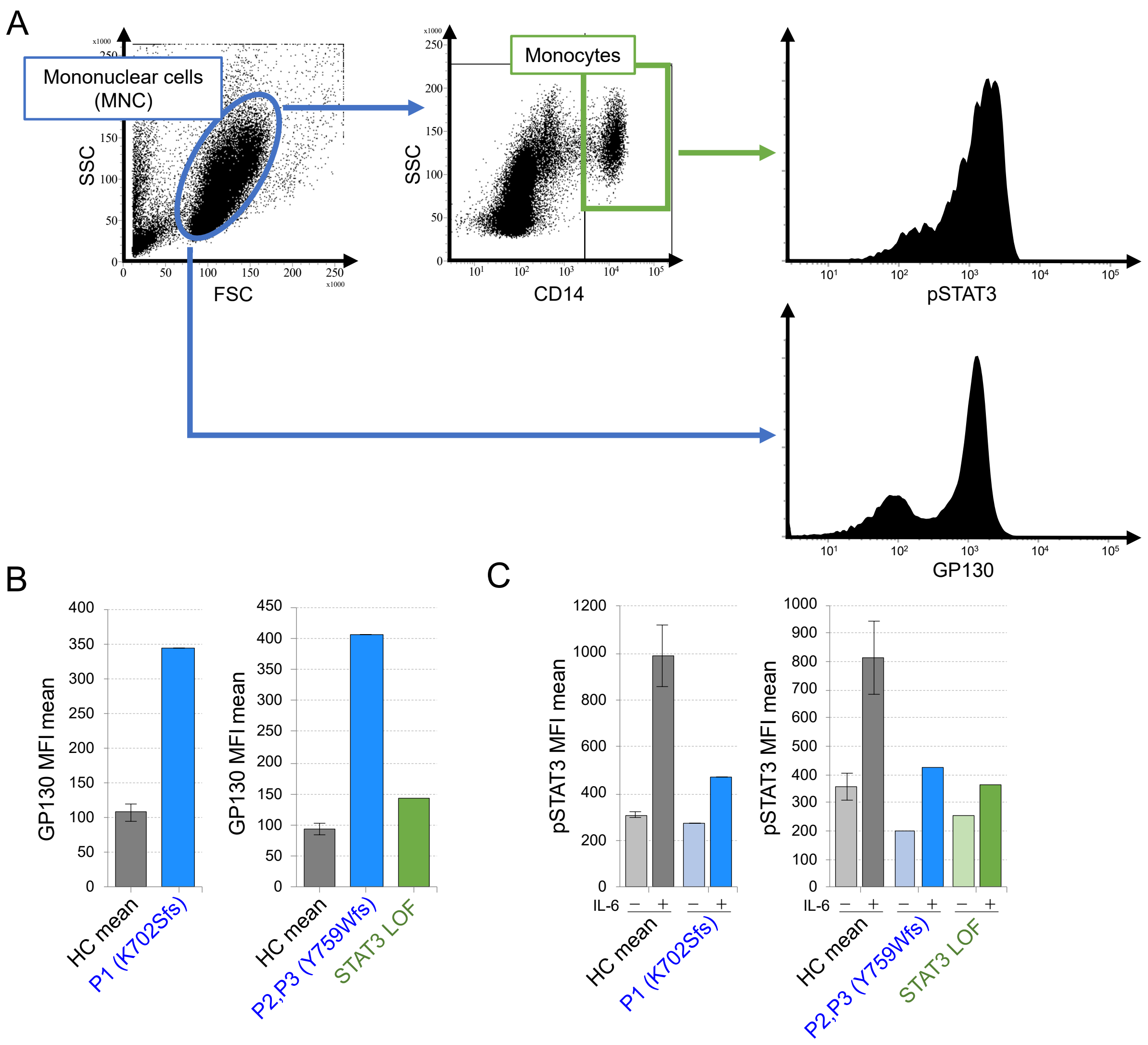

(A) Gating scheme of PBMCs. Mononuclear cells were gated based on forward and side scatter profiles (left panel). The MFI of GP130 was evaluated in mononuclear cells (bottom right panel). CD14-positive cells were gated within the mononuclear cells (monocytes, middle panel) and the MFI of pSTAT3 was evaluated (top right panel). **(B)** Quantitative MFI values for cell surface GP130 in PBMCs from patients and healthy subjects are shown; for HCs, values are the average of 3 samples, for *IL6ST* (Y759Wfs) patients, values are the average of P2 and P3. Each experiment was independently performed twice and a representative result is shown. **(C)** PBMCs from patients and healthy subjects were cultured and stimulated with IL-6 (50 ng/mL) or left unstimulated. The quantitative MFI values of pSTAT3 gated with CD14 after fixation and permeabilization of the collected cells are shown; for HCs, the values are the average of 3 samples, for *IL6ST* (Y759Wfs) patients, values are the average of P2 and P3. Each experiment was independently performed twice and a representative result is shown. HC; healthy control, MFI; mean fluorescence intensity, LOF; loss-of-function.

Figure S7. The mutants, which are predicted to be AD, are less active than the AR mutant and exhibit a DN effect.

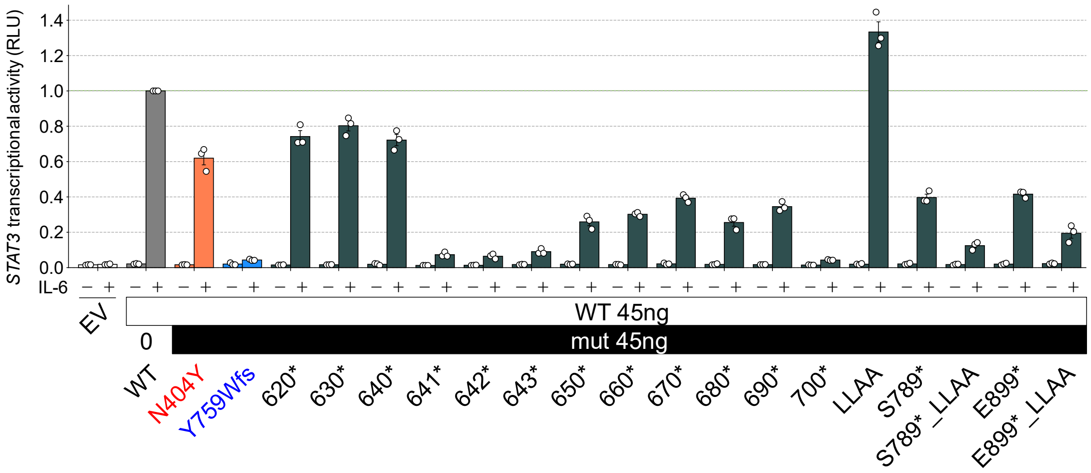

STAT3 transcriptional activity of GP130-KO-HEK293T cells transfected with either 45 ng of WT alone or with WT or its variants at a 1:1 ratio (WT:mut = 45 ng:45 ng) and stimulated with or without IL-6. RLU values were normalized to the poststimulation WT values as 1. Bars and error bars are the means and SEM of technical triplicates. This experiment was independently performed three times, and a representative result is shown. EV, empty vector; mut, mutant.

Figure S8. Functional analysis of nonsense or frameshift variants listed in gnomAD.

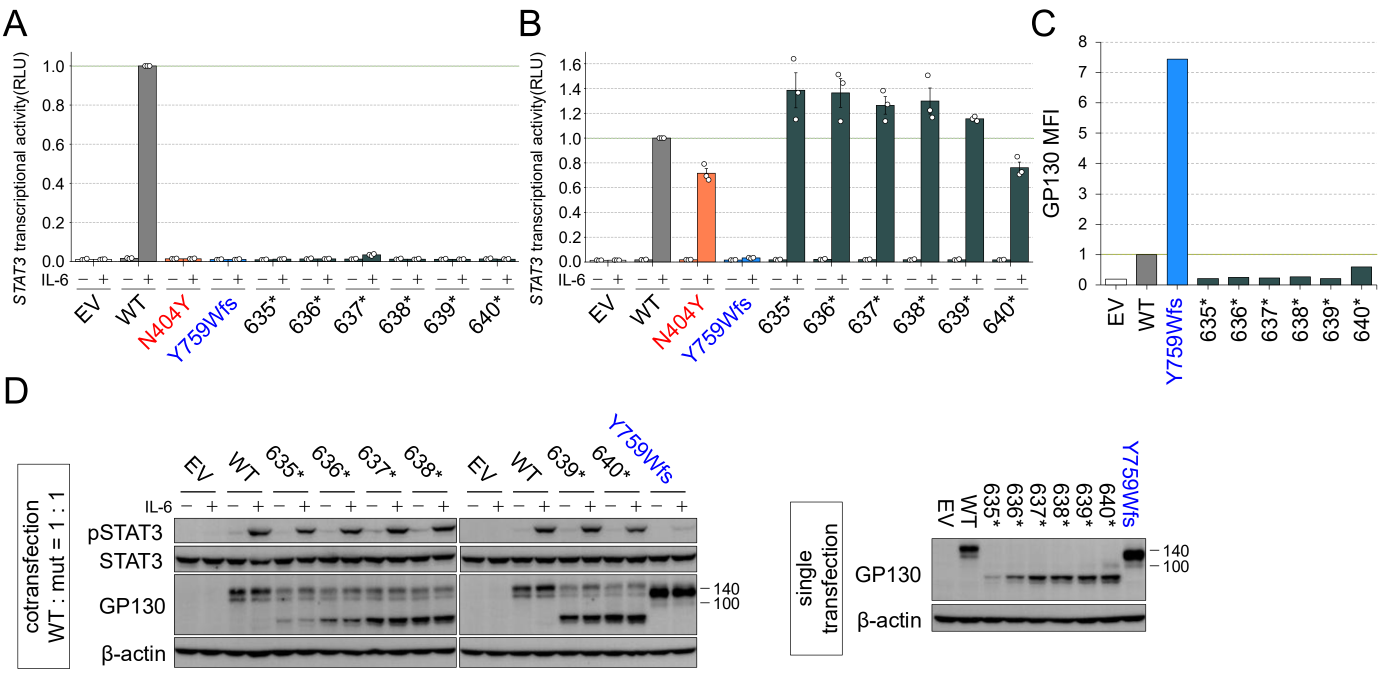

(A,B) STAT3 transcriptional activity in GP130-KO-HEK293T cells transfected with WT and/or variants and stimulated with or without IL-6 (**A**: single, **B**: 1:1 ratio). RLU values were normalized to the poststimulation WT value of 1. Bars and error bars are the means and SEM of technical triplicates. This experiment was independently performed three times, and a representative result is shown.
(C) MFI of cell surface GP130 expression in transfected GP130-KO-HEK293T cells. MFI values were normalized to the WT values as 1. This experiment was independently performed three times, and a representative result is shown. **(D)** Immunoblotting of pSTAT3, STAT3 and GP130 in GP130-KO-HEK293T cells transfected with WT and/or variants and stimulated with or without IL-6 (above: cotransfection at a 1:1 ratio, below: single). β-Actin was used as a loading control. This experiment was independently performed twice, and a representative result is shown. EV, empty vector; mut, mutant.

Figure S9. Functional analysis of nonsense or frameshift variants listed in gnomAD.

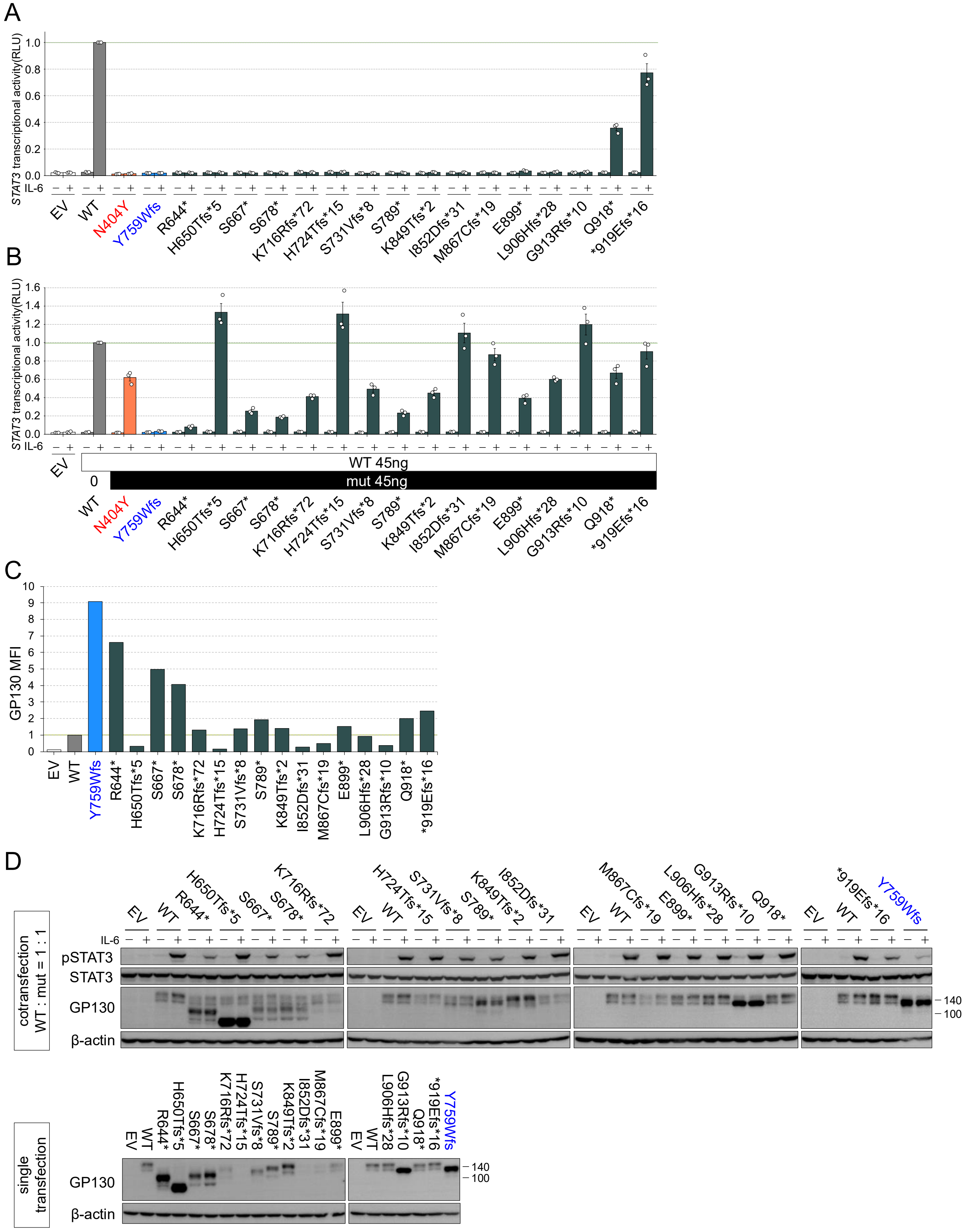

(A,B) STAT3 transcriptional activity in GP130-KO-HEK293T cells transfected with WT and/or variants and stimulated with or without IL-6 (**A**: single, **B**: 1:1 ratio). RLU values were normalized to the poststimulation WT value of 1. Bars and error bars are the means and SEM of technical triplicates. This experiment was independently performed three times, and a representative result is shown.
(C) MFI of cell surface GP130 expression in transfected GP130-KO-HEK293T cells. MFI values were normalized to the WT values as 1. This experiment was independently performed three times, and a representative result is shown. **(D)** Immunoblotting of pSTAT3, STAT3 and GP130 in GP130-KO-HEK293T cells transfected with WT and/or variants and stimulated with or without IL-6 (above: cotransfection at a 1:1 ratio, below: single). β-Actin was used as a loading control. This experiment was independently performed twice, and a representative result is shown. EV, empty vector; mut, mutant.

Table S1. Deep immunophenotyping of patients with the AD-*IL6ST* mutation K702Sfs.

Patient<br>Variant allele	Family 1<br>P1<br>K702Sfs*7<br>H855P		Reference Value (20)
B cells (% of lymphocytes)	0.4	low	12.2±4.4
Memory B cells (% of CD19+)	12	low	18.5±8.2
T cells (% of lymphocytes)	83.1	high	67.8±5.4
CD4+ T cells (% of CD3+)	60.1	normal	59.9±9.9
Naïve T cells (% of CD3+CD4+)	85.7	high	47.2±9.3
Memory T cells (% of CD3+CD4+)	11.9	low	-
Th1 cells (% of CD3+CD4+CD45RO+)	4.94	low	22.6±8.7
Th2 cells (% of CD3+CD4+CD45RO+)	71.9	high	35.3±13.8
Th17 cells (% of CD3+CD4+CD45RO+)	21.4	normal	23.7±4.3
CD8+ T cells (% of CD3+)[%]	38.4	normal	34.1±8.7
CD8+ Naïve T cells (% of CD3+CD8+)	96.6	high	64.2±7.1
CD8+ Memory T cells (% of CD3+CD8+)	2.5	low	-

Table S2. The nonsense or frameshift variants within the intracellular domain registered in gnomAD and the UK Biobank.

variants		Allele Frequency		Reference
amino acid	codon	gnomAD	UK Biobank	
p.Arg644Ter	c.1930C>T	7.13E-07	4.26E-06	
p.His650ThrfsTer5	c.1947dup	6.91E-07	N.D	
p.Ser667Ter	c.2000C>A	6.89E-07	N.D	
p.Ser678Ter	c.2033C>A	1.20E-06	1.06E-06	
p.Lys716ArgfsTer72	c.2147del	1.59E-06	N.D	
p.His724ThrfsTer15	c.2168dup	6.84E-07	N.D	
p.Ser731ValfsTer8	c.2190dup	6.58E-06	N.D	Arlabosse et al. (16)
p.Ser789Ter	c.2366C>G	6.84E-07	N.D	
p.Lys849ThrfsTer2	c.2542_2545dup	6.84E-07	N.D	
p.Ile852AspfsTer31	c.2552dup	6.84E-07	N.D	
p.Met867CysfsTer19	c.2599del	1.20E-06	1.06E-06	
p.Glu899Ter	c.2694dup	6.57E-06	N.D	
p.Leu906HisfsTer28	c.2716_2719del	6.84E-07	N.D	
p.Gly913ArgfsTer10	c.2736dup	1.59E-06	N.D	
p.Gln918Ter	c.2752C>T	2.40E-06	4.26E-06	
p.Ter919GlufsTer16	c.2755del	1.20E-06	N.D	

N.D, no data.

Table S3. The antibodies used in the panel of deep immunophenotyping.

Color	marker	Vender	Catalog	Clone
BUV395	CD45RA	BD Biosciences	740315	5H9
Ghost Dye UV450	Live/dead	Tonbo Biosciences	13-0868-T500	
BUV496	CD16	BD Biosciences	612944	3G8
BUV563	CCR5	BD Biosciences	741401	2D7/CCR5
BUV615	CD314(NKG2D)	BD Biosciences	751232	1D11
BUV661	CD39	BD Biosciences	749967	TU66
BUV737	CD56	BD Biosciences	612766	NCAM16.2
BUV805	CD8	BD Biosciences	612889	SK1
BV421	CCR7	BioLegend	353208	G043H7
SuperBright436	CD123	Thermo Fisher Scientific	62-1239-42	6H6
eFluor 450	CD11c	Thermo Fisher Scientific	48-0116-42	3.9
BV510	CD3	BioLegend	344828	SK7
cFluor V547	CD20	Cytek Biosciences	R7-20111	2H7
BV570	IgM	BioLegend	314518	MHM-88
BV605	CCR4	BioLegend	359418	L291H4
BV650	CD28	BioLegend	302946	CD28.2
BV711	CCR6	BioLegend	353436	G034E3
BV750	CXCR5	BD Biosciences	747111	RF8B2
BV785	PD-1	BioLegend	329930	EH12.2H7
cFluor B515	cD141	Cytek Biosciences	R7-20113	M80
cFluor B532	CD57	Cytek Biosciences	RC-00127	HNK-1
cFluor B548	CD14	Cytek Biosciences	R7-20115	63D3
PerCP	CD45	Tonbo Biosciences	67-9459-T500	2D1
PerCP-Cy5.5	CD2	BioLegend	309226	TS1/8
PerCP-Vio700	TCRgd	Miltenyi Biotec	130-113-506	11F2
cFluor BYG750	IgD	Cytek Biosciences	RC-00521	IgD26
PE	NKG2C(CD159C)	Miltenyi Biotec	130119776	REA205
cFluor YG584	CD4	Cytek Biosciences	R7-20041	SK3
PE-Dazzle594	CD337(NKp30)(NCR3)	BioLegend	325232	p30-15
cFluor YG610	CD24	Cytek Biosciences	R7-20659	SN3
PE-Cy5	FAS	BioLegend	305610	DX2
PE-Fire 700	CD25	BioLegend	356146	M-A251
PE-Cy7	CXCR3	BioLegend	353720	G025H7
PE-Fire810	HLA-DR	BioLegend	307683	L243
APC	NKG2A	Miltenyi Biotec	130113563	REA110
cFluor R668	CD1c	Cytek Biosciences	R7-20119	L161
Spark NIR685	CD19	BioLegend	302270	HIB19
cFluor R720	CD127	Cytek Biosciences	RC-00009	A019D5
APC H7	CD27	BD Biosciences	560222	M-T271
APC-Fire 810	CD38	BioLegend	356644	HIT2
