# Supplementary material for: Fine mapping of heterozygous IL6ST nonsense variants underlying autosomal dominant hyper-IgE syndrome
Source: JCI Insight. 2025 Jun 17;10(14):e190065. doi: 10.1172/jci.insight.190065 (PMC12288962; doi:10.1172/jci.insight.190065)

Full unedited blot for Figure 4E_single_pSTAT3

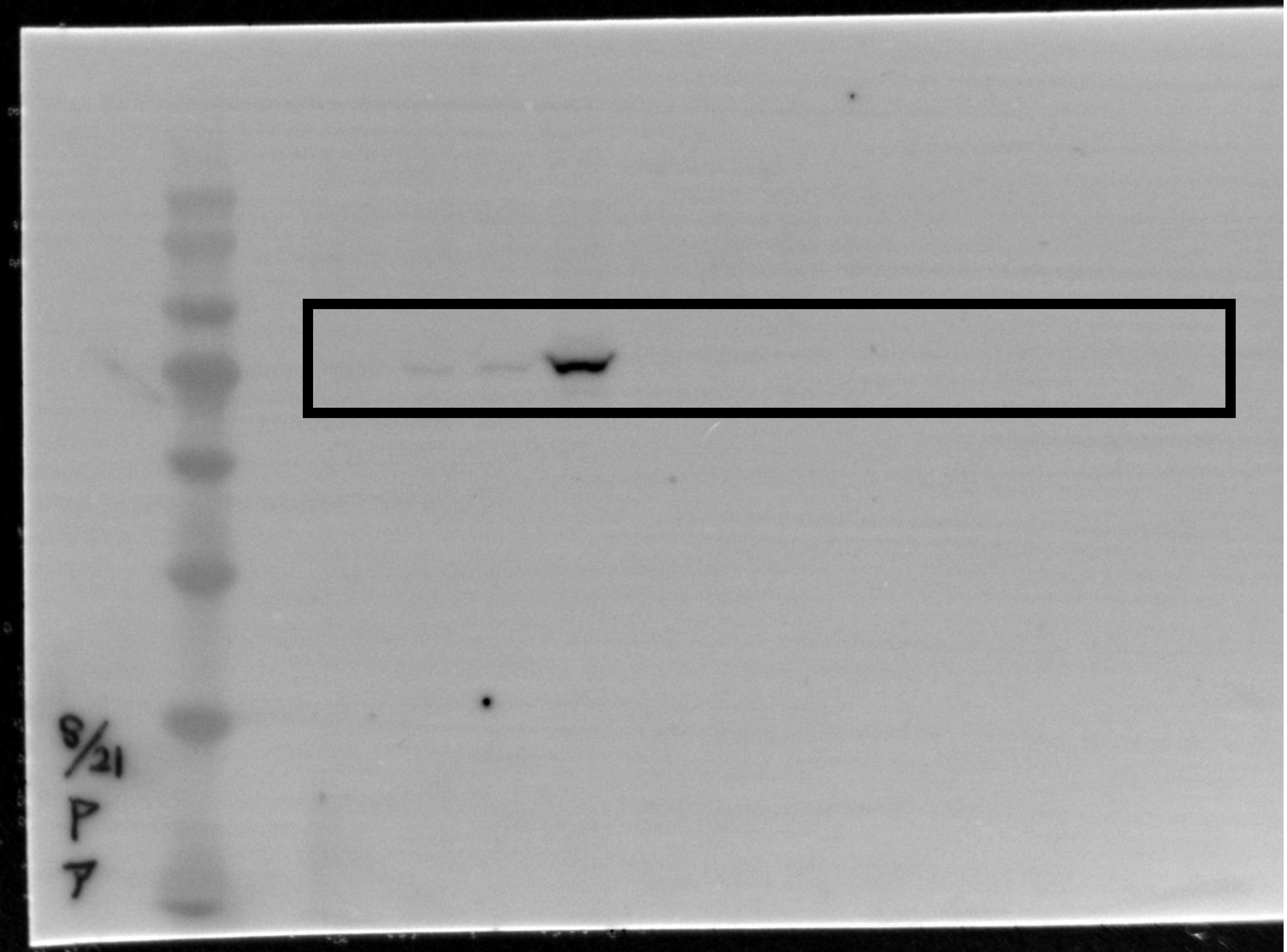

Full unedited blot for Figure 4E_single_STAT3

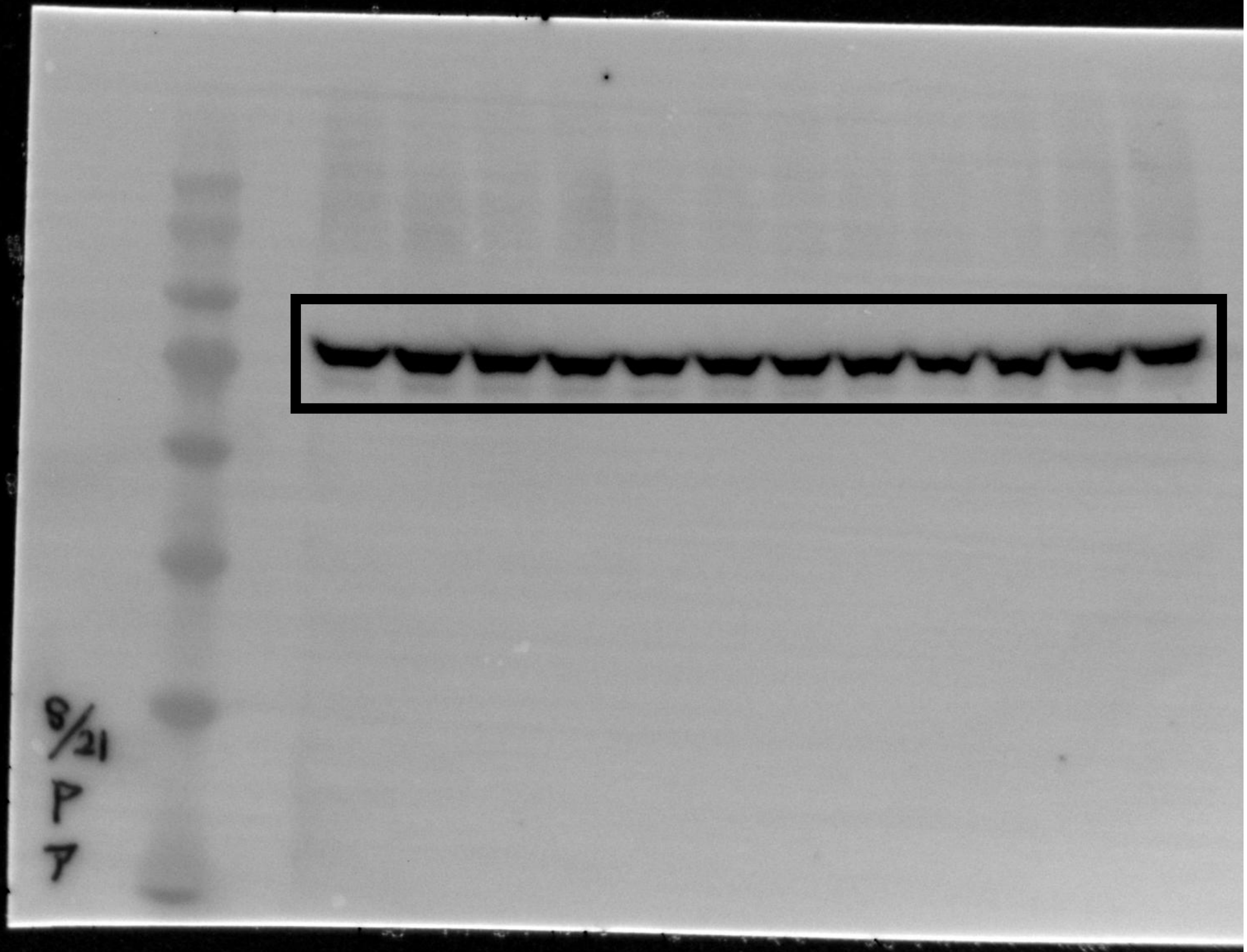

Full unedited blot for Figure 4E_single_GP130

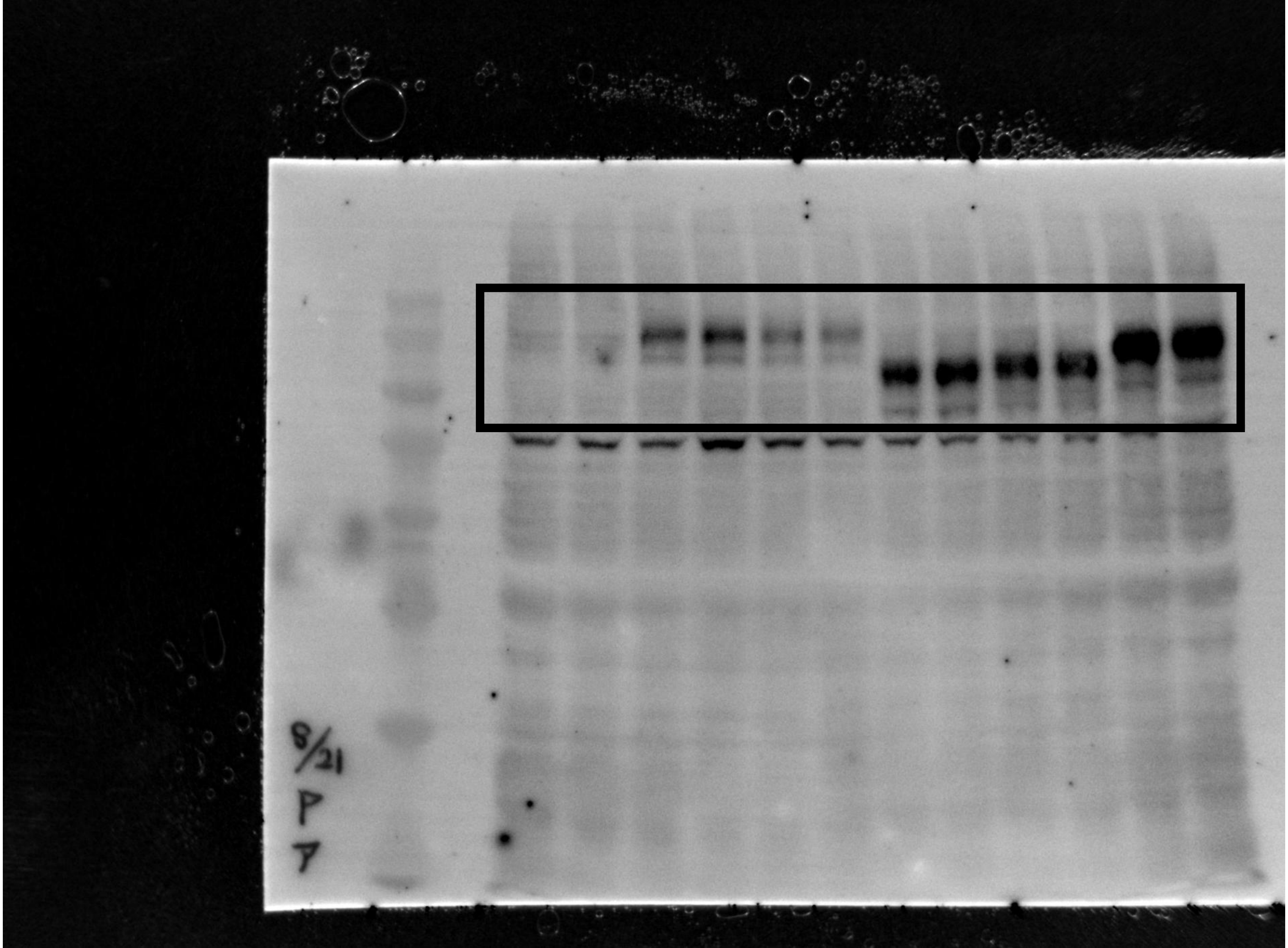

Full unedited blot for Figure 4E_single_β-actin

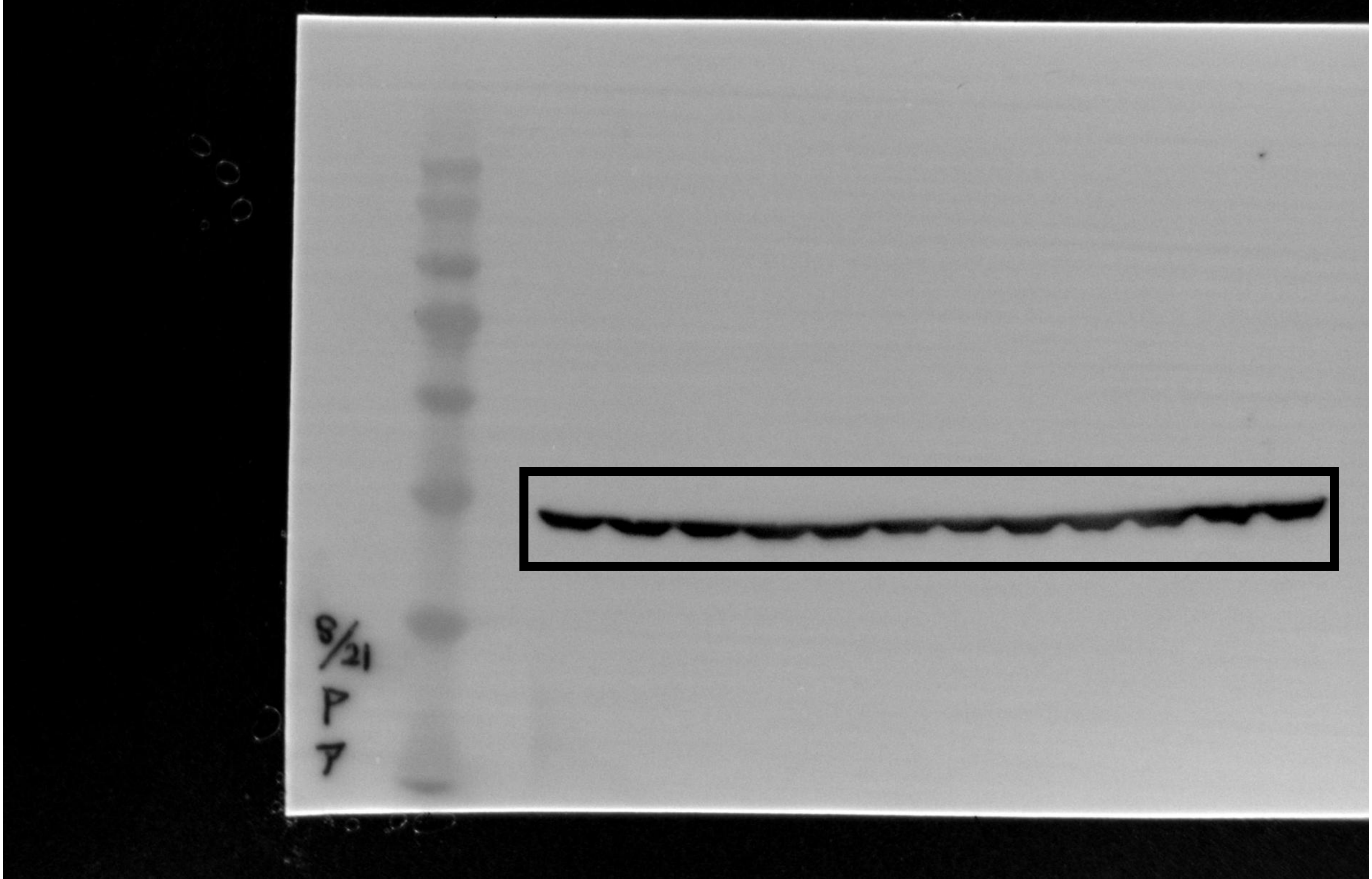

Full unedited blot for Figure 4E_cotransfection_pSTAT3

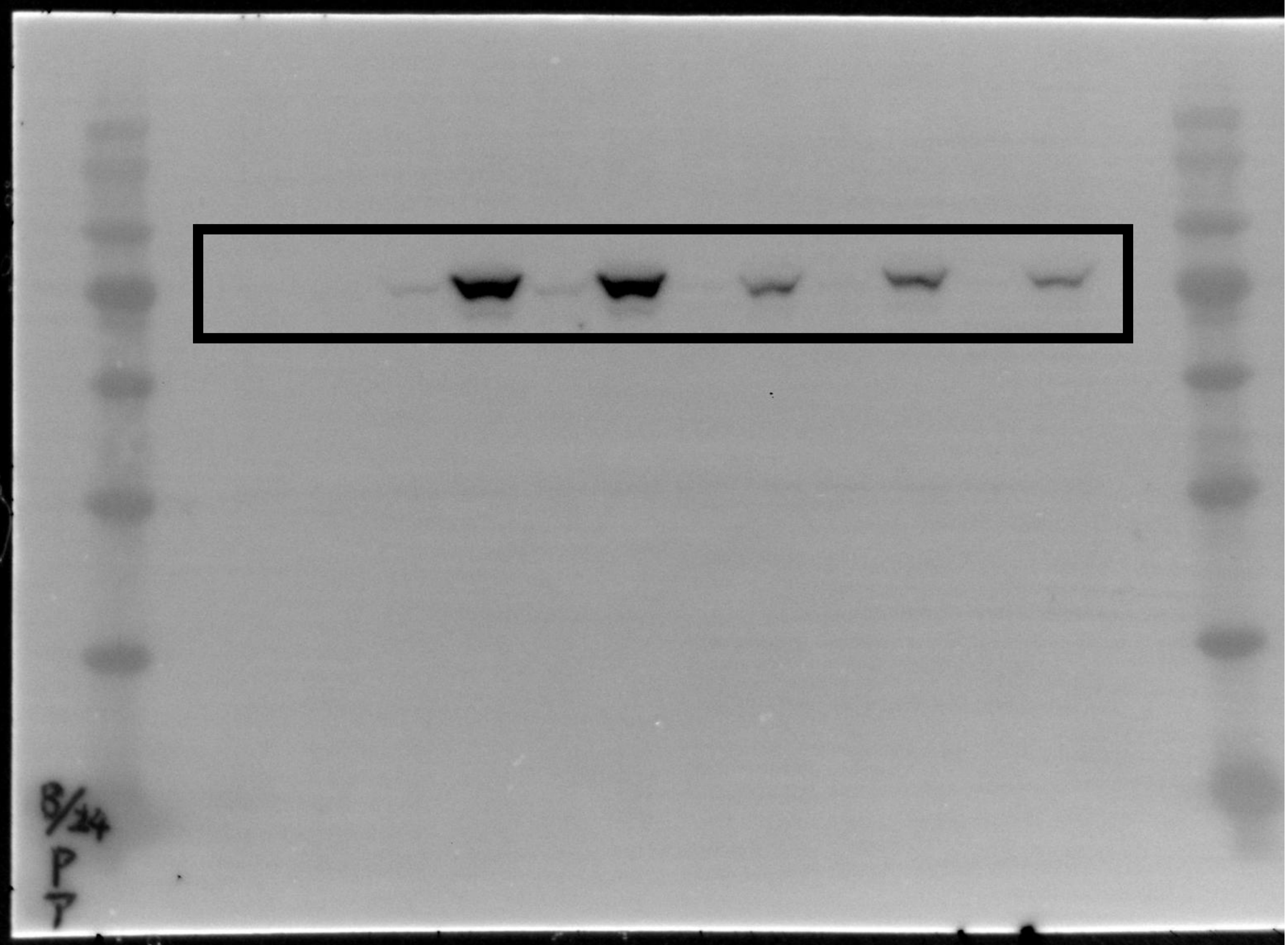

Full unedited blot for Figure 4E_cotransfection_STAT3

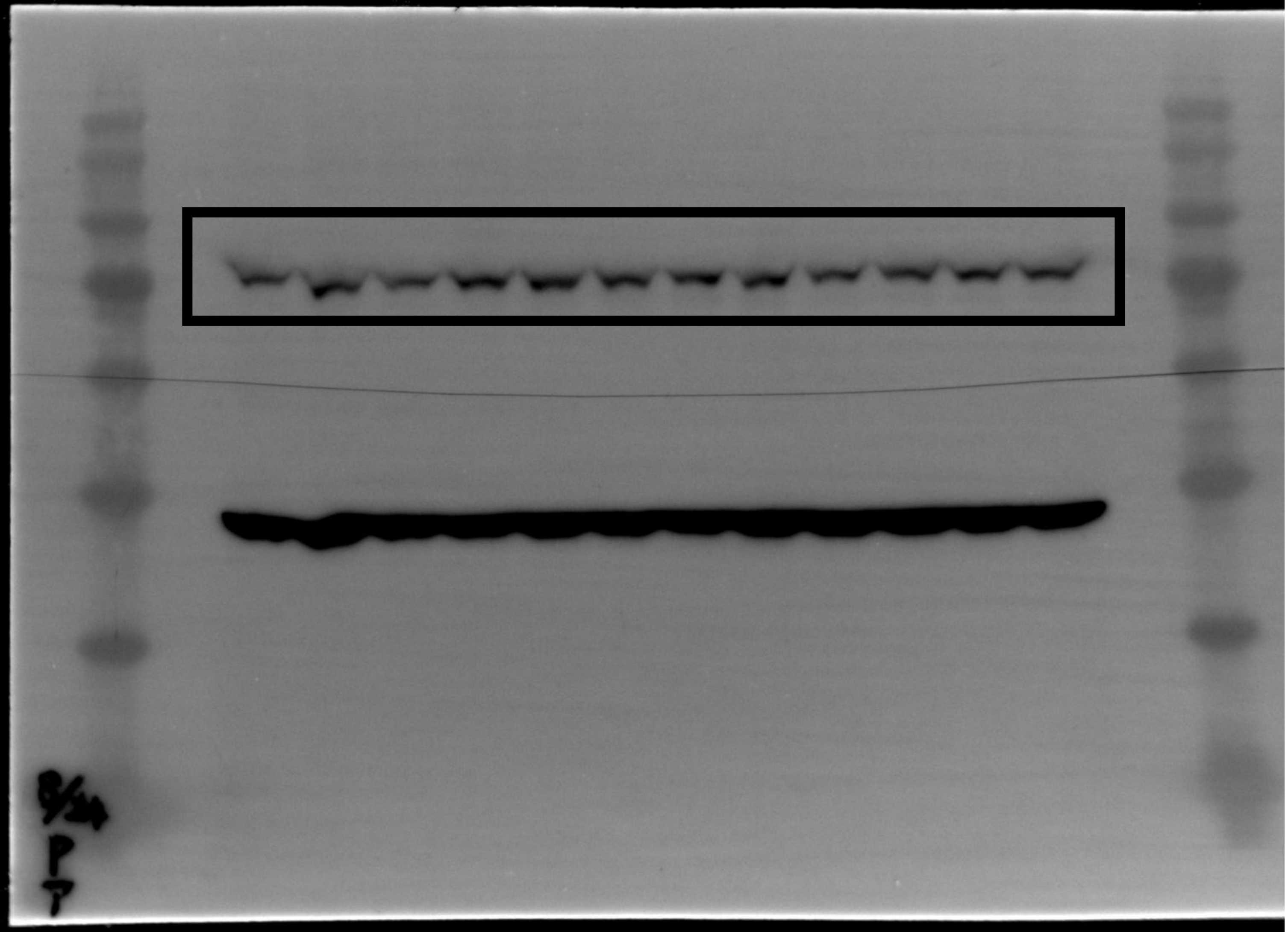

Full unedited blot for Figure 4E_cotransfection_GP130

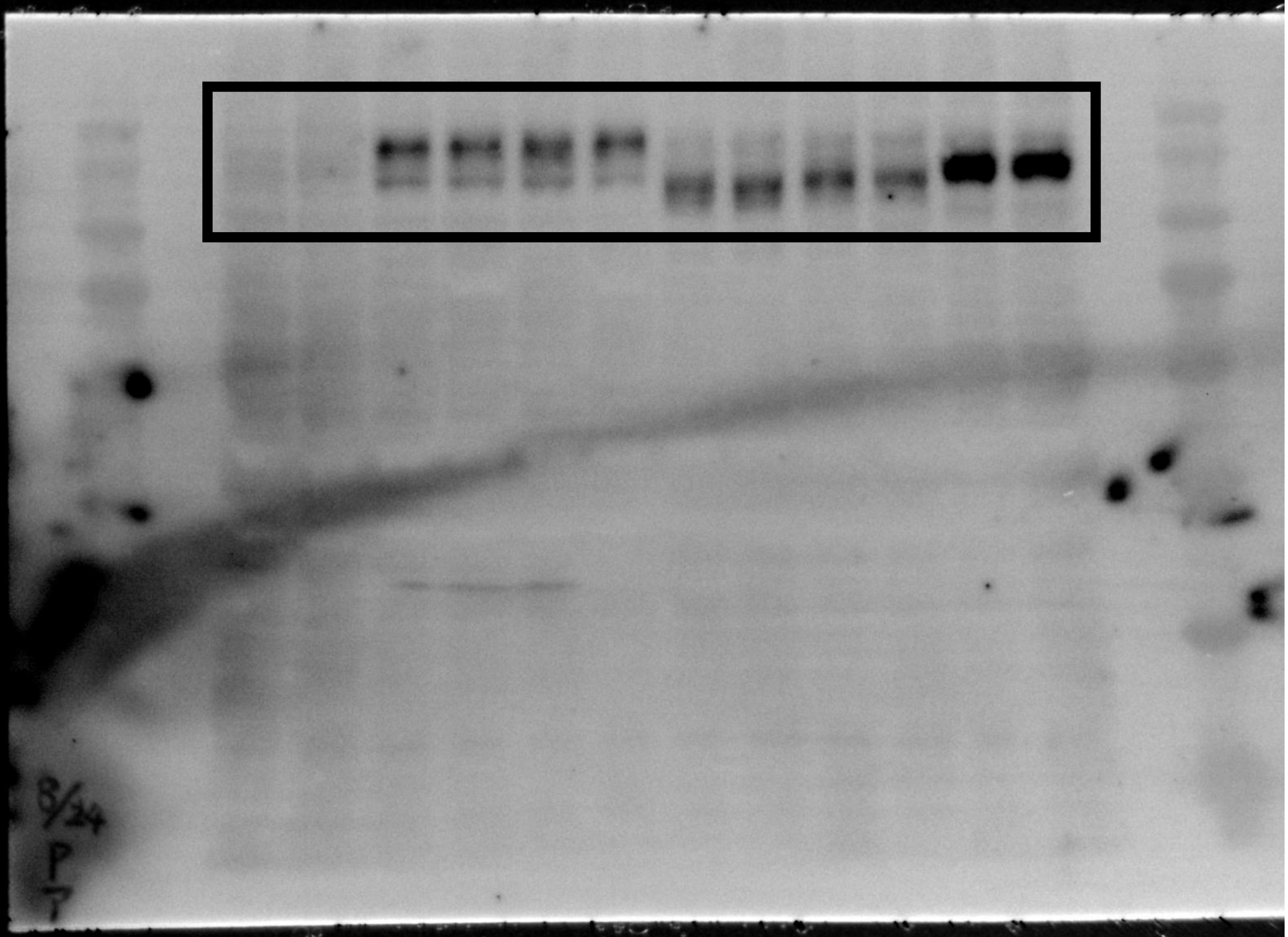

Full unedited blot for Figure 4E_cotransfection_β-actin

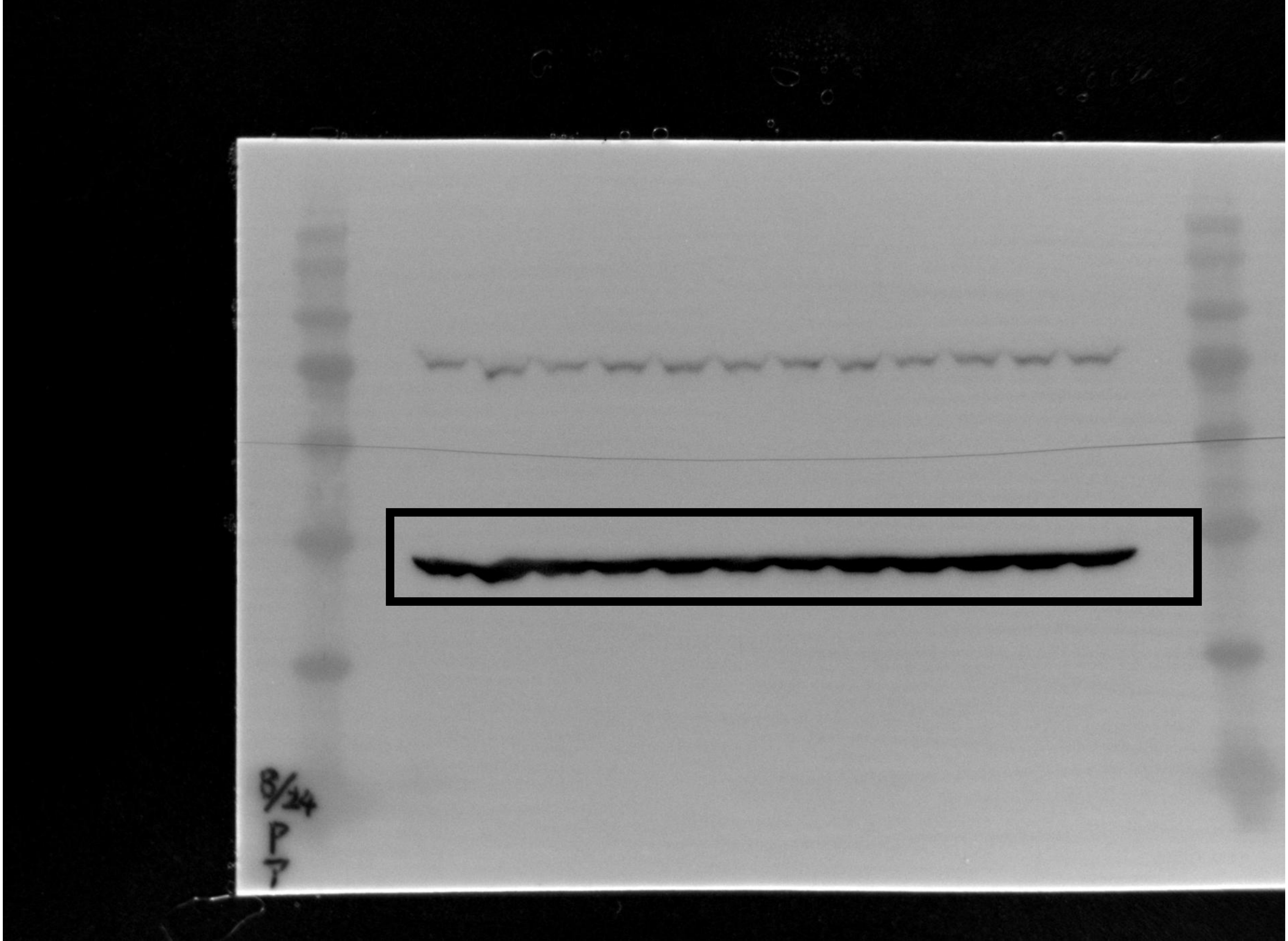

Full unedited blot for Figure 6D_620-660_pSTAT3

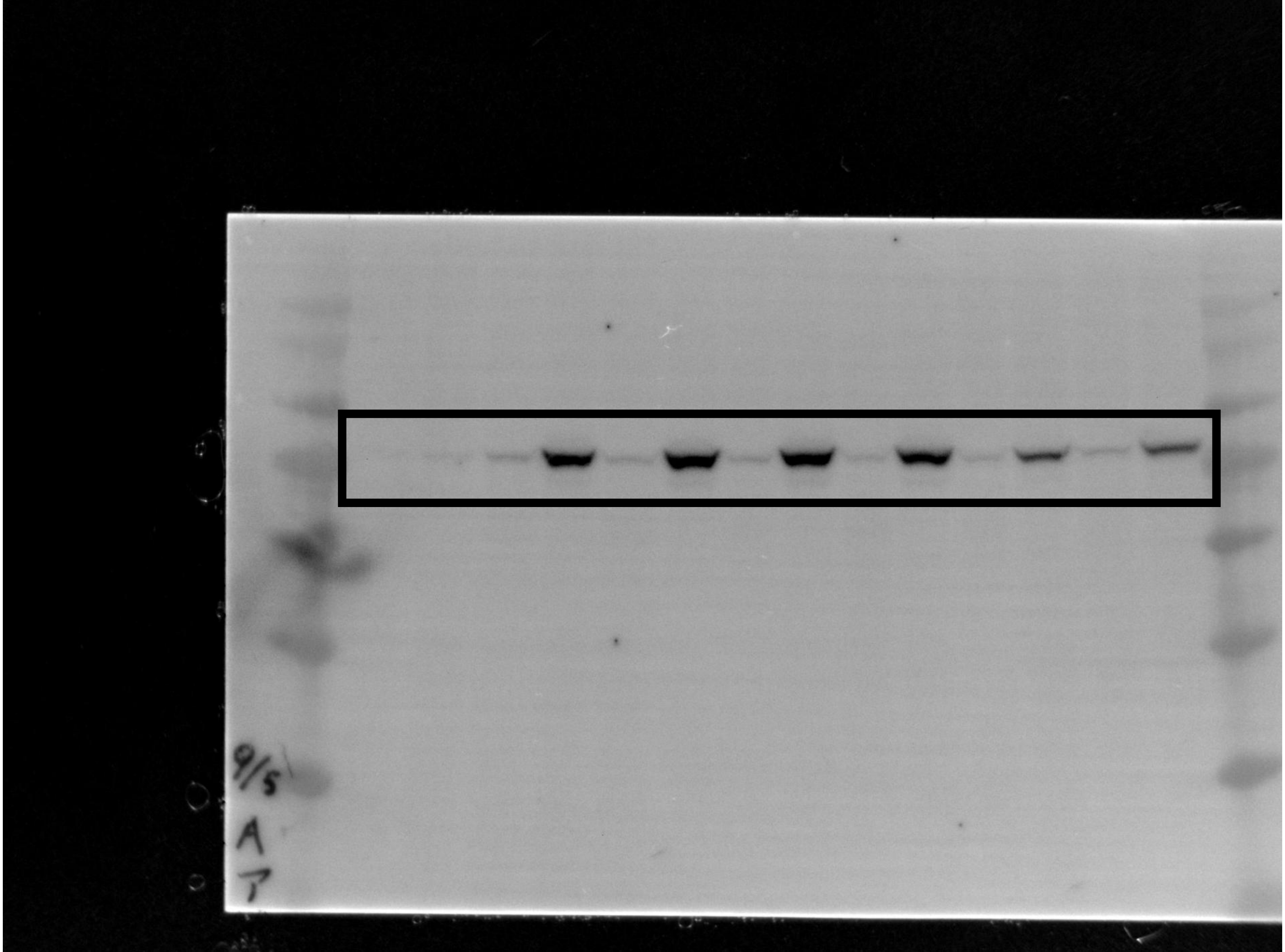

Full unedited blot for Figure 6D_620-660_STAT3

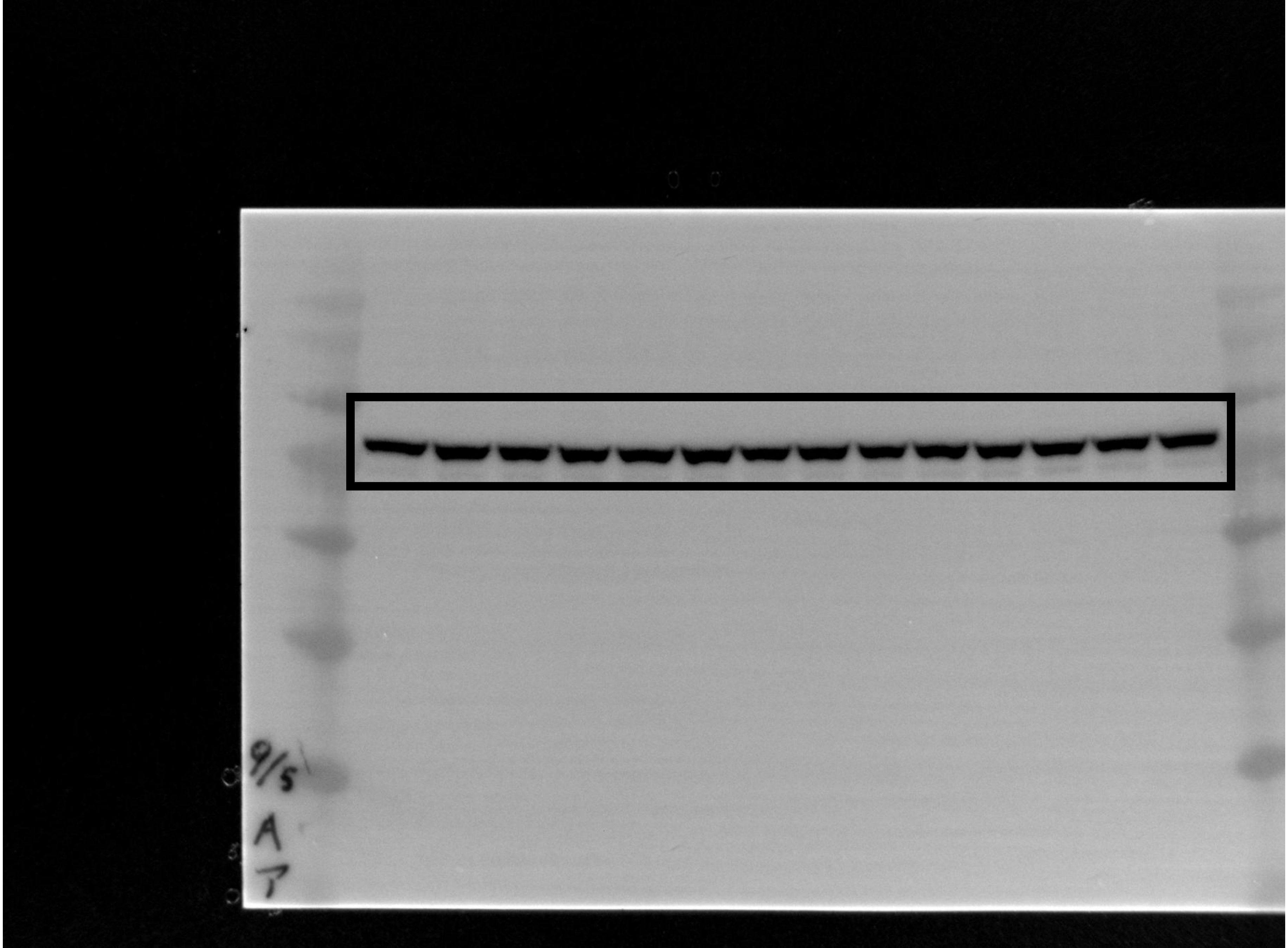

Full unedited blot for Figure 6D_620-660_GP130

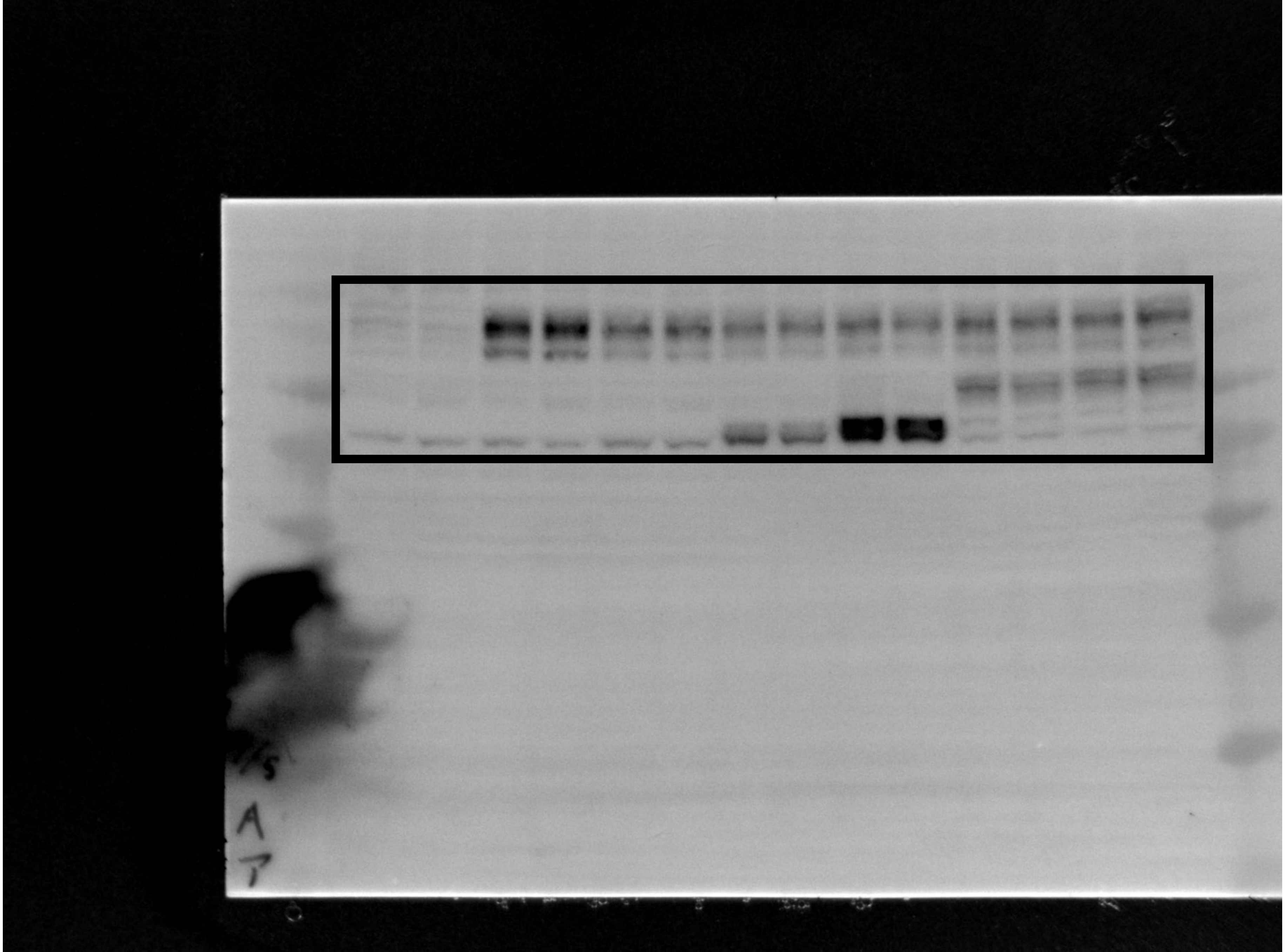

Full unedited blot for Figure 6D_620-660_β-actin

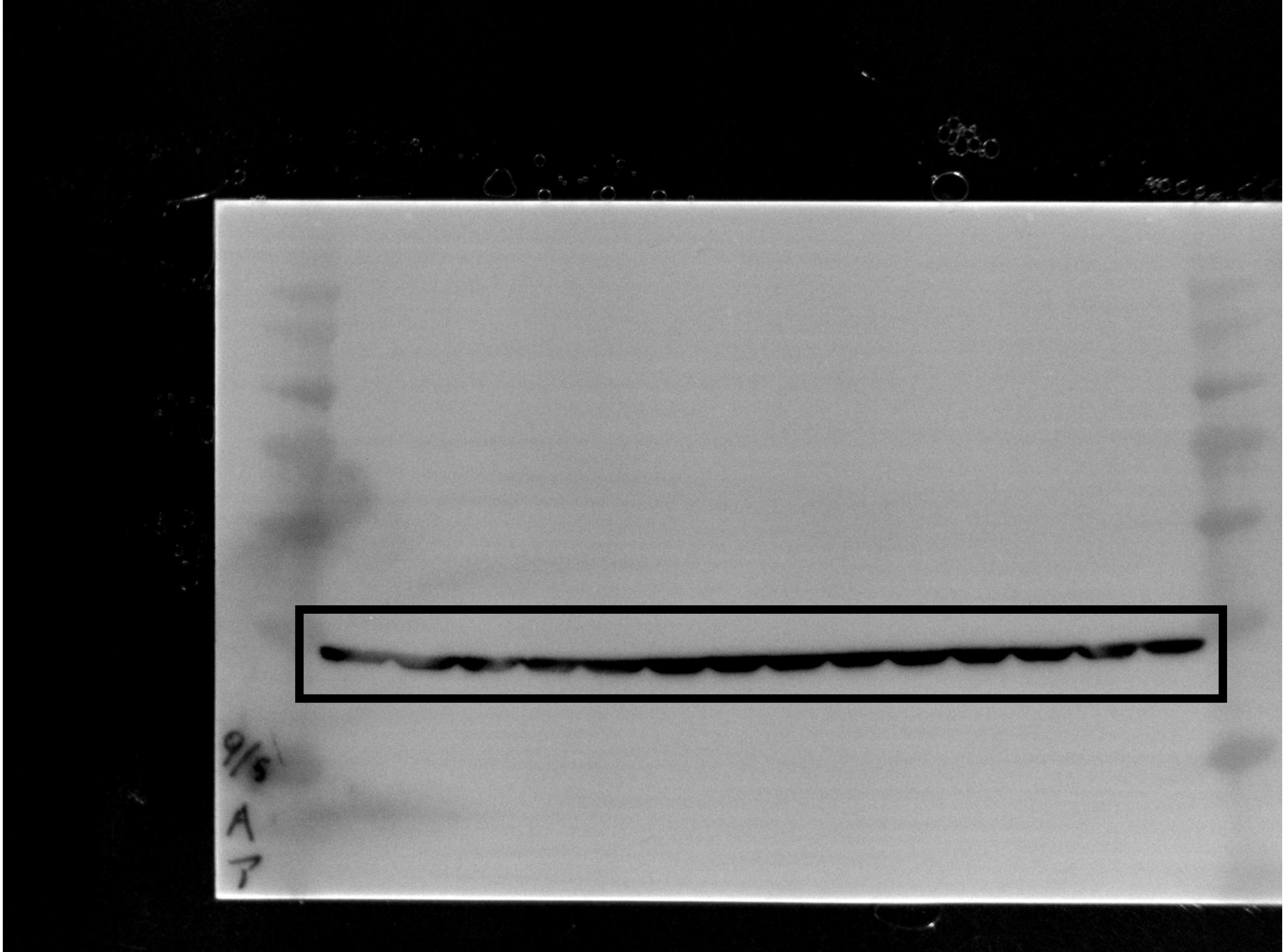

Full unedited blot for Figure 6D_670-700_pSTAT3

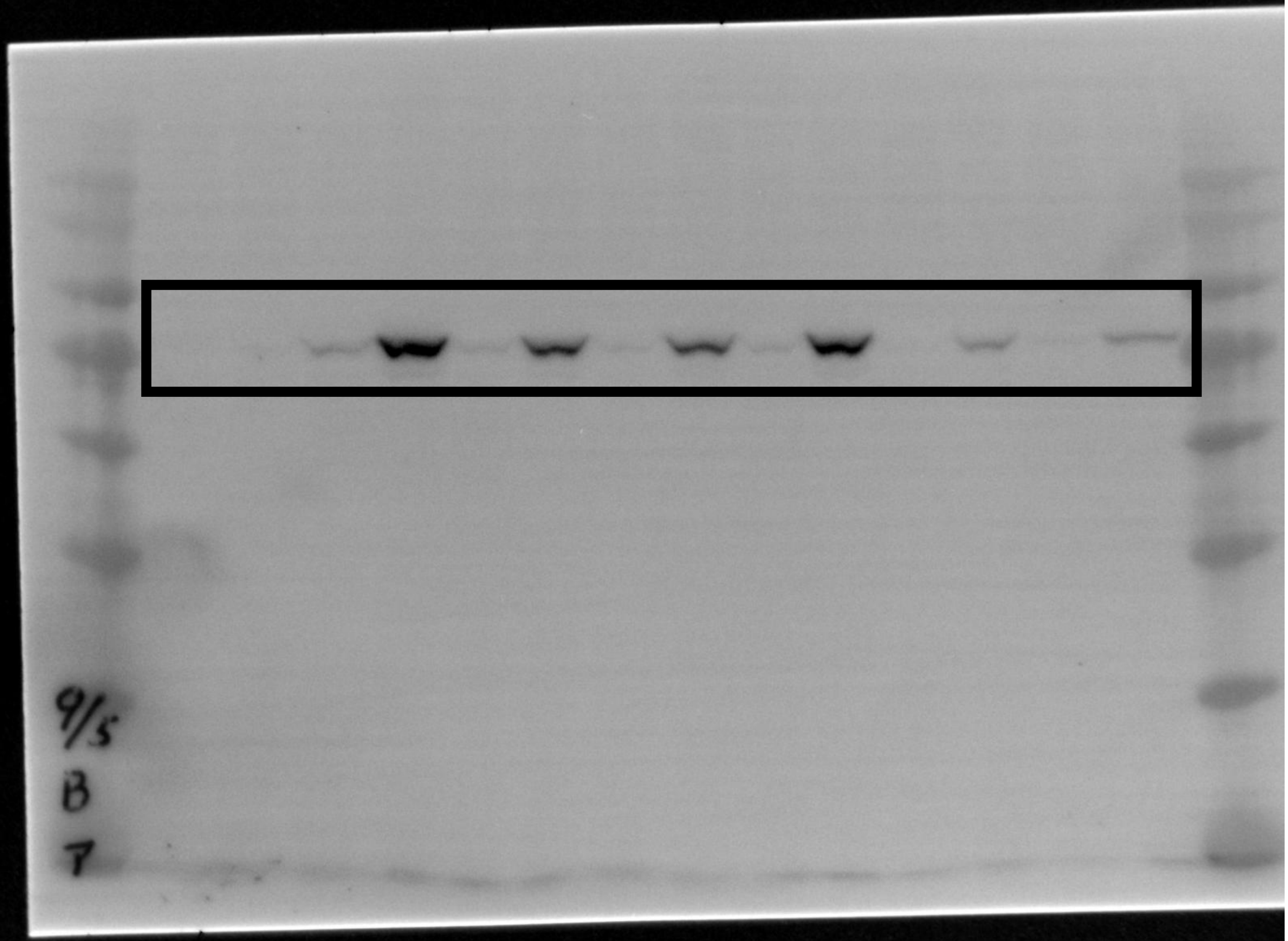

Full unedited blot for Figure 6D_670-700_STAT3

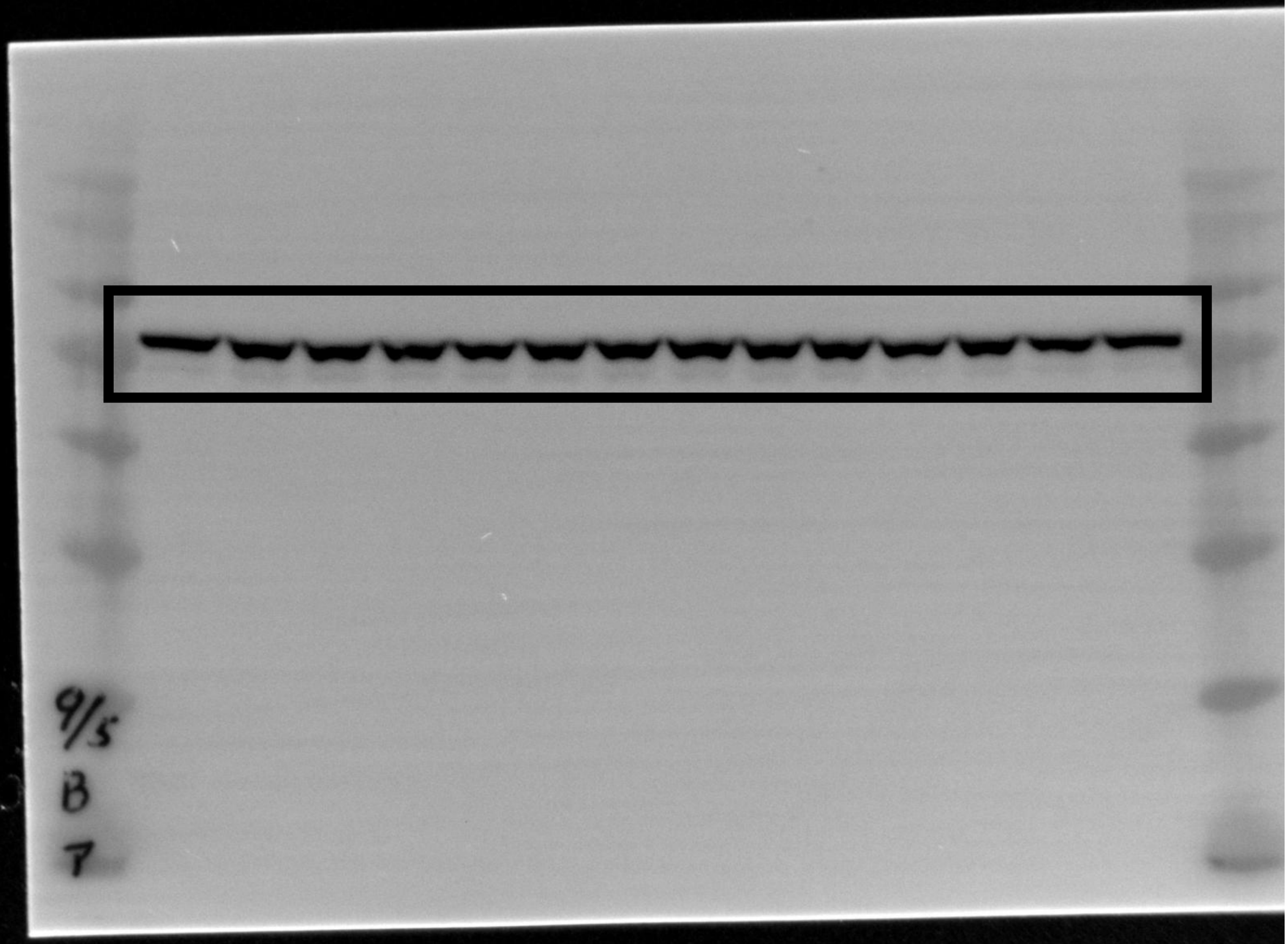

Full unedited blot for Figure 6D_670-700_GP130

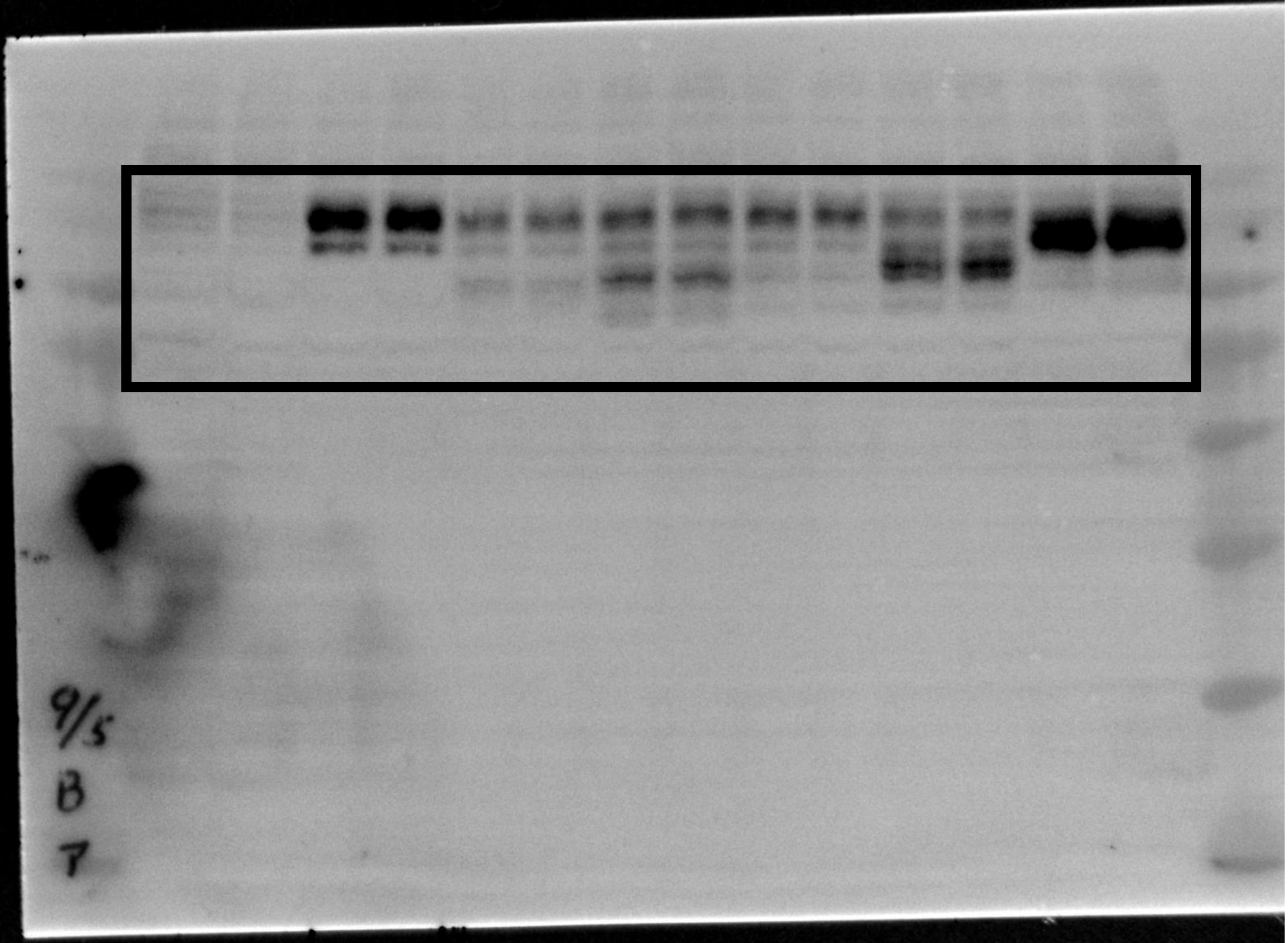

Full unedited blot for Figure 6D_670-700_β-actin

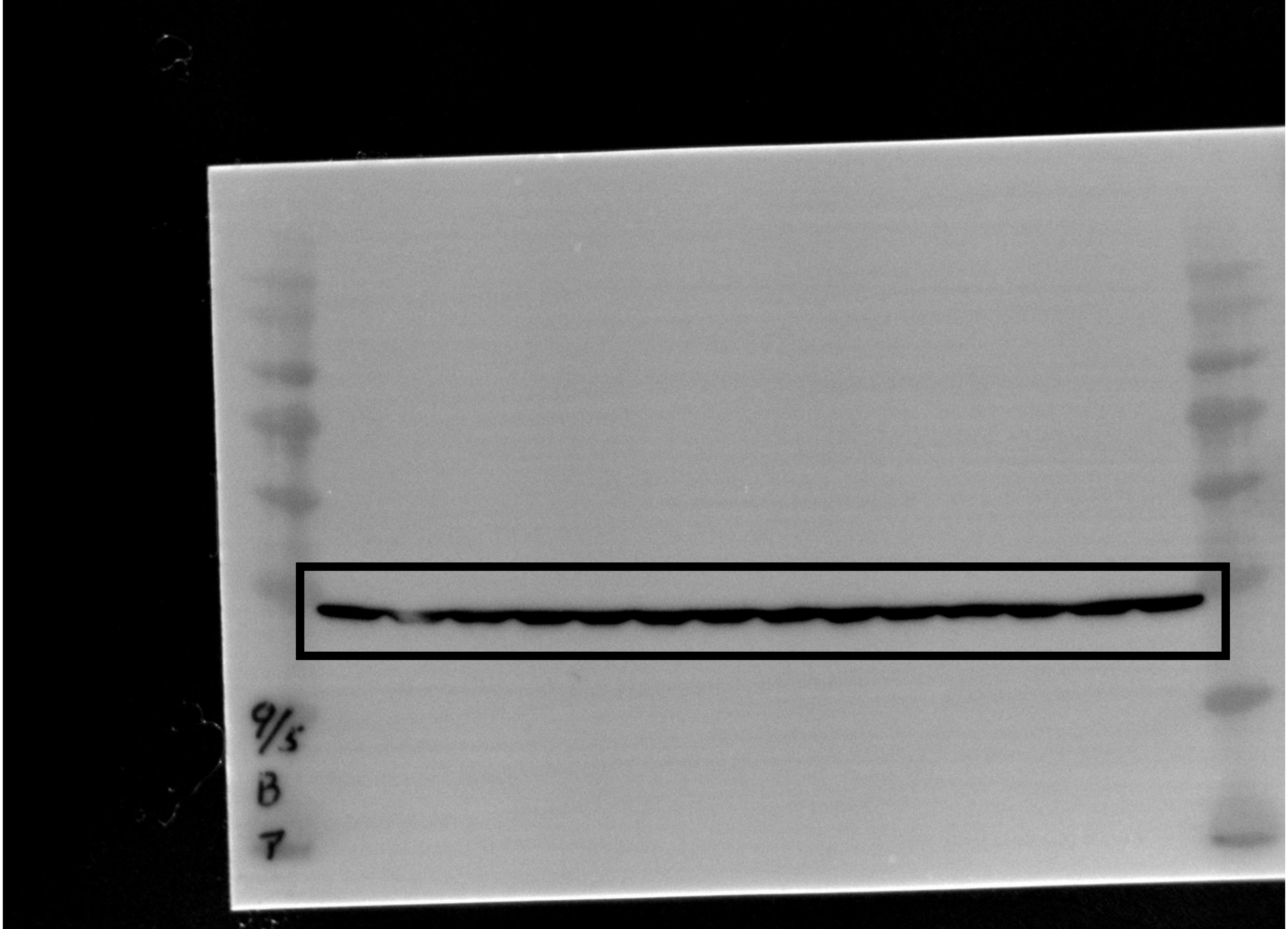

Full unedited blot for Figure 6D_641-643_pSTAT3

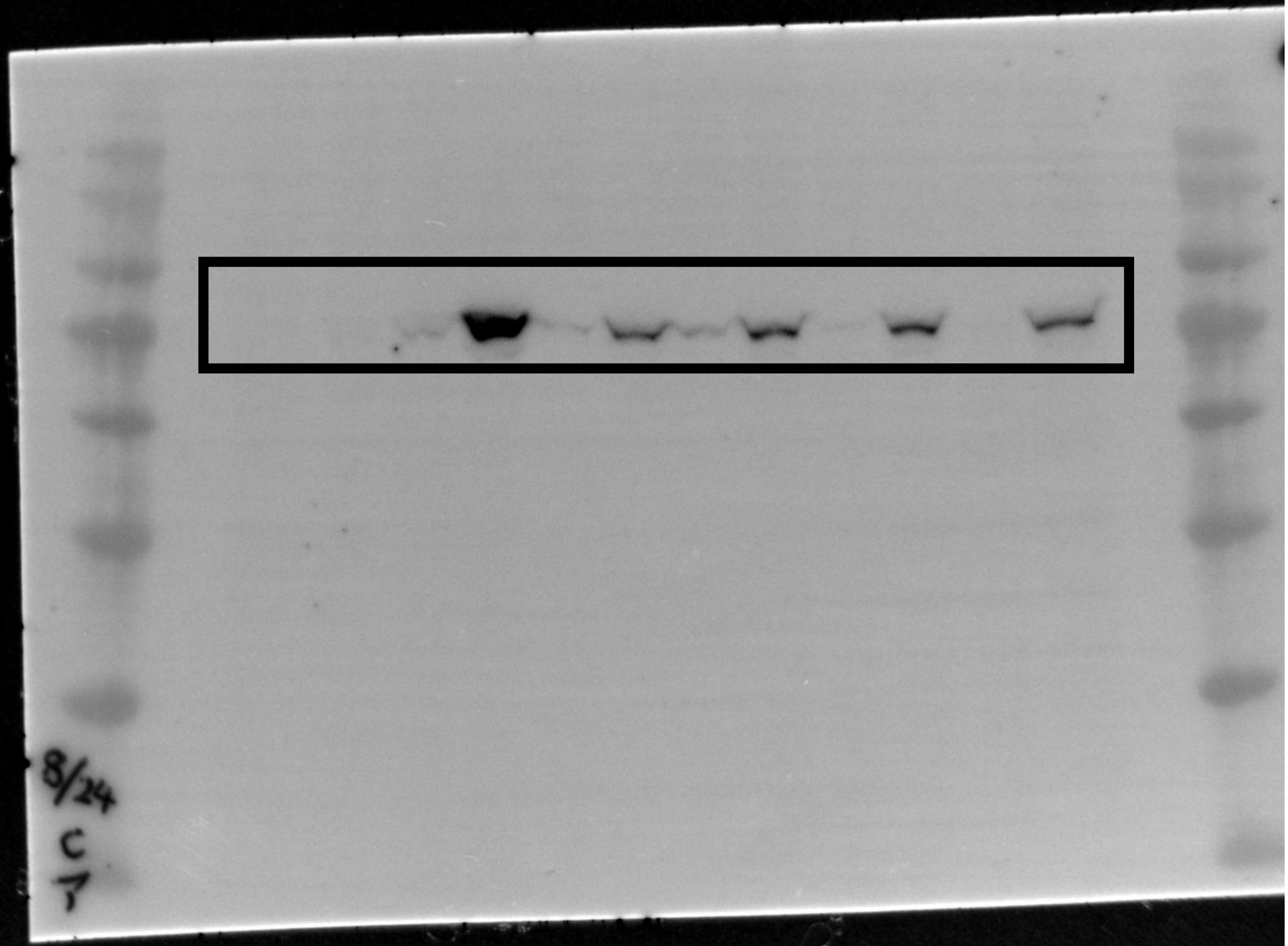

Full unedited blot for Figure 6D_641-643_STAT3

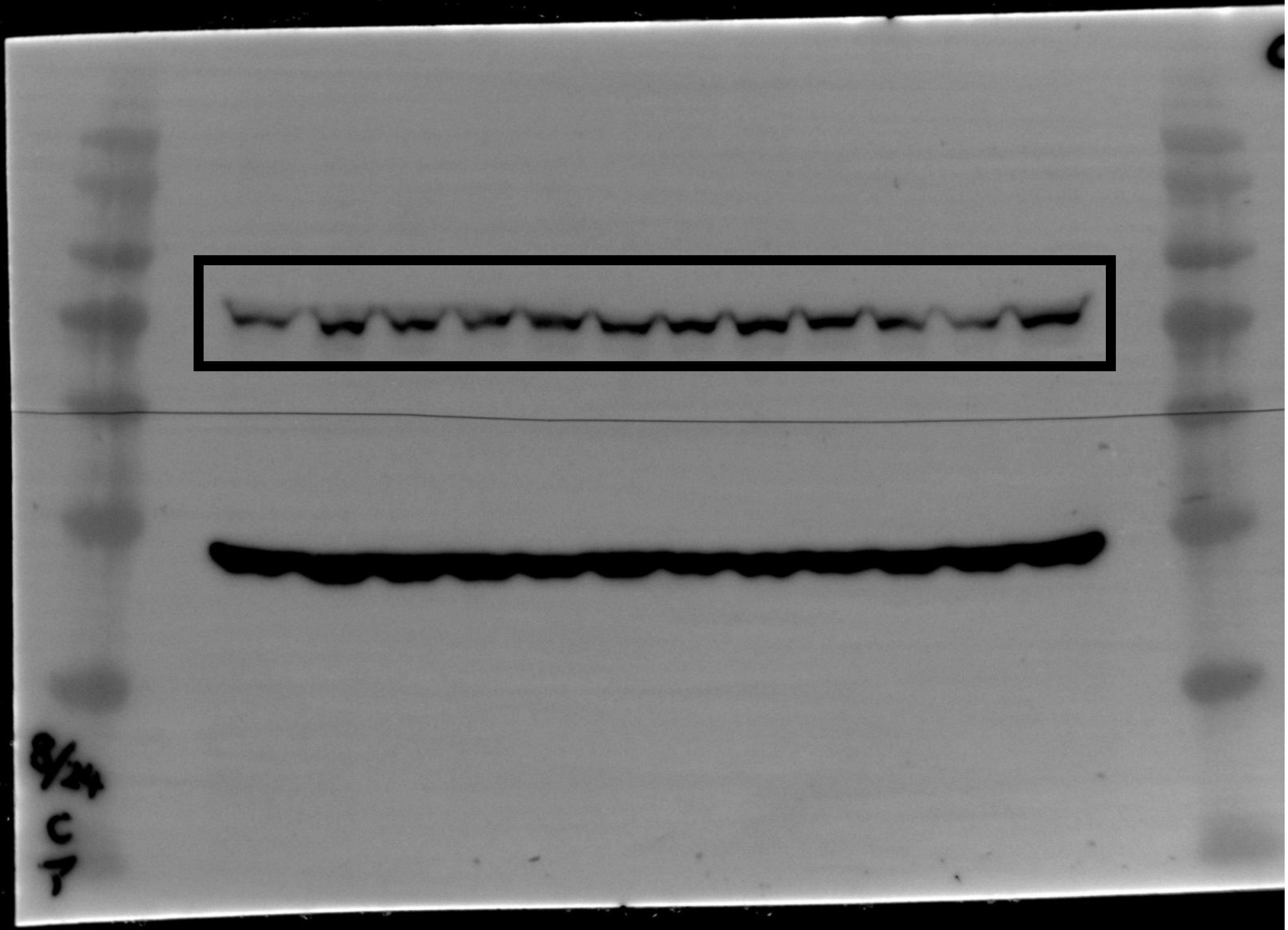

Full unedited blot for Figure 6D_641-643_GP130

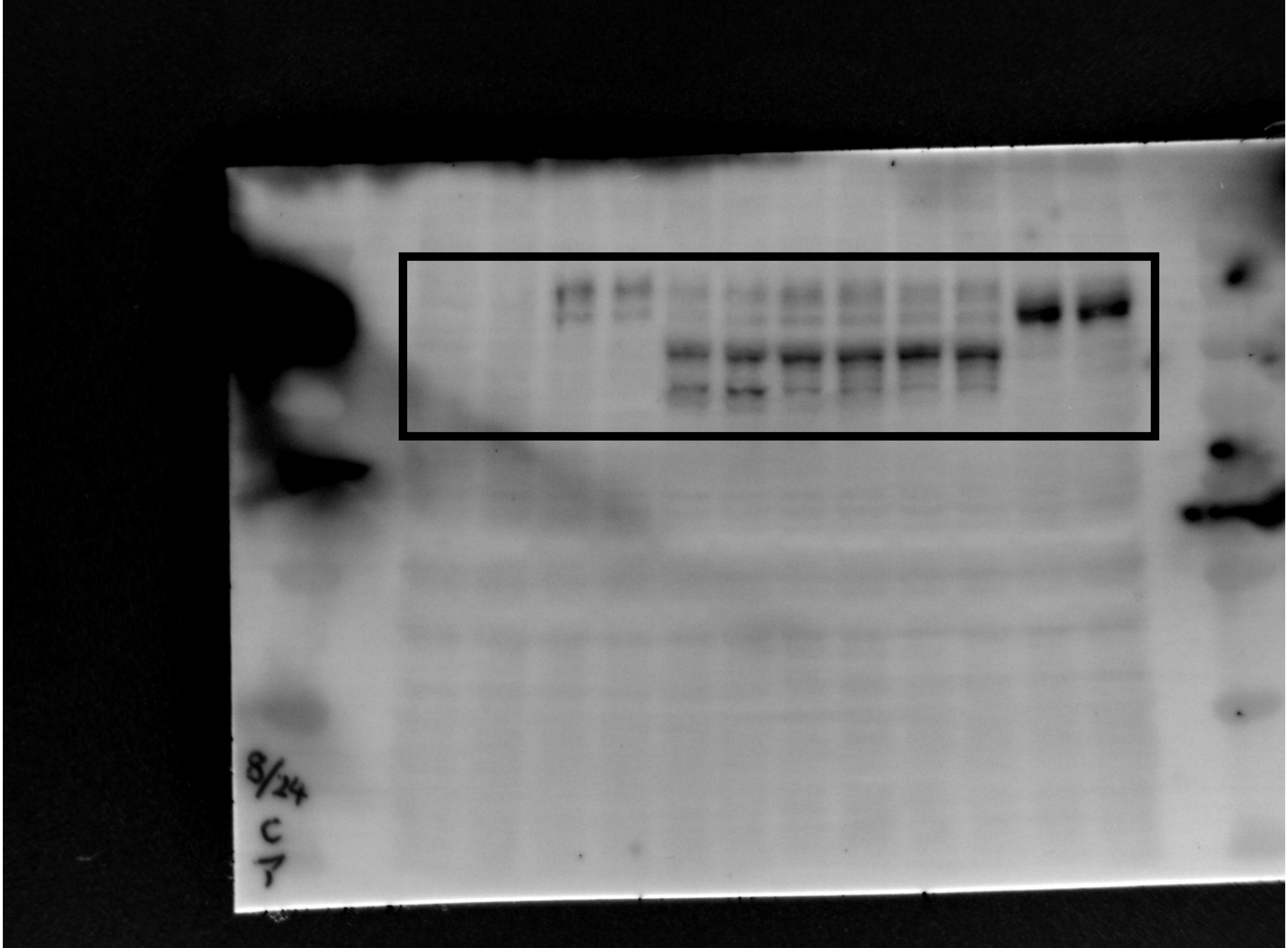

Full unedited blot for Figure 6D_641-643_β-actin

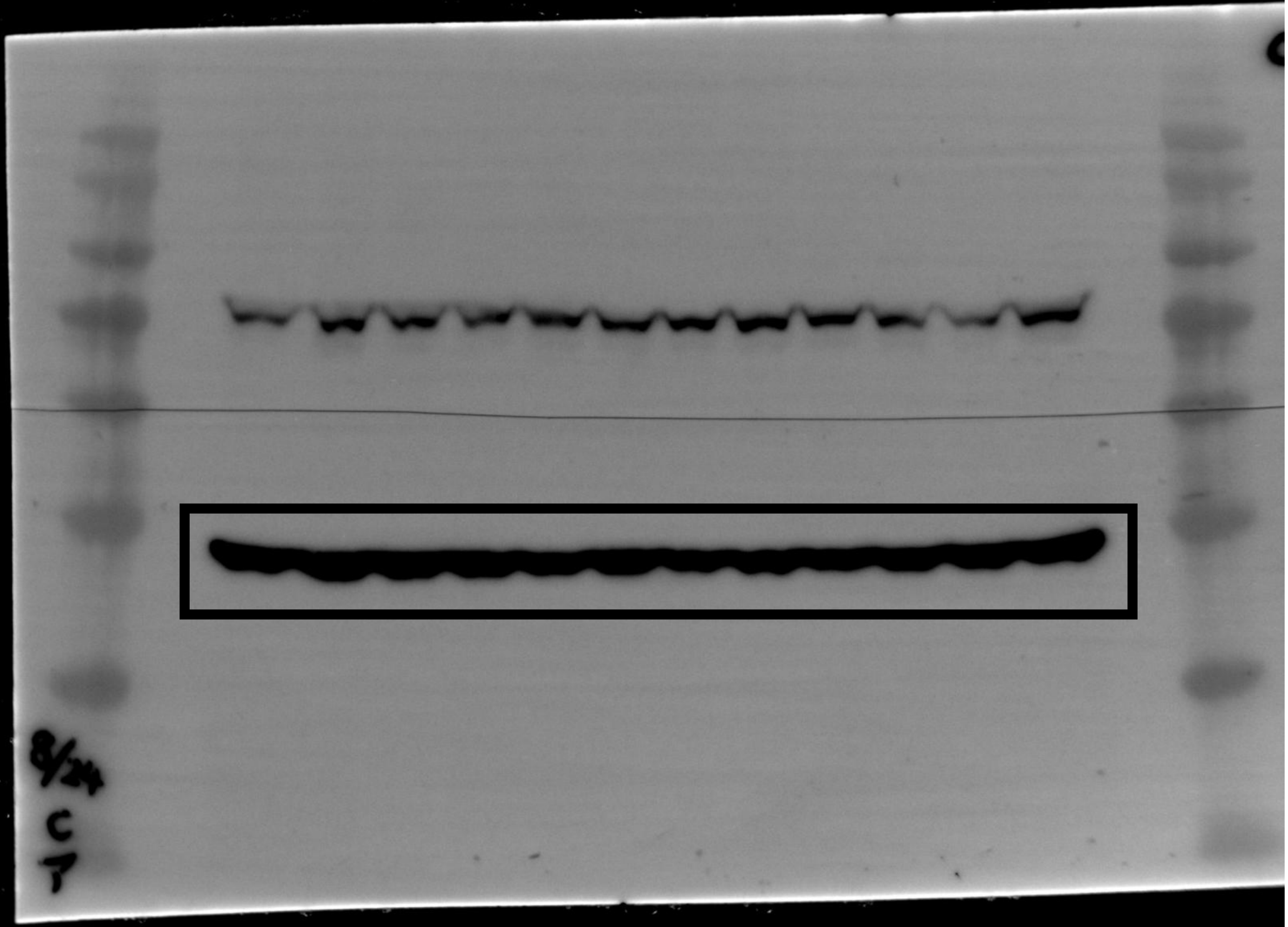

Full unedited blot for Figure 7F_1_pSTAT3

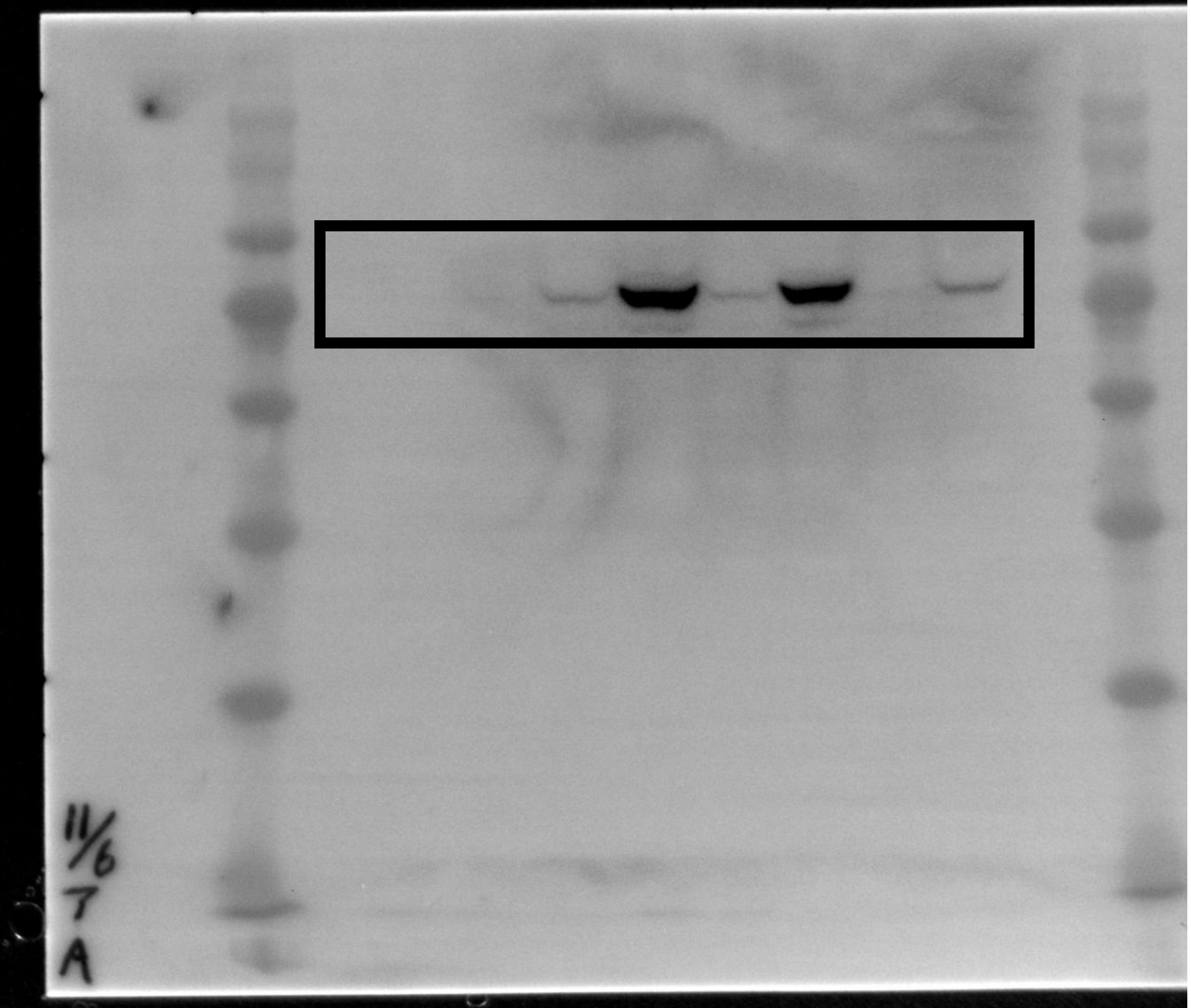

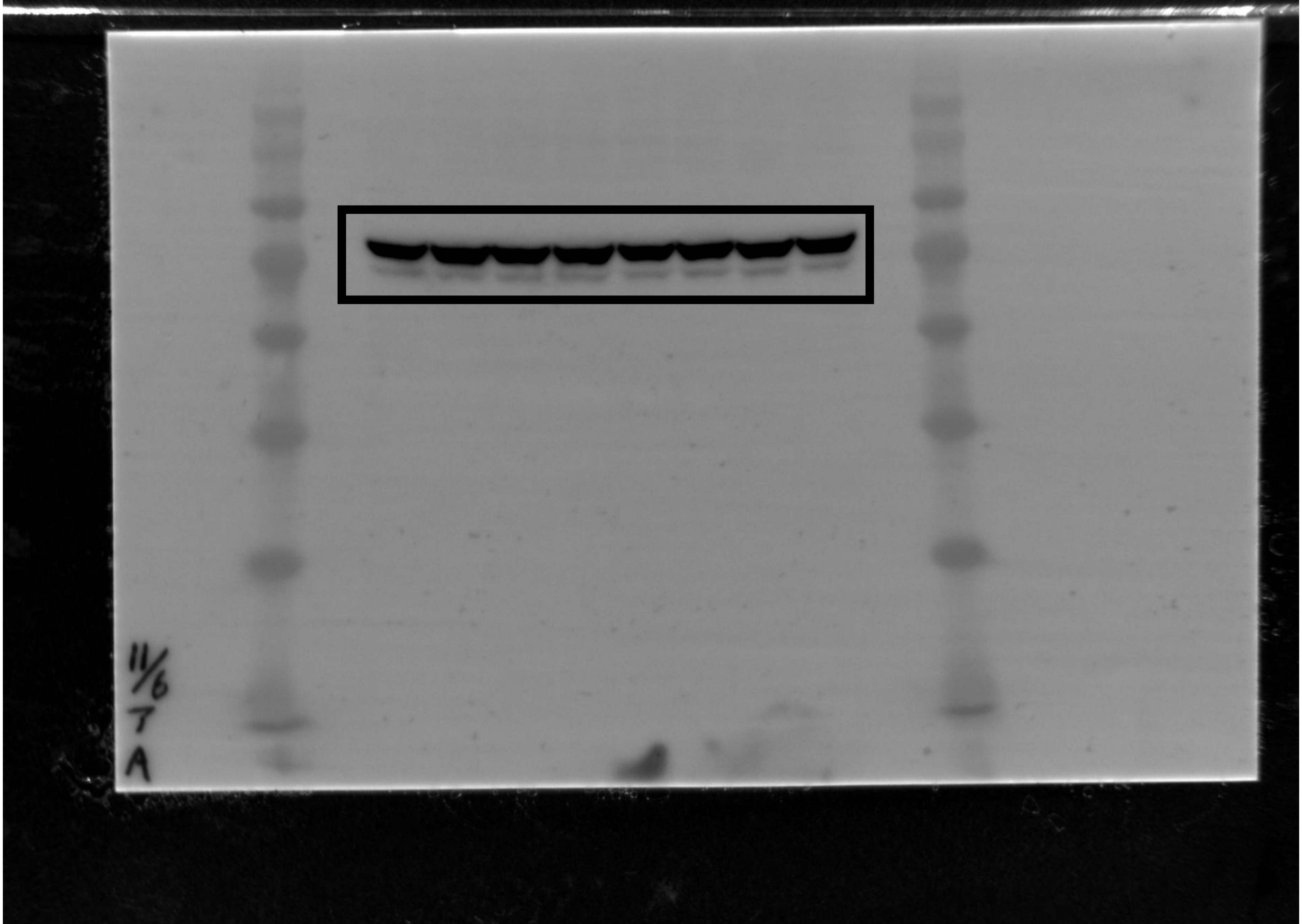

Full unedited blot for Figure 7F_1_STAT3

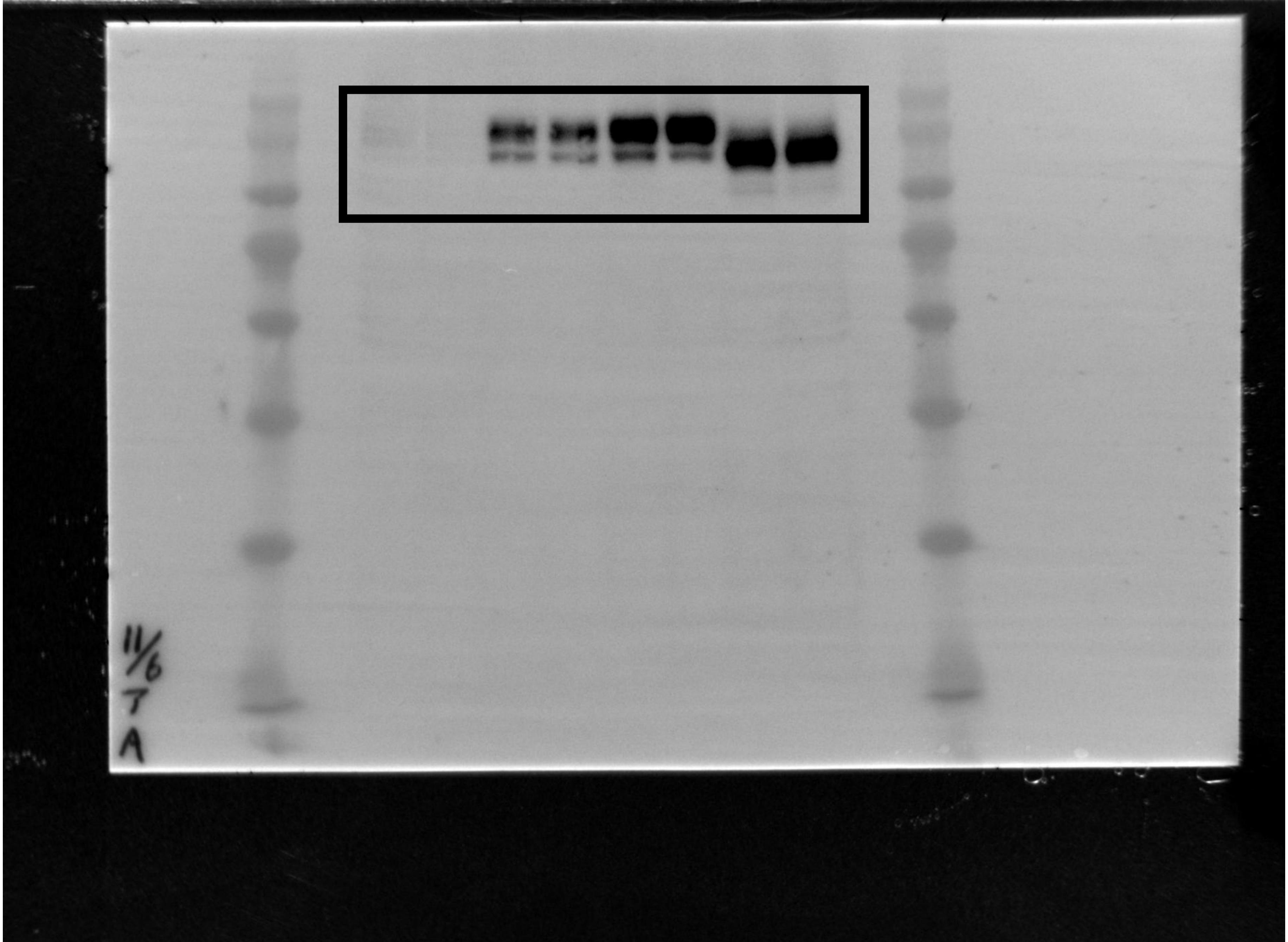

Full unedited blot for Figure 7F_1_GP130

Full unedited blot for Figure 7F_1_β-actin

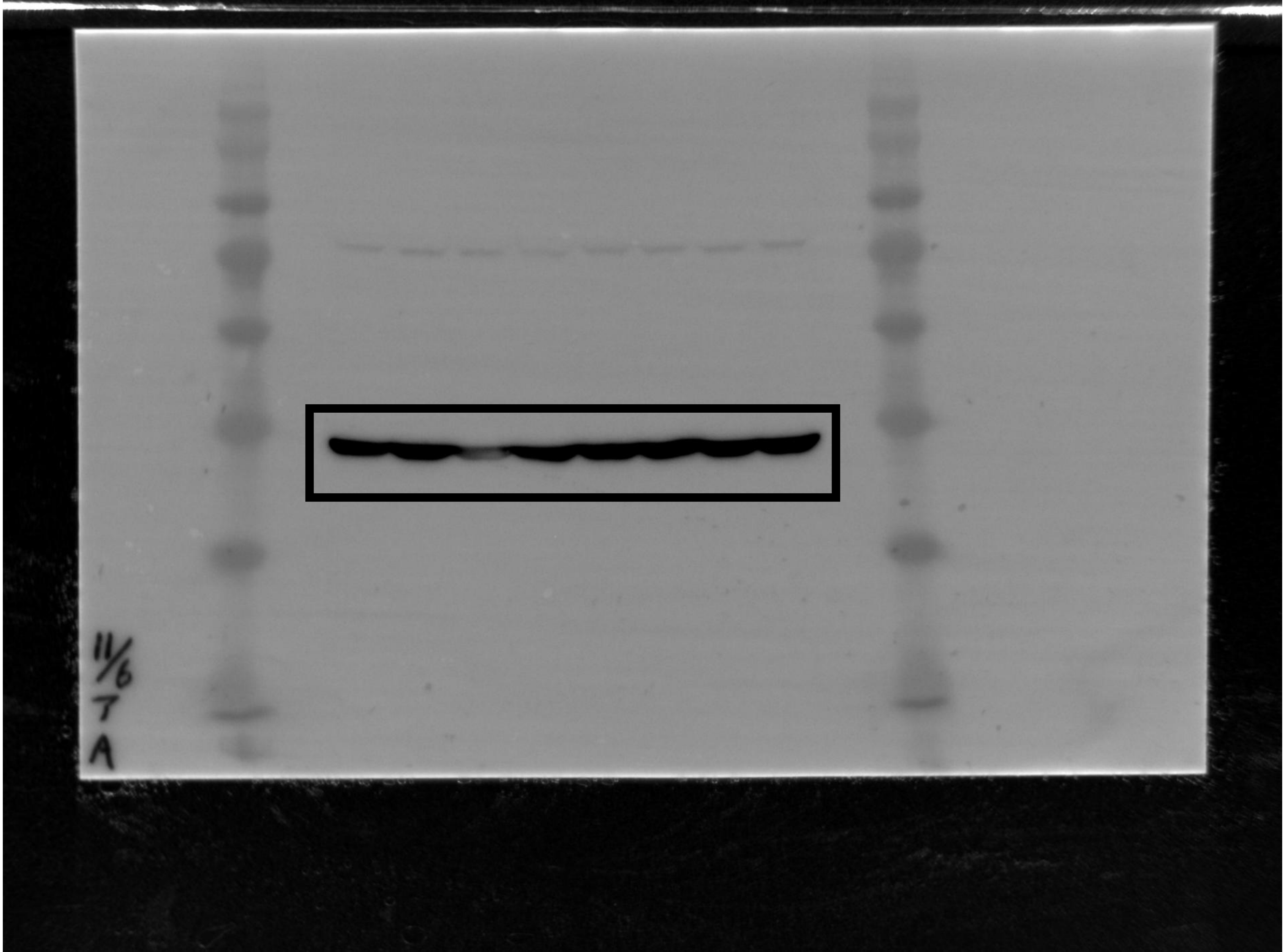

Full unedited blot for Figure 7F_2_pSTAT3

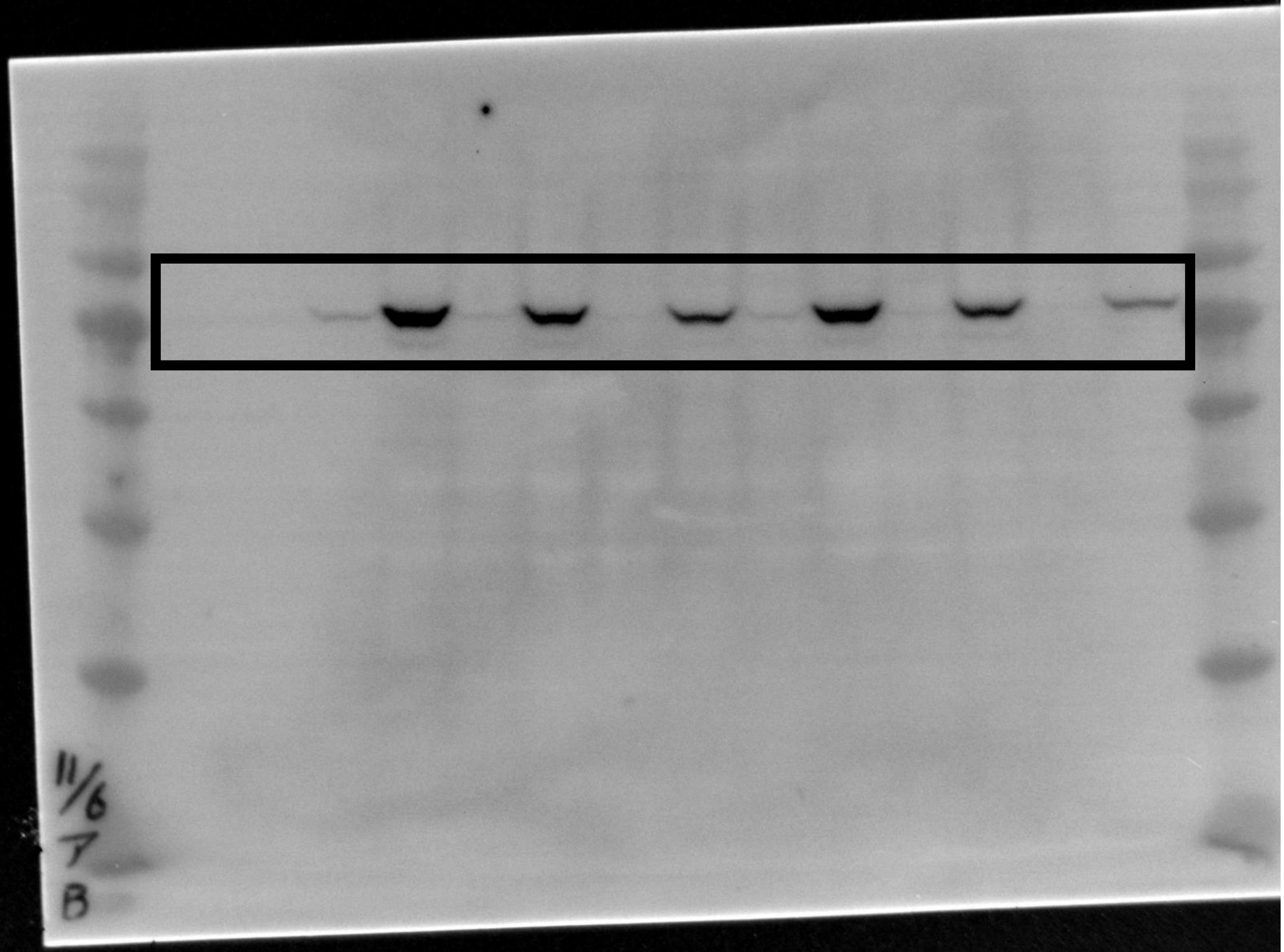

Full unedited blot for Figure 7F_2_STAT3

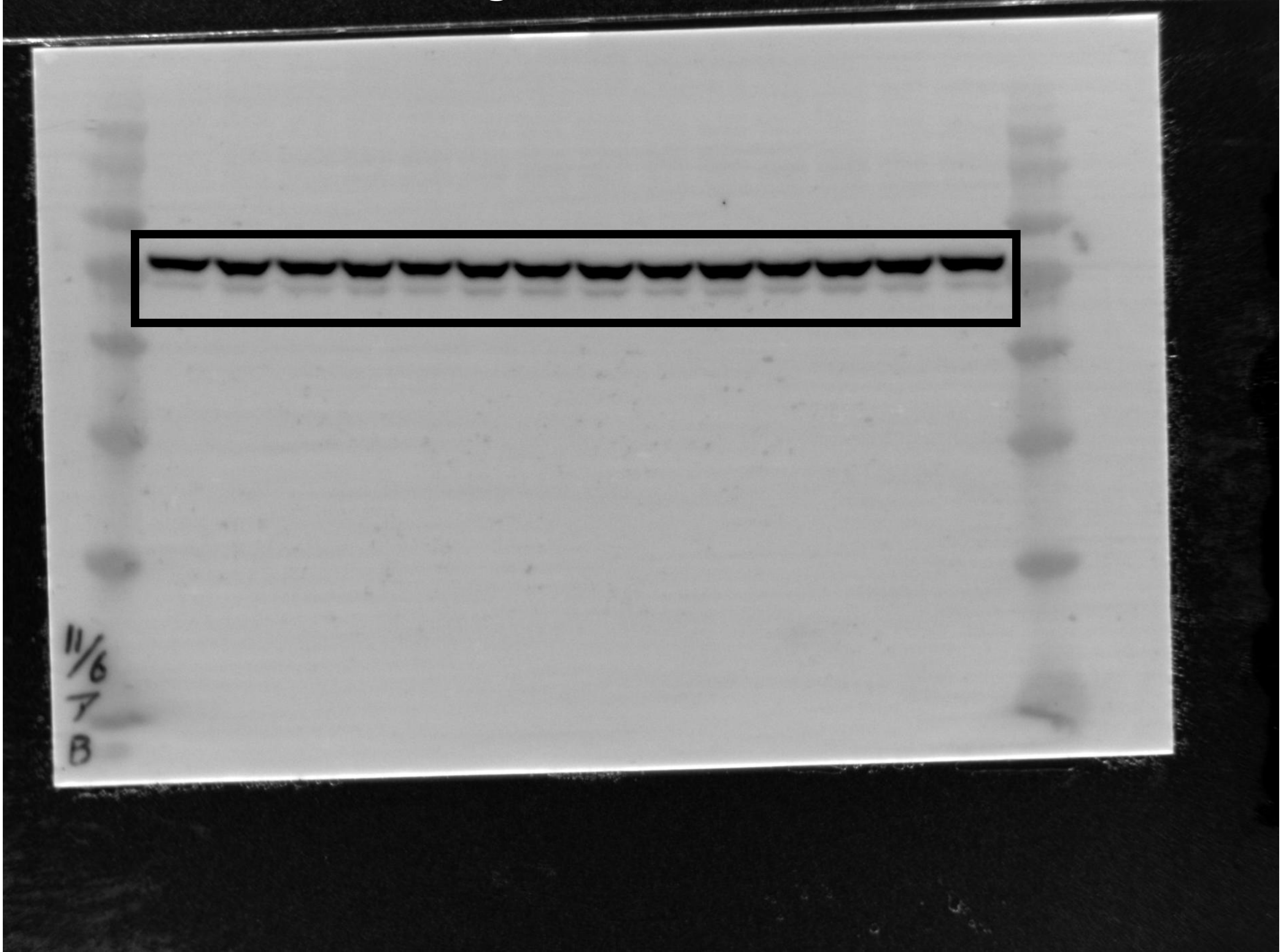

Full unedited blot for Figure 7F_2_GP130

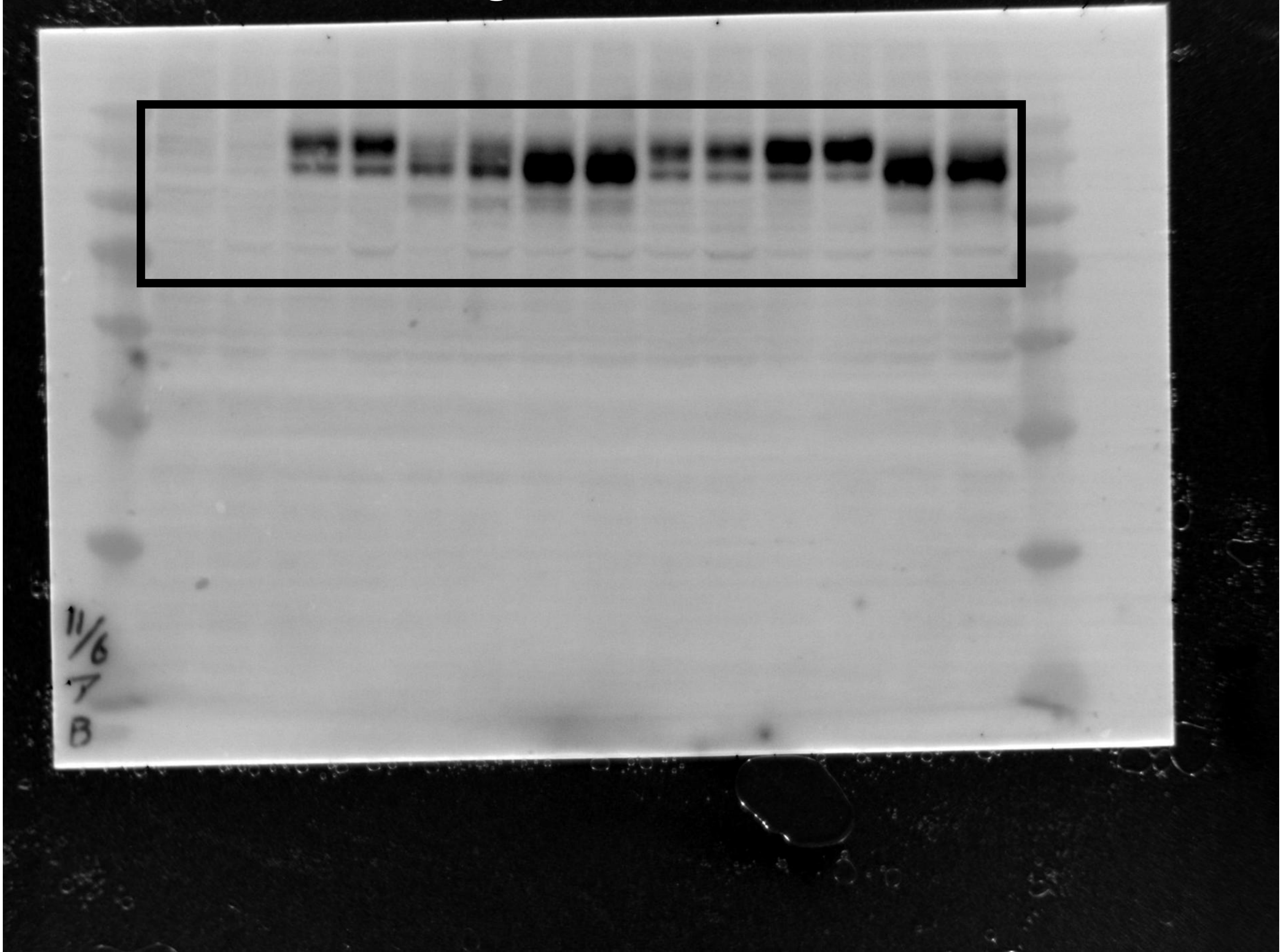

Full unedited blot for Figure 7F_2_β-actin

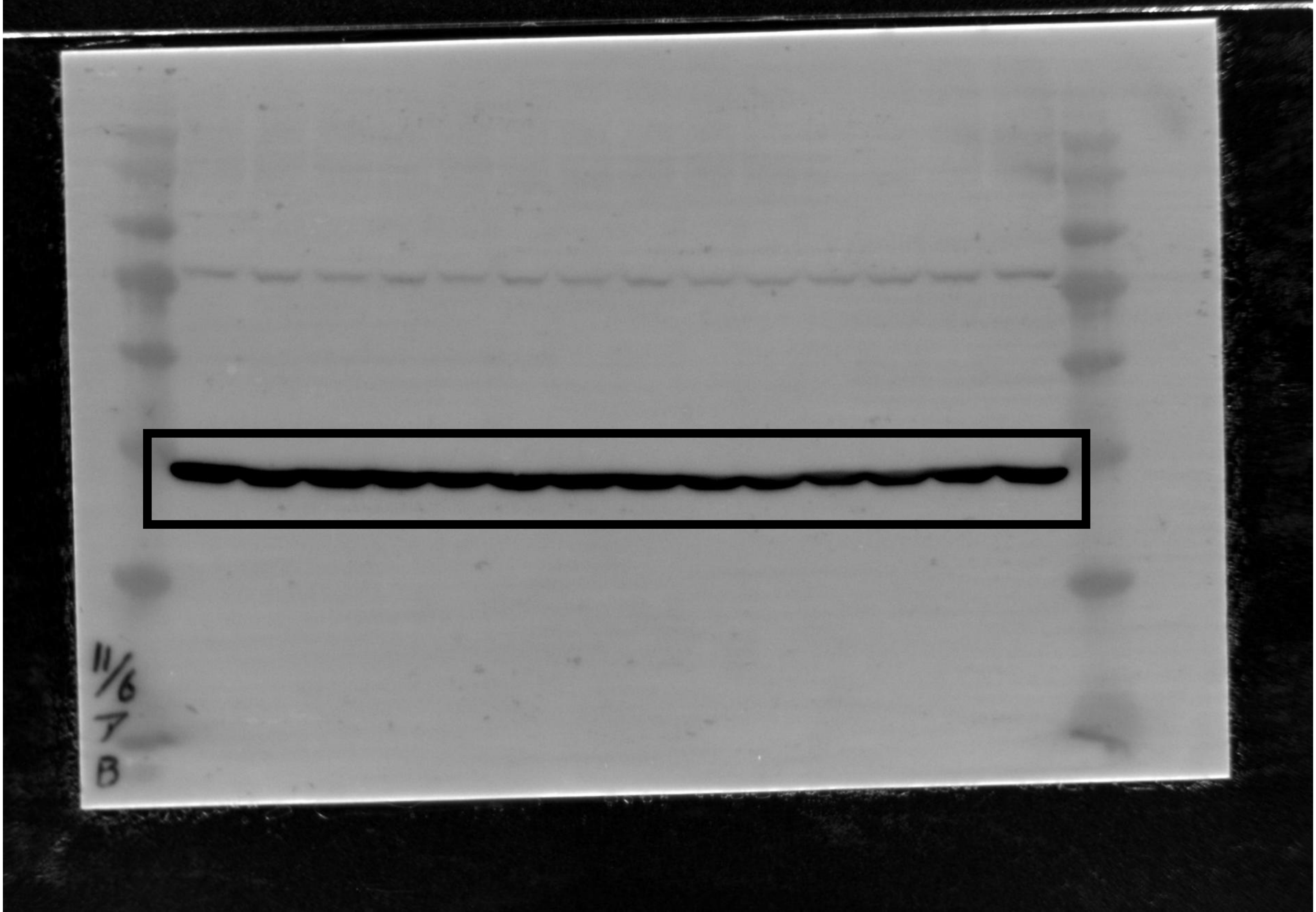

Full unedited blot for Figure S8D_cotransfection_1_pSTAT3

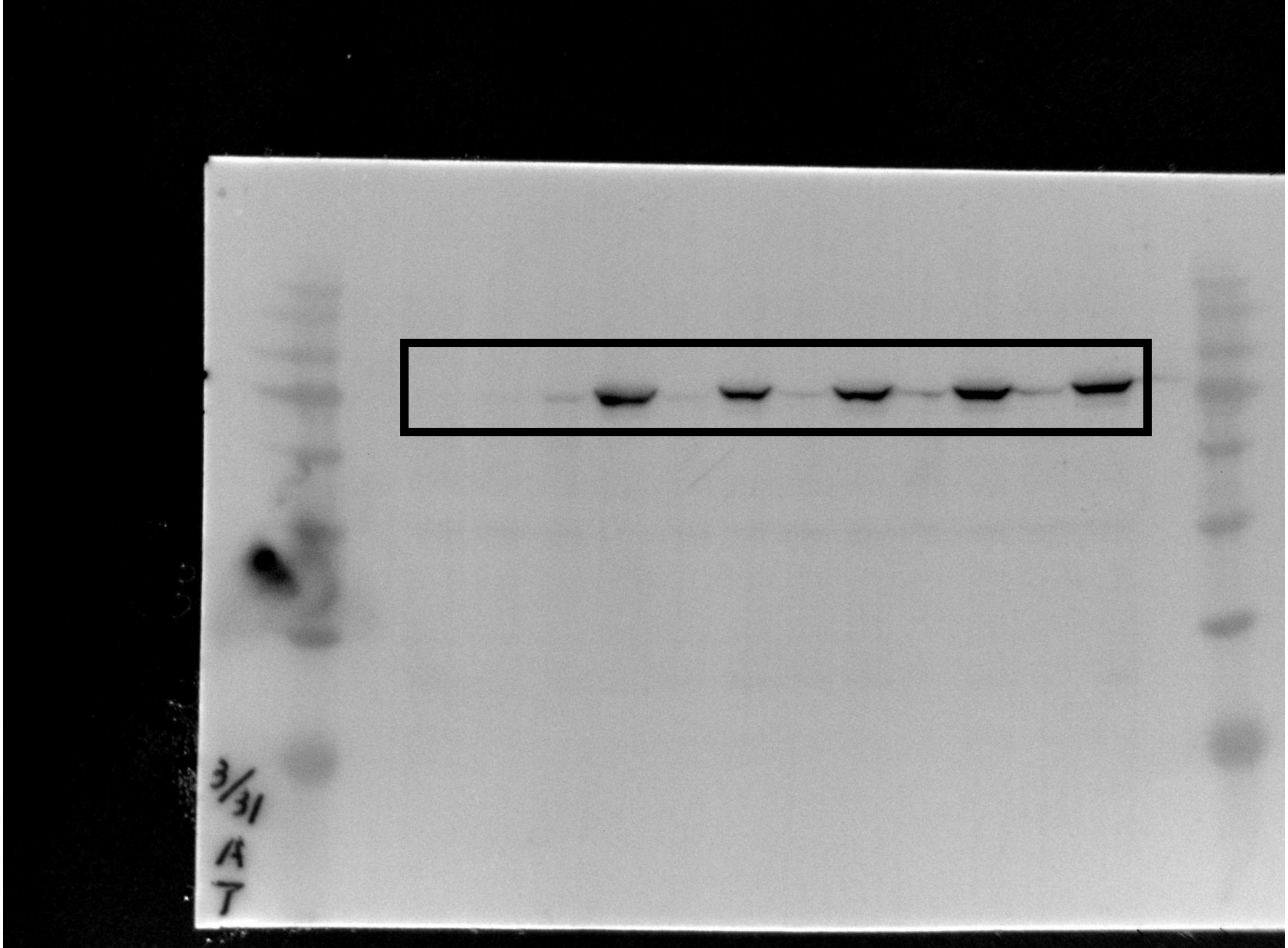

Full unedited blot for Figure S8D_cotransfection_1_STAT3

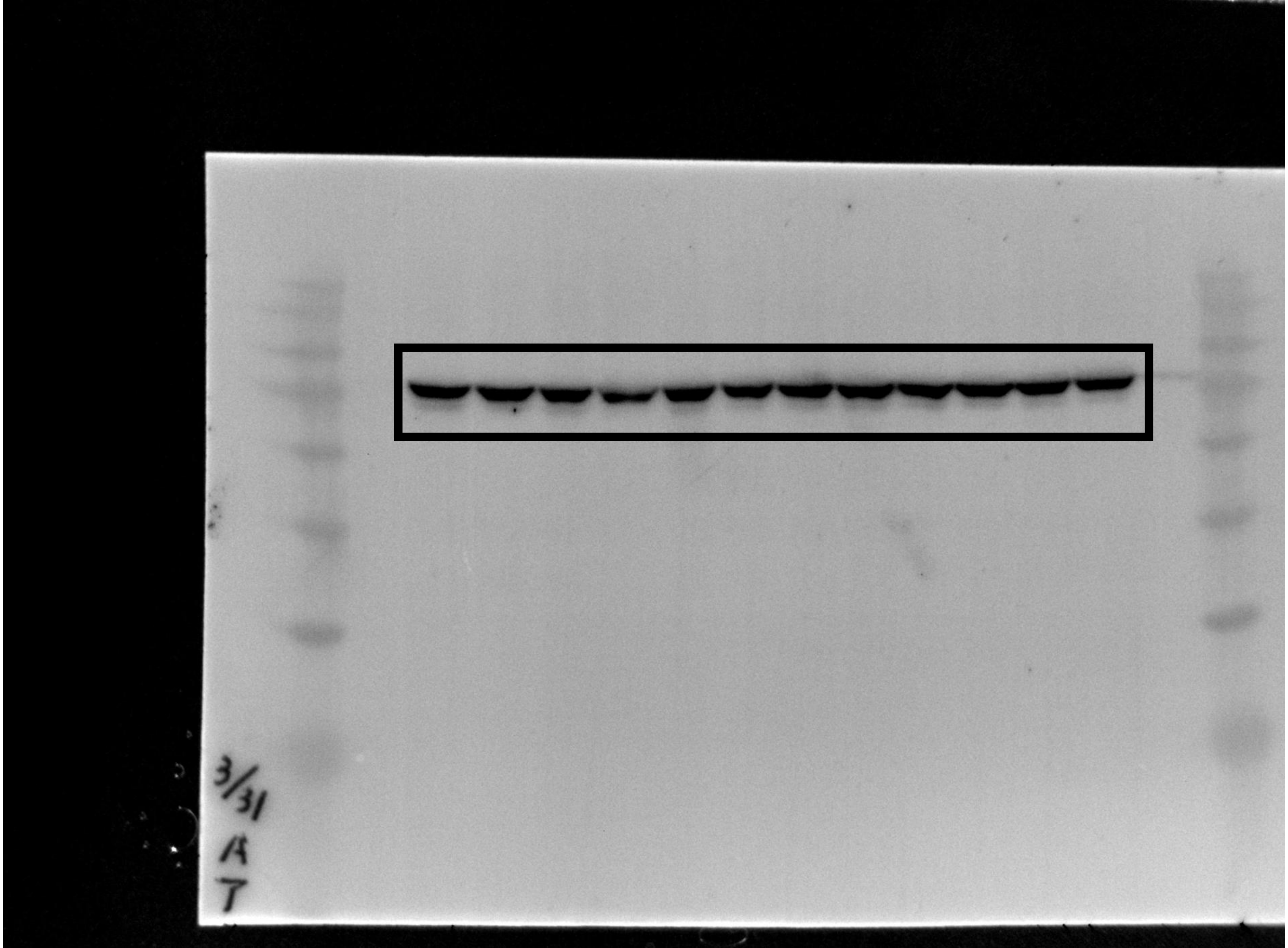

Full unedited blot for Figure S8D_cotransfection_1_GP130

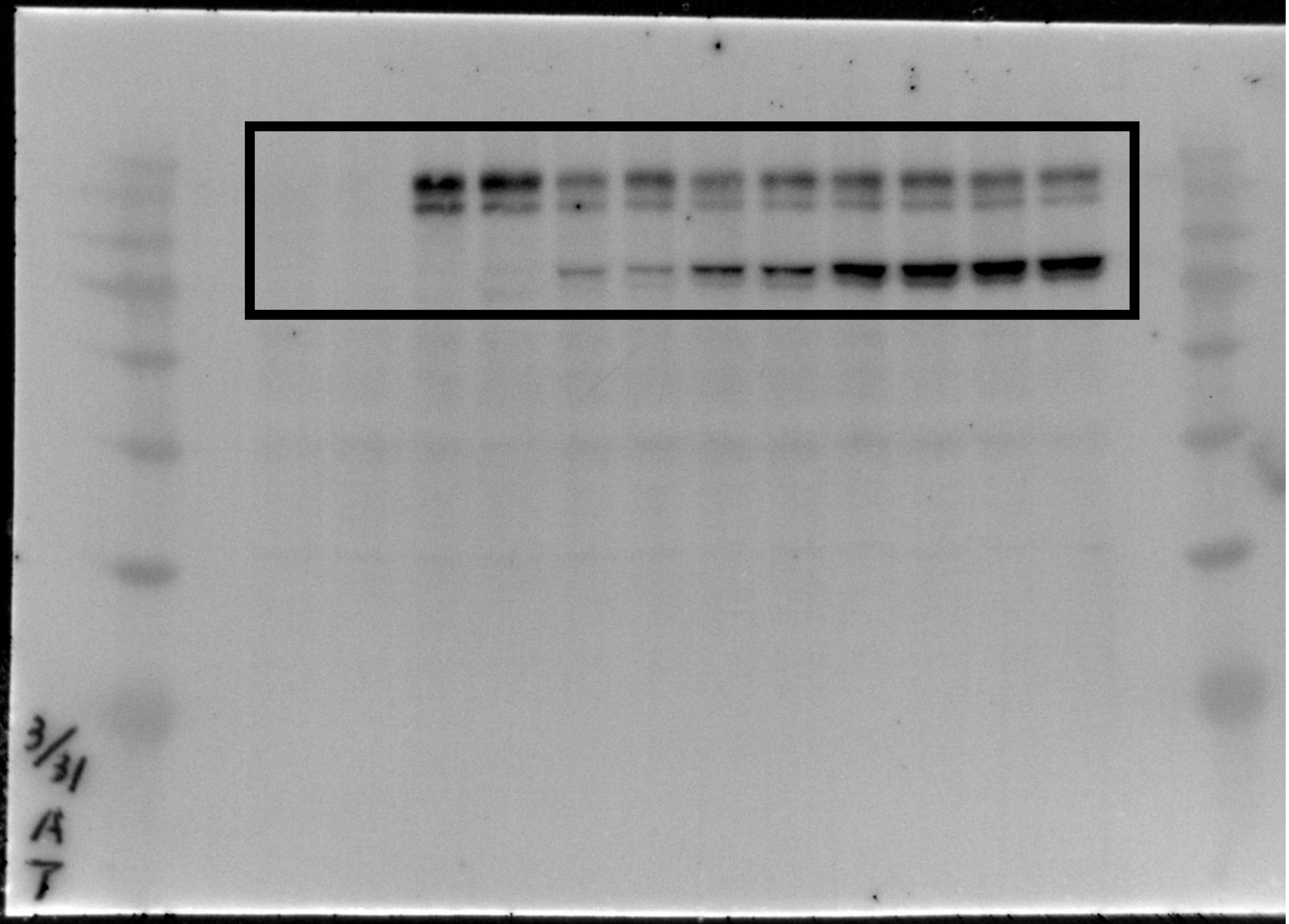

Full unedited blot for Figure S8D_cotransfection_1_β-actin

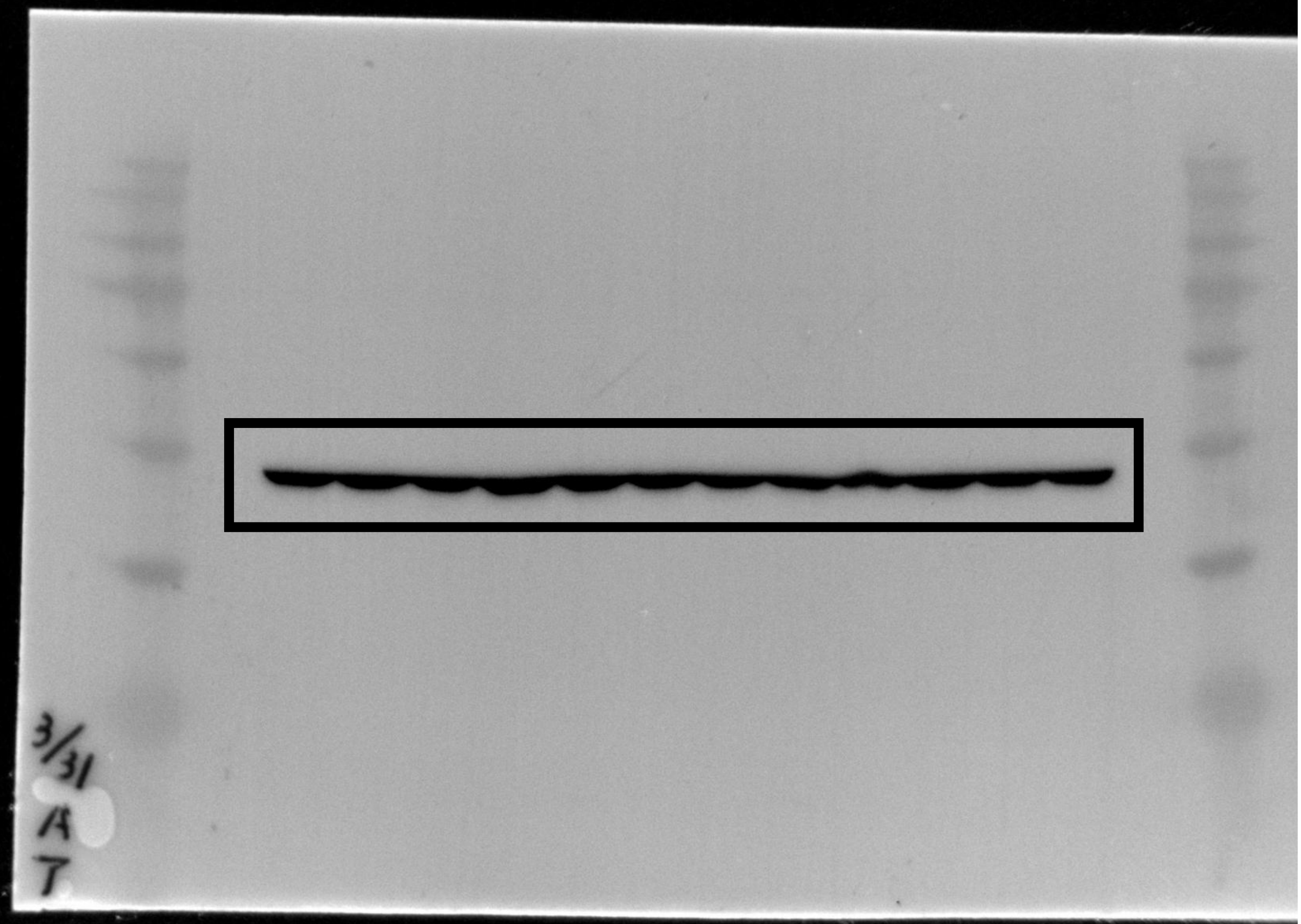

Full unedited blot for Figure S8D_cotransfection_2_pSTAT3

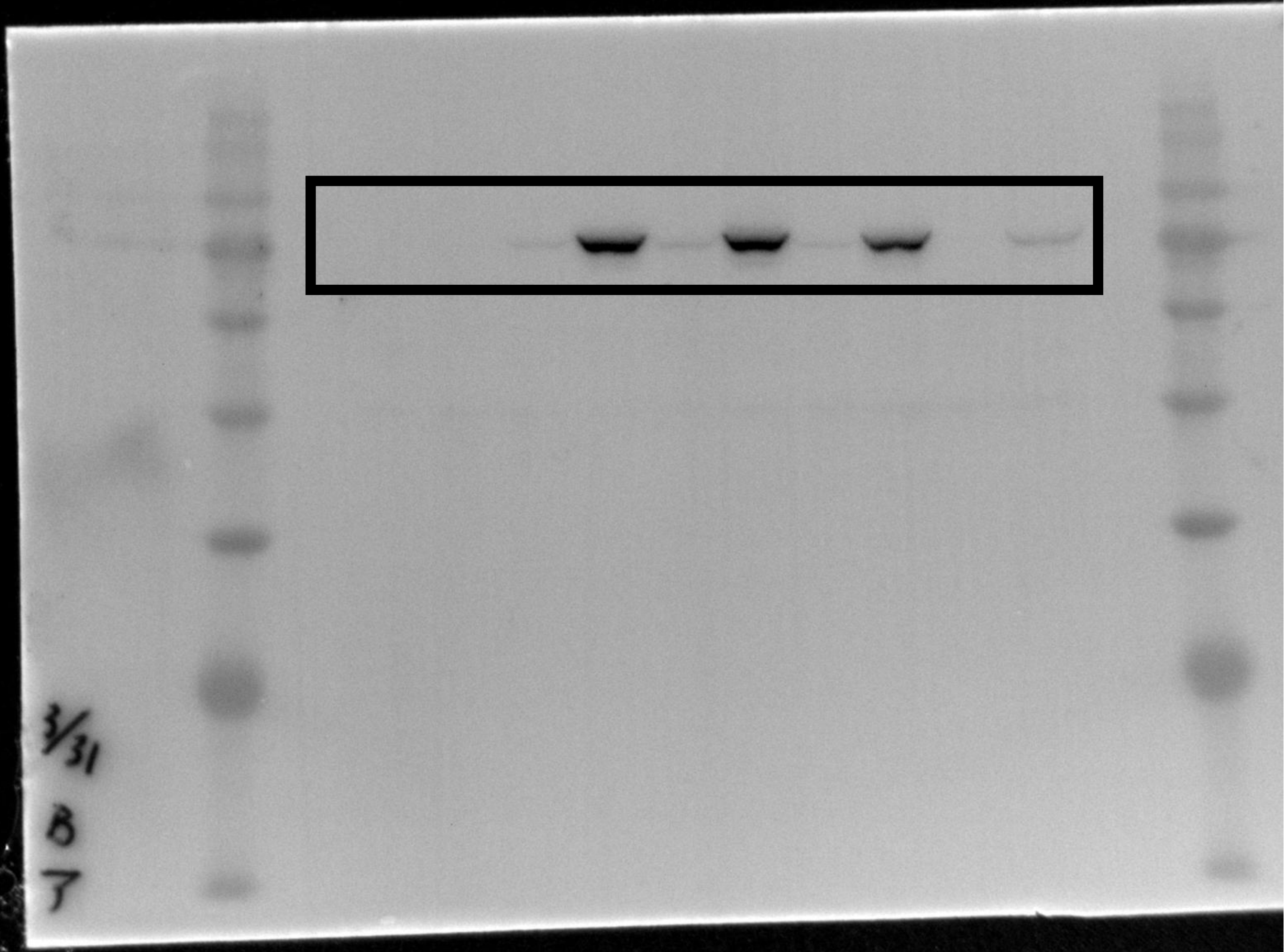

Full unedited blot for Figure S8D_cotransfection_2_STAT3

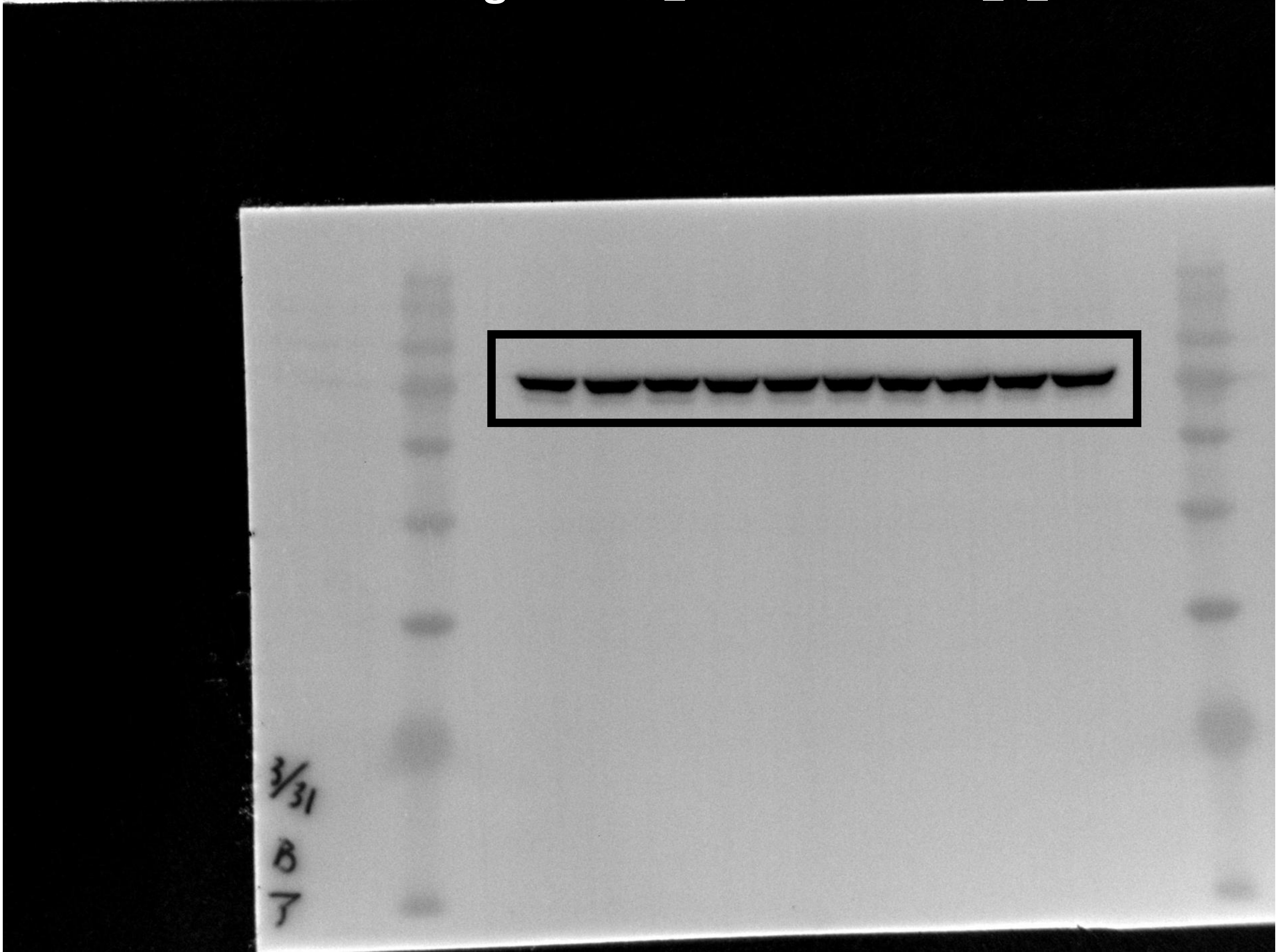

Full unedited blot for Figure S8D_cotransfection_2_GP130

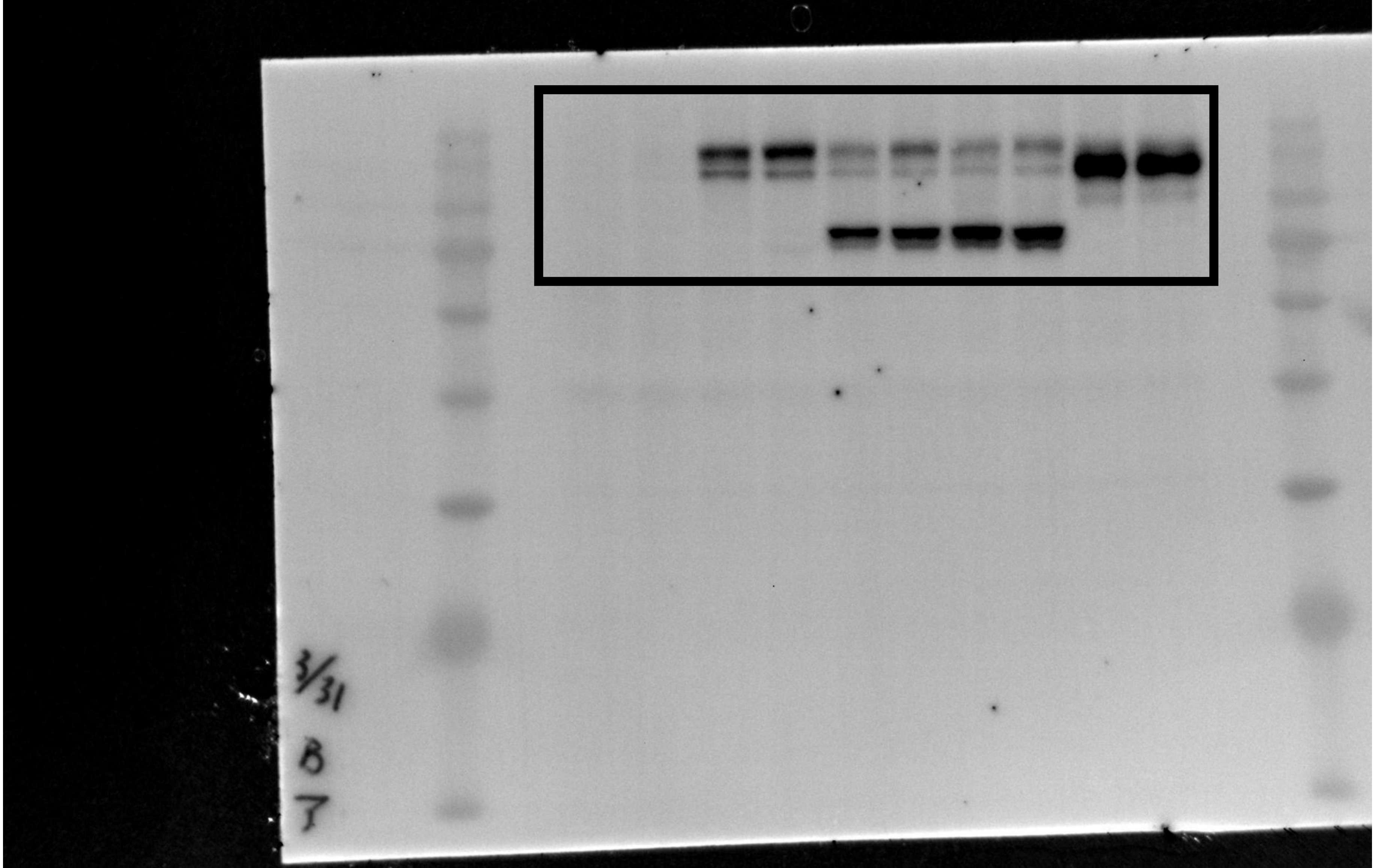

Full unedited blot for Figure S8D_cotransfection_2_β-actin

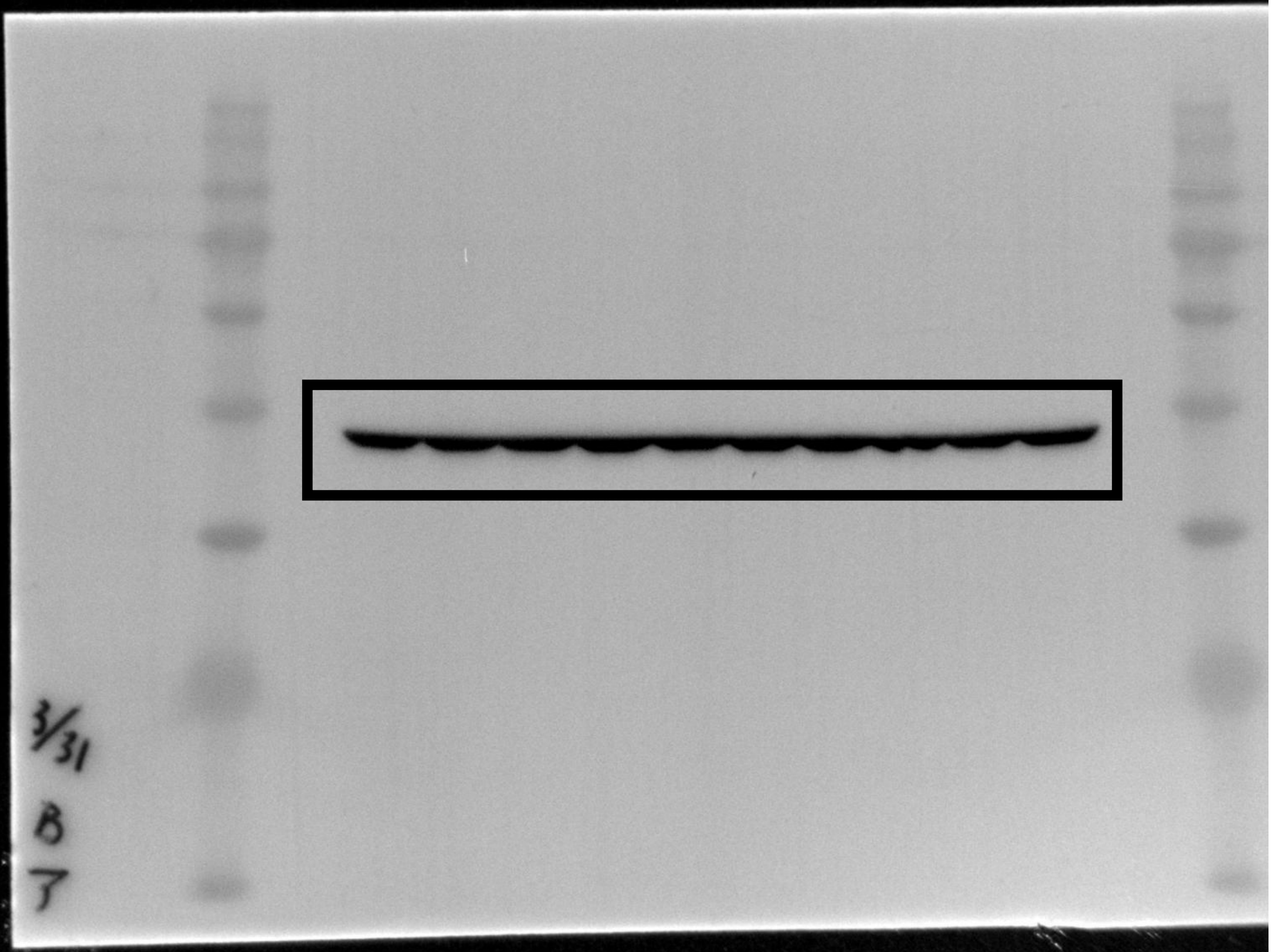

Full unedited blot for Figure S8D_single_GP130

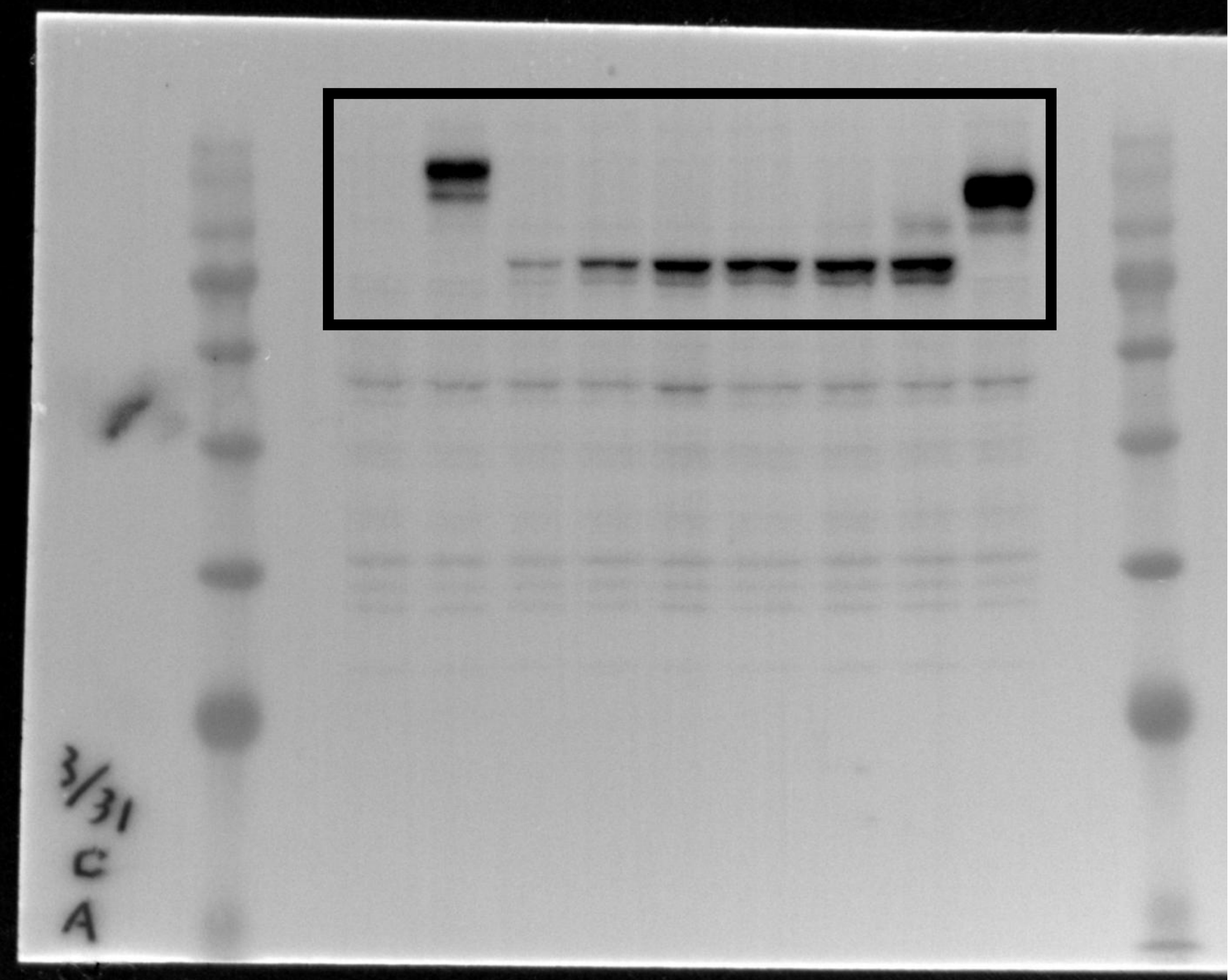

Full unedited blot for Figure S8D_single_β-actin

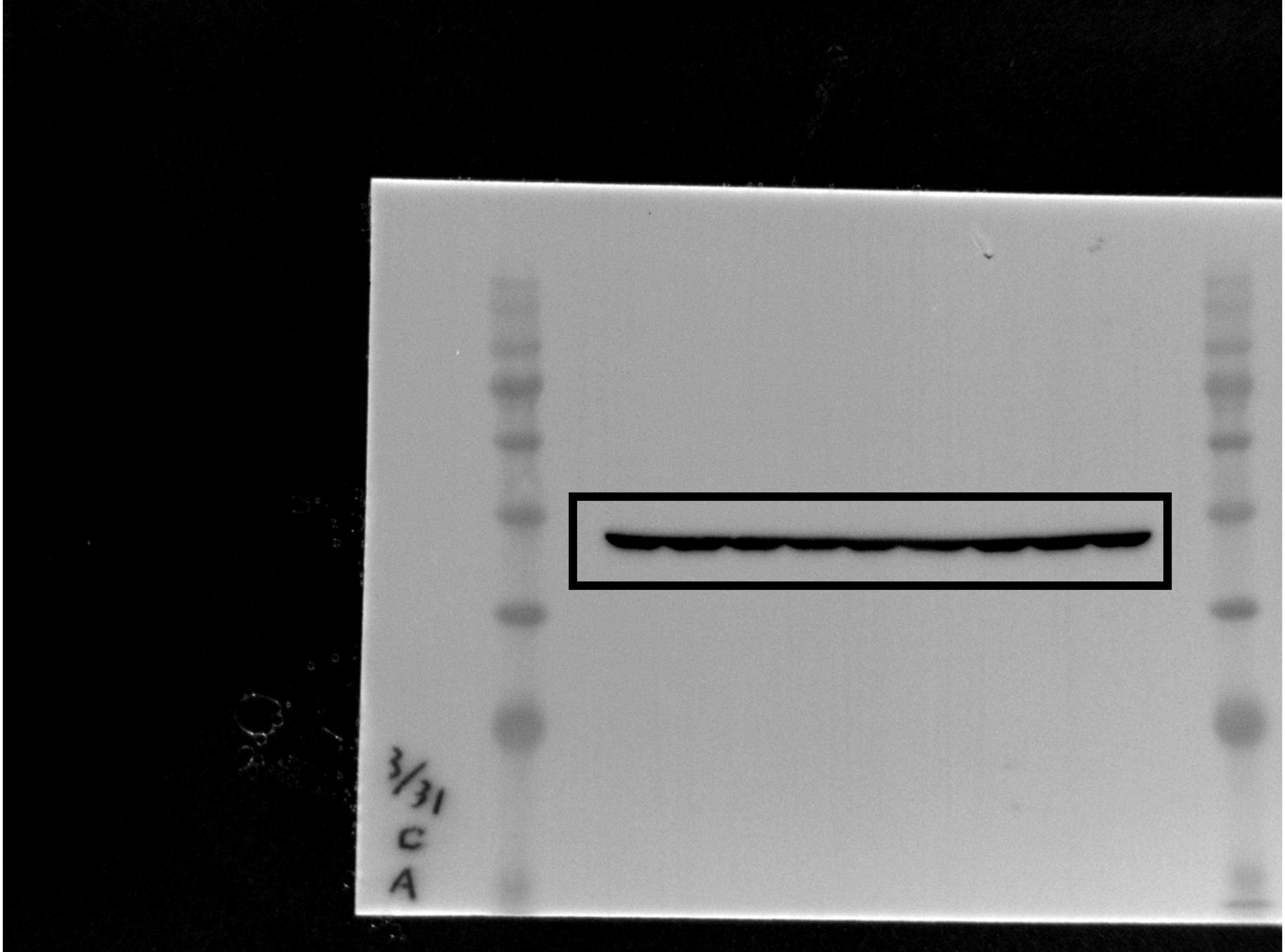

Full unedited blot for Figure S9D_cotransfection_1_pSTAT3

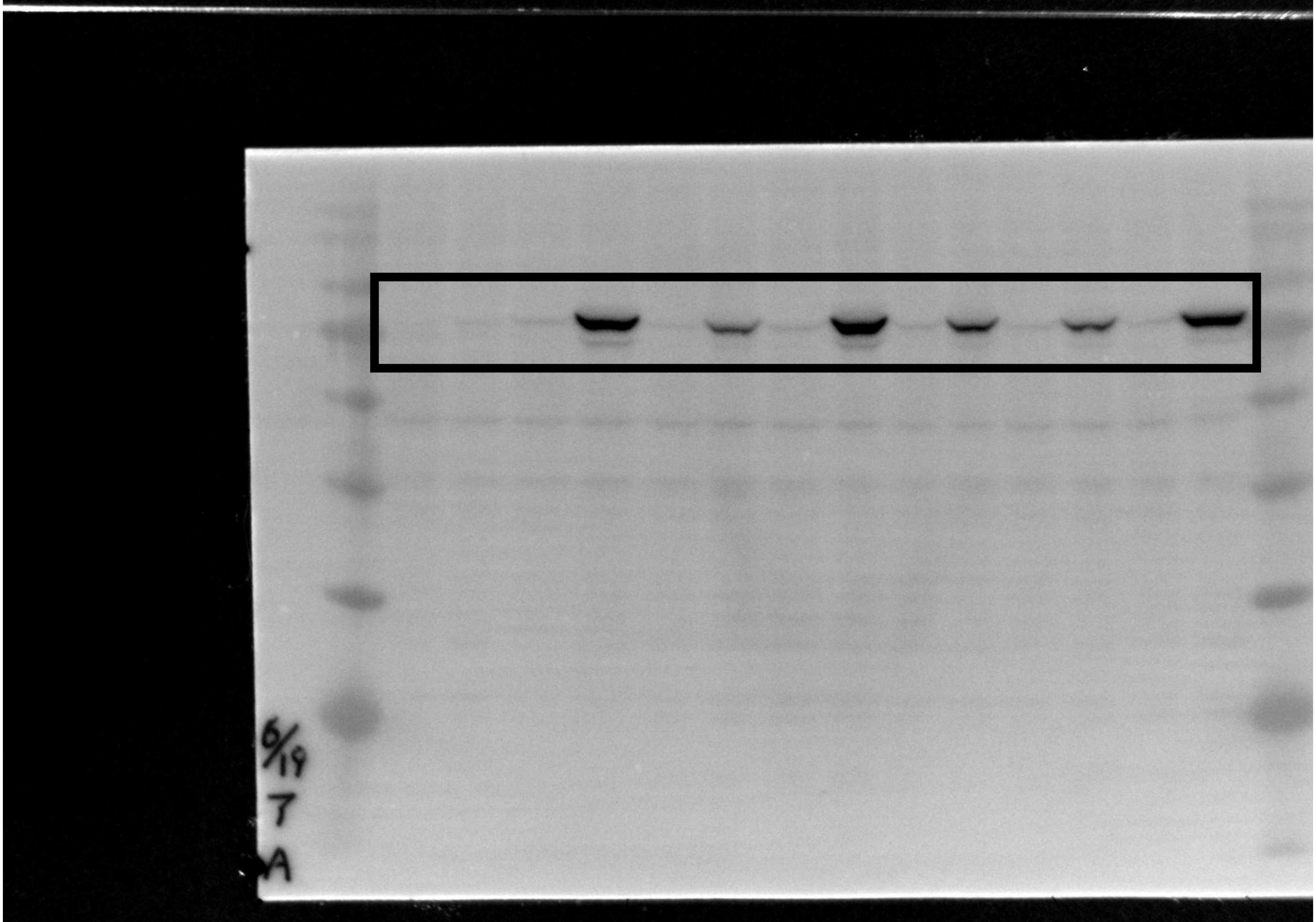

Full unedited blot for Figure S9D_cotransfection_1_STAT3

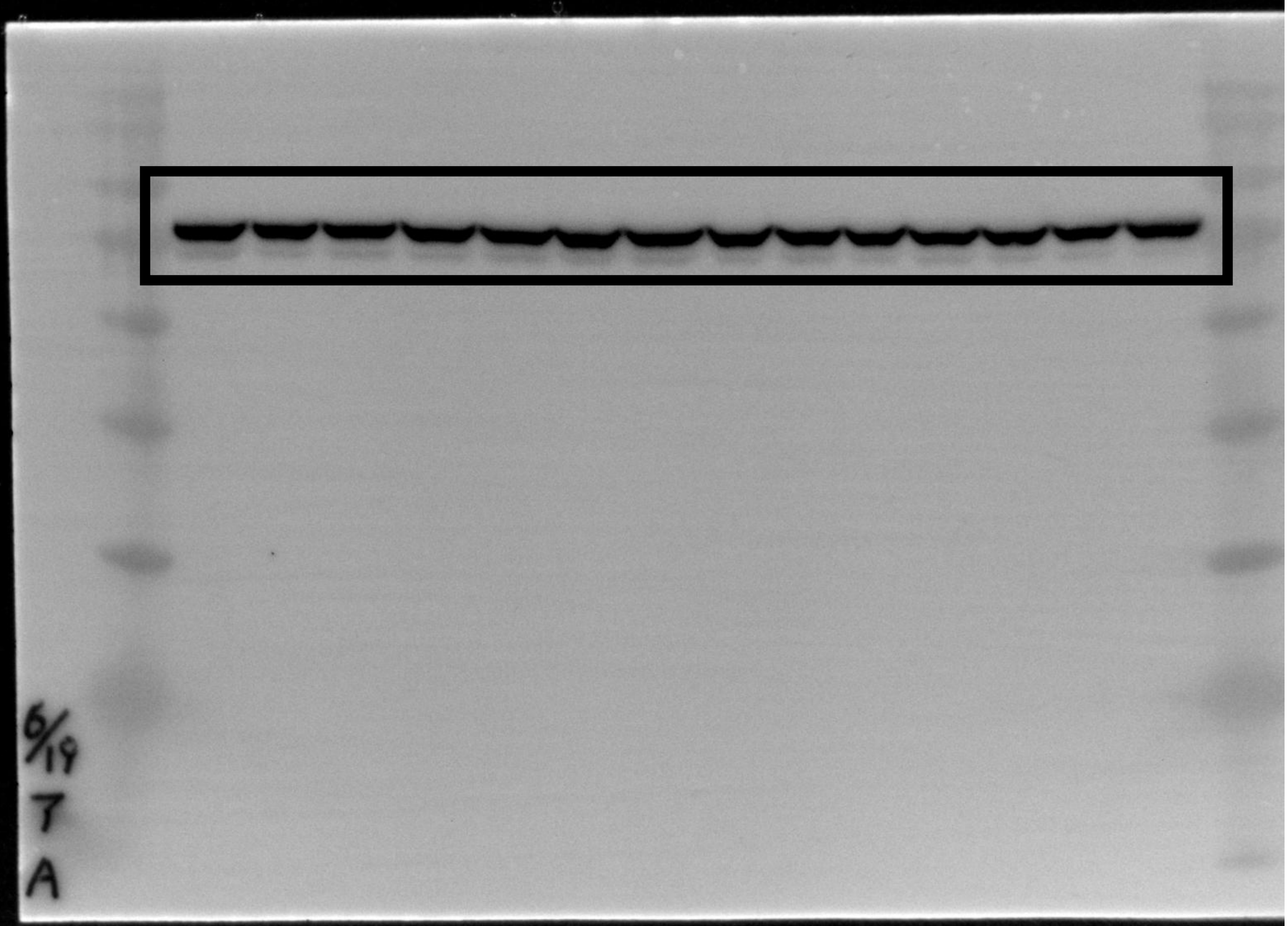

Full unedited blot for Figure S9D_cotransfection_1_GP130

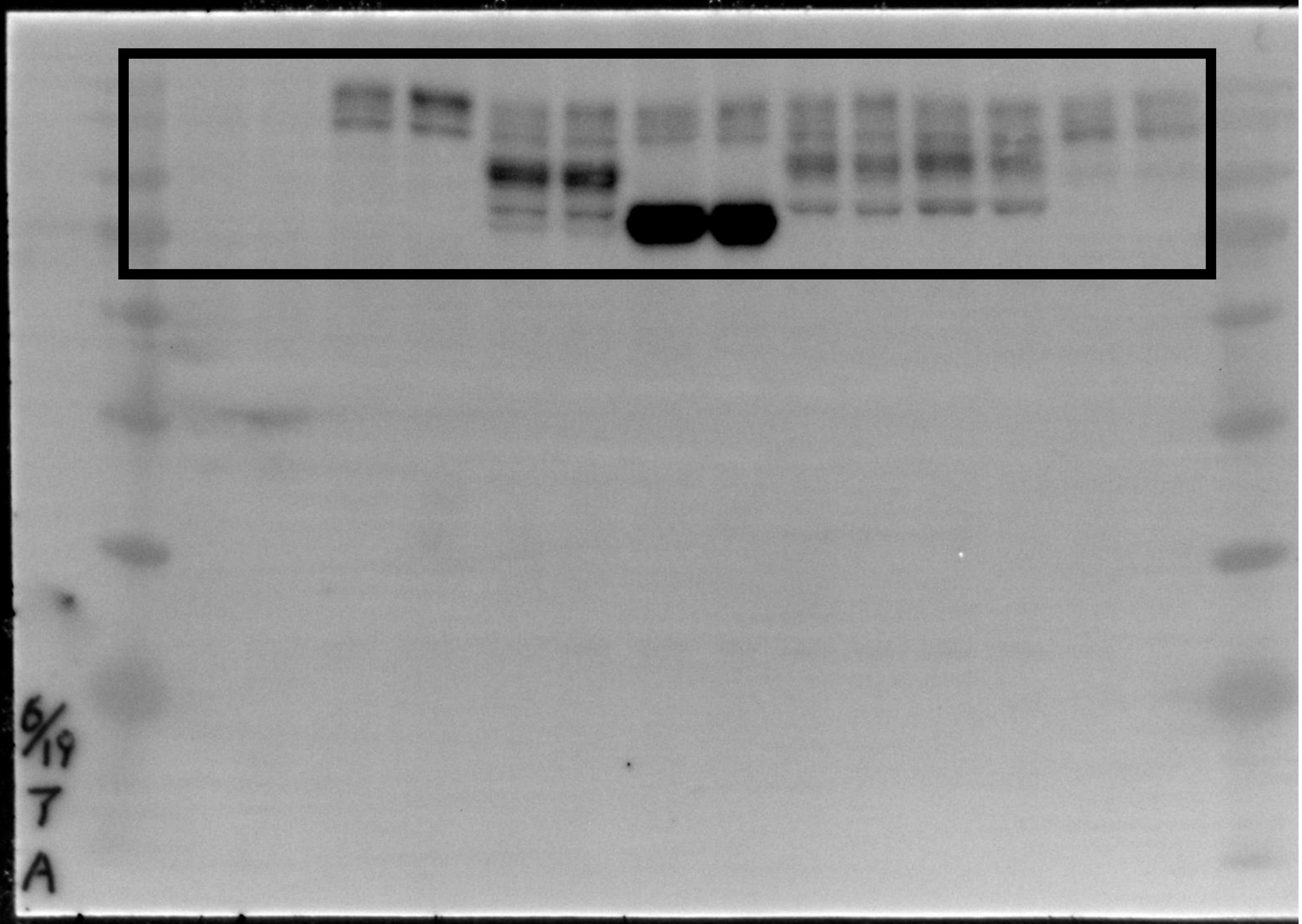

Full unedited blot for Figure S9D_cotransfection_1_β-actin

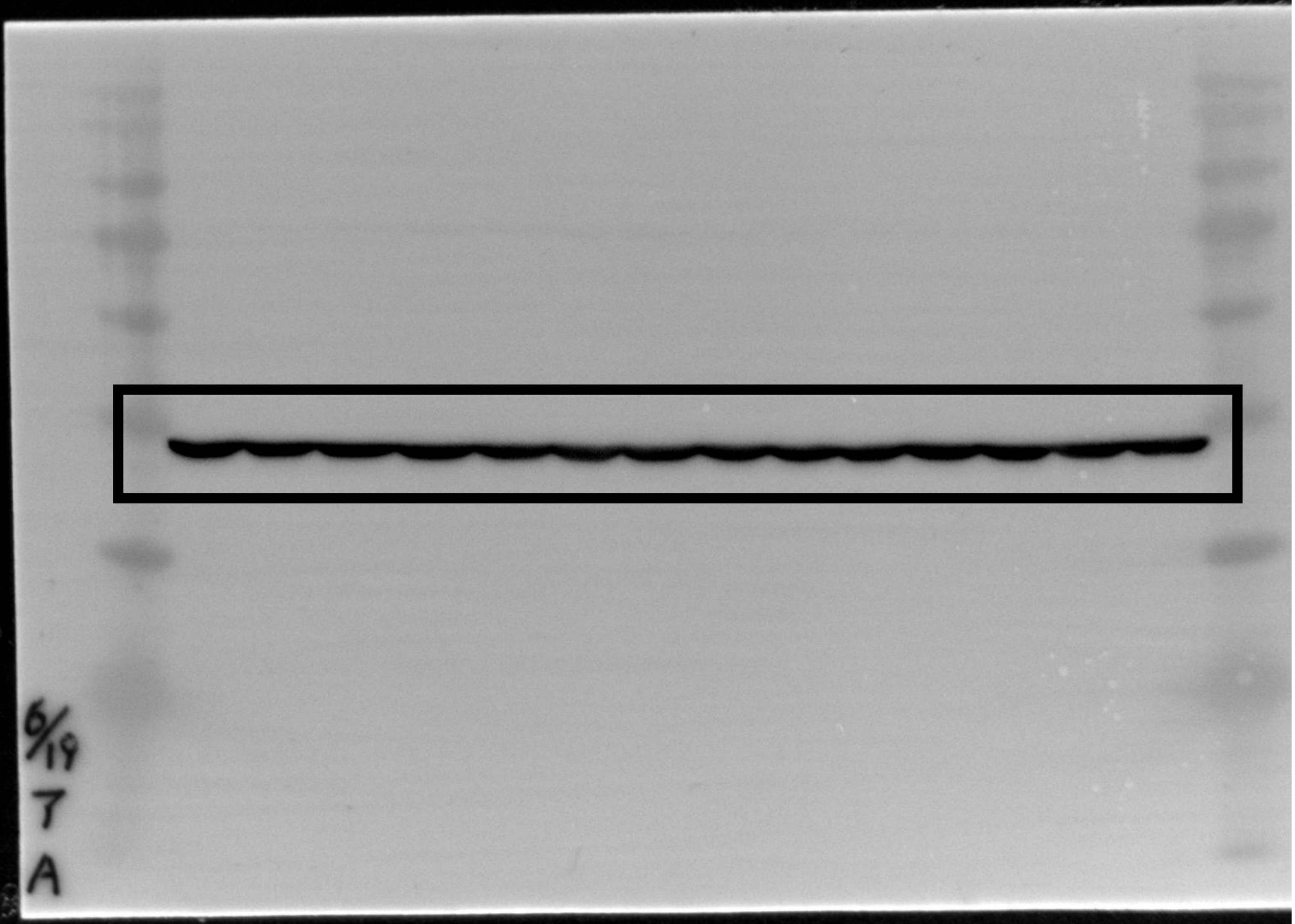

Full unedited blot for Figure S9D_cotransfection_2_pSTAT3

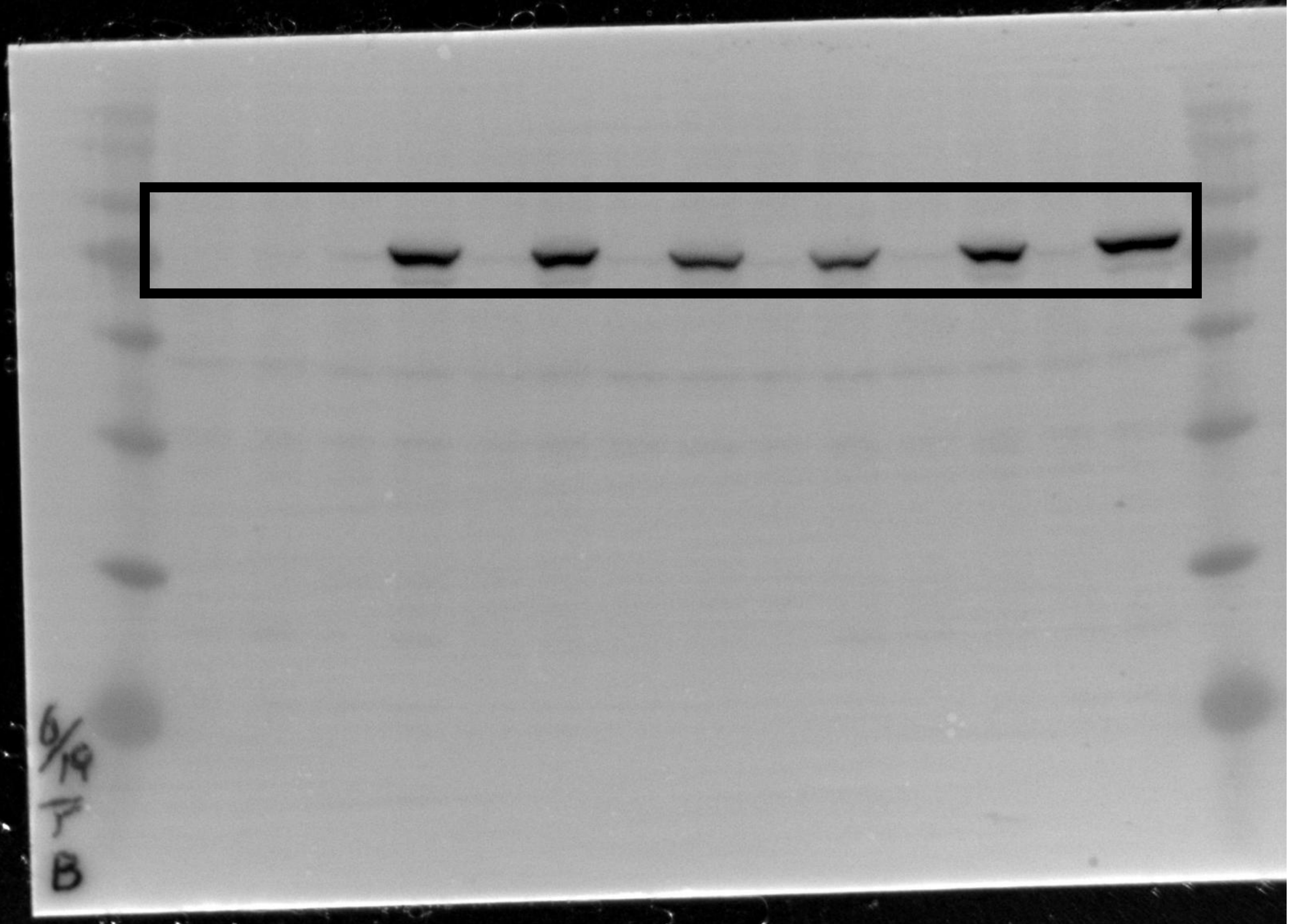

Full unedited blot for Figure S9D_cotransfection_2_STAT3

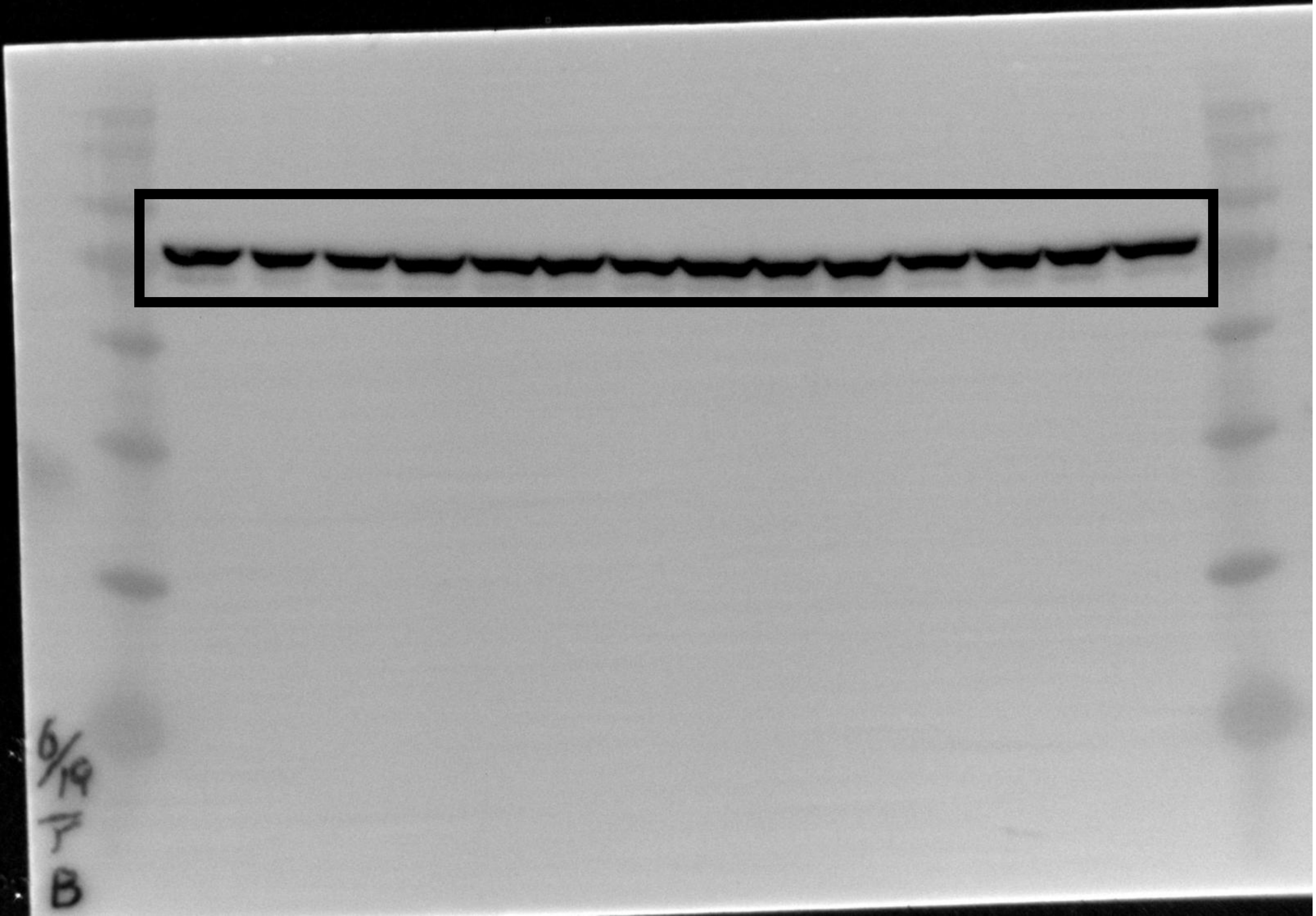

Full unedited blot for Figure S9D_cotransfection_2_GP130

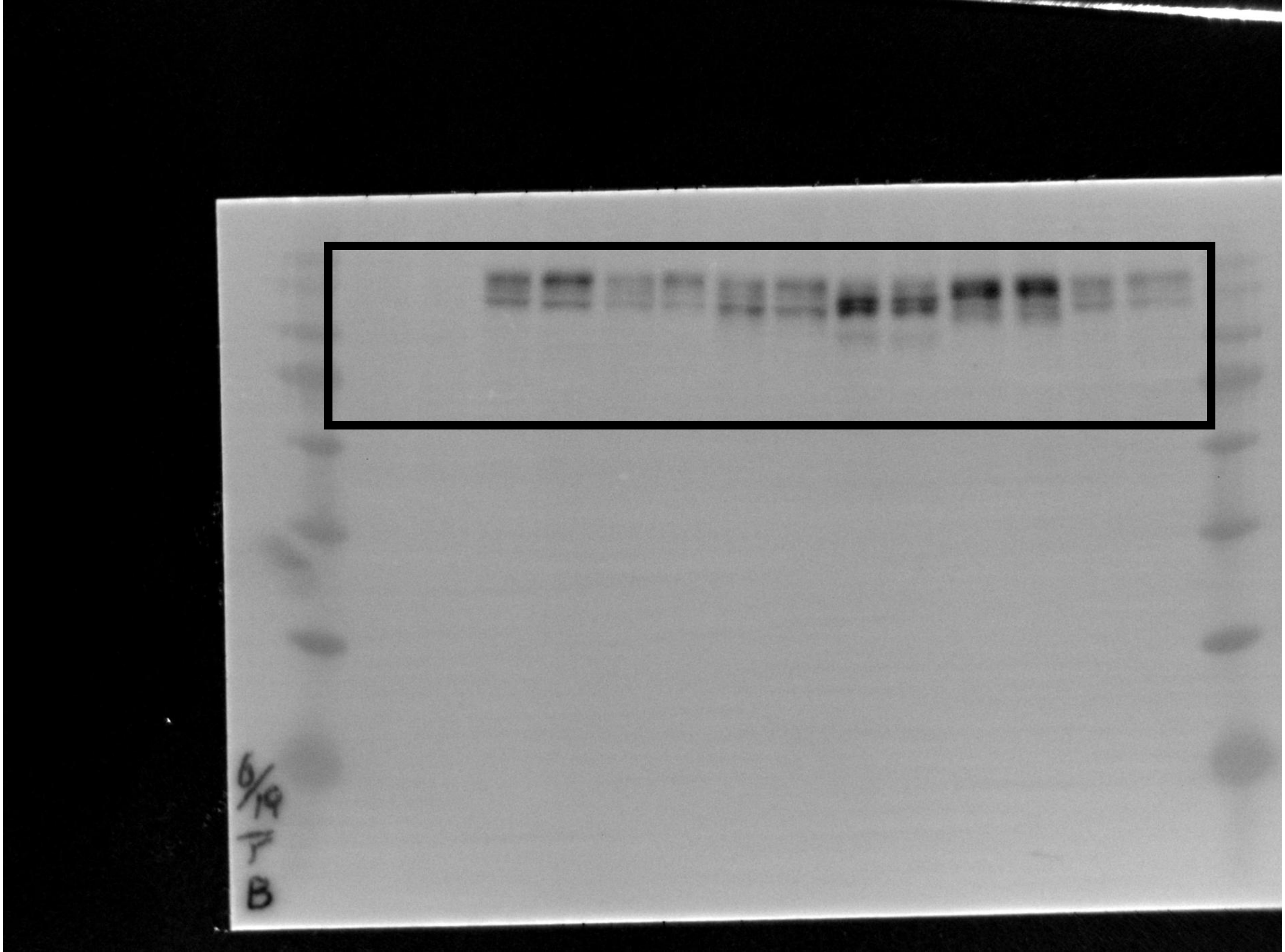

Full unedited blot for Figure S9D_cotransfection_2_β-actin

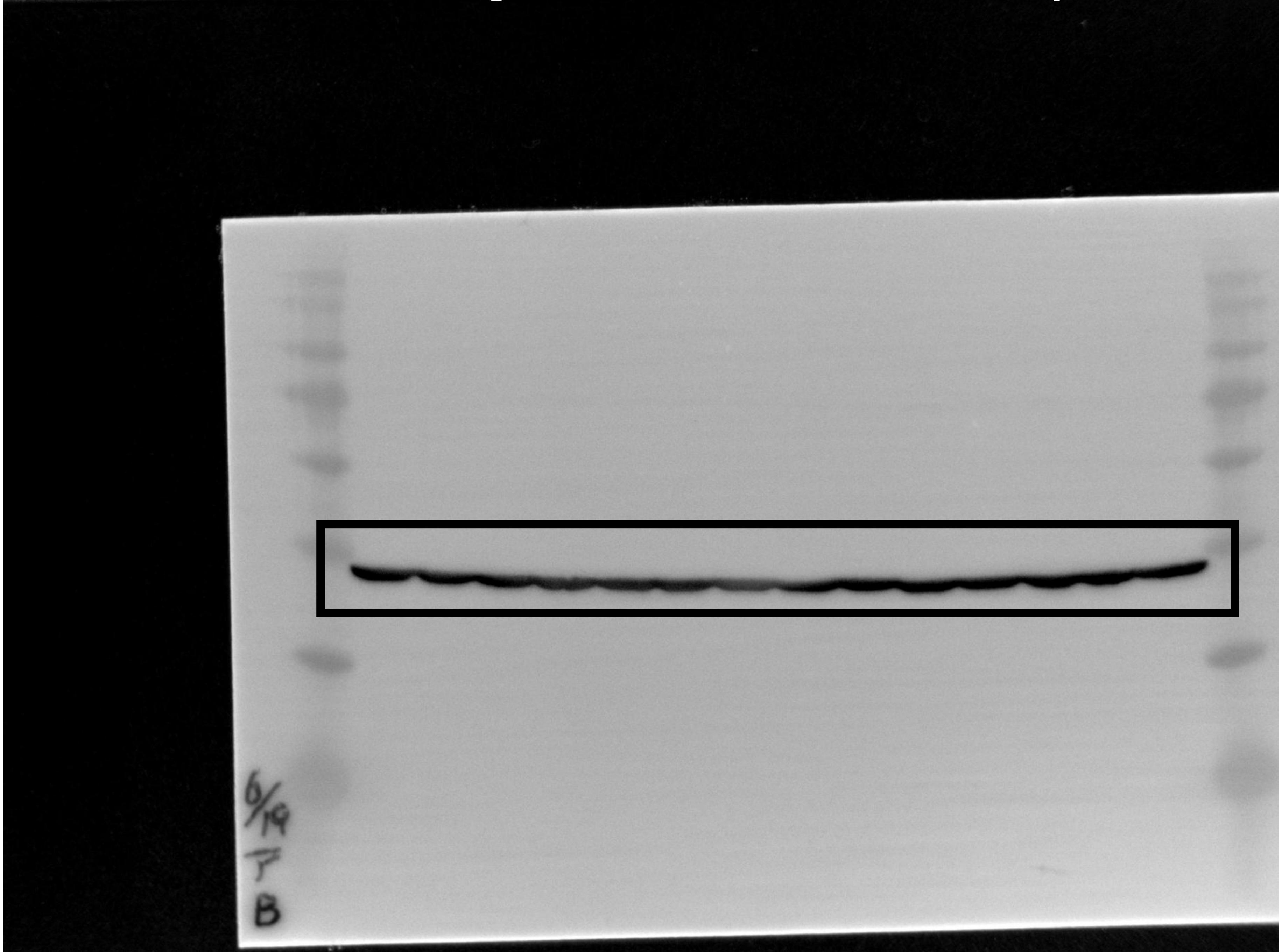

Full unedited blot for Figure S9D_cotransfection_3_pSTAT3

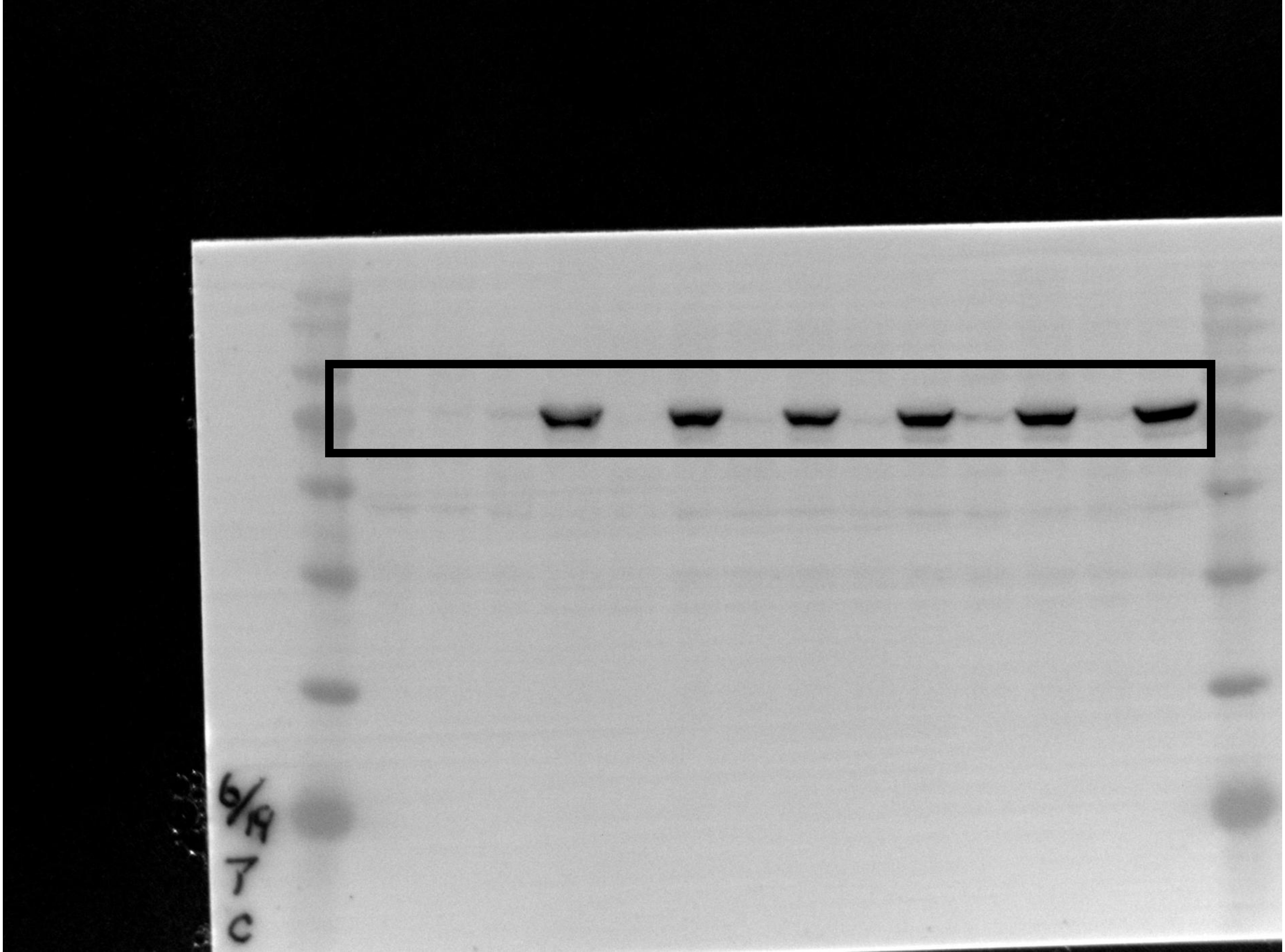

Full unedited blot for Figure S9D_cotransfection_3_STAT3

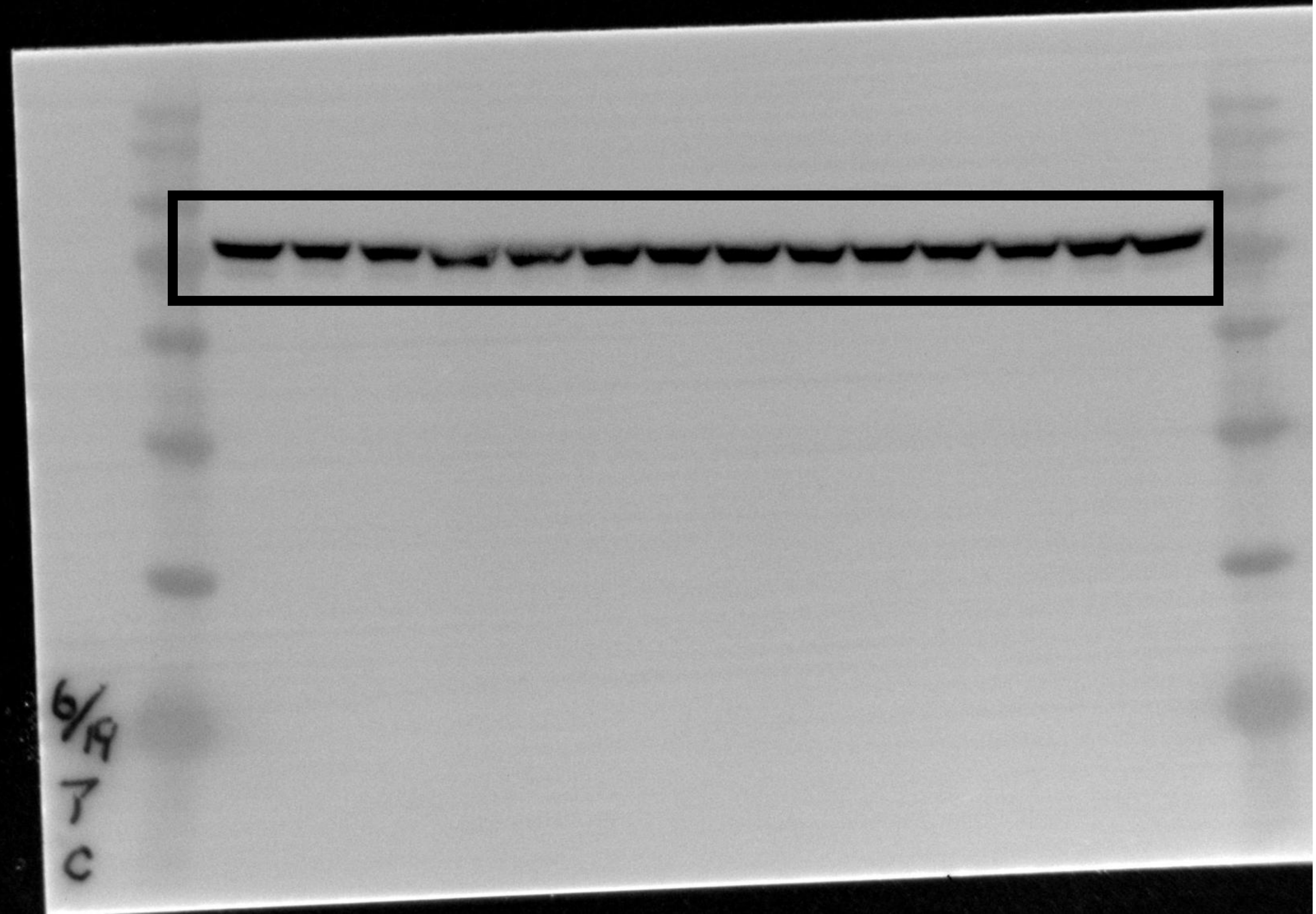

Full unedited blot for Figure S9D_cotransfection_3_GP130

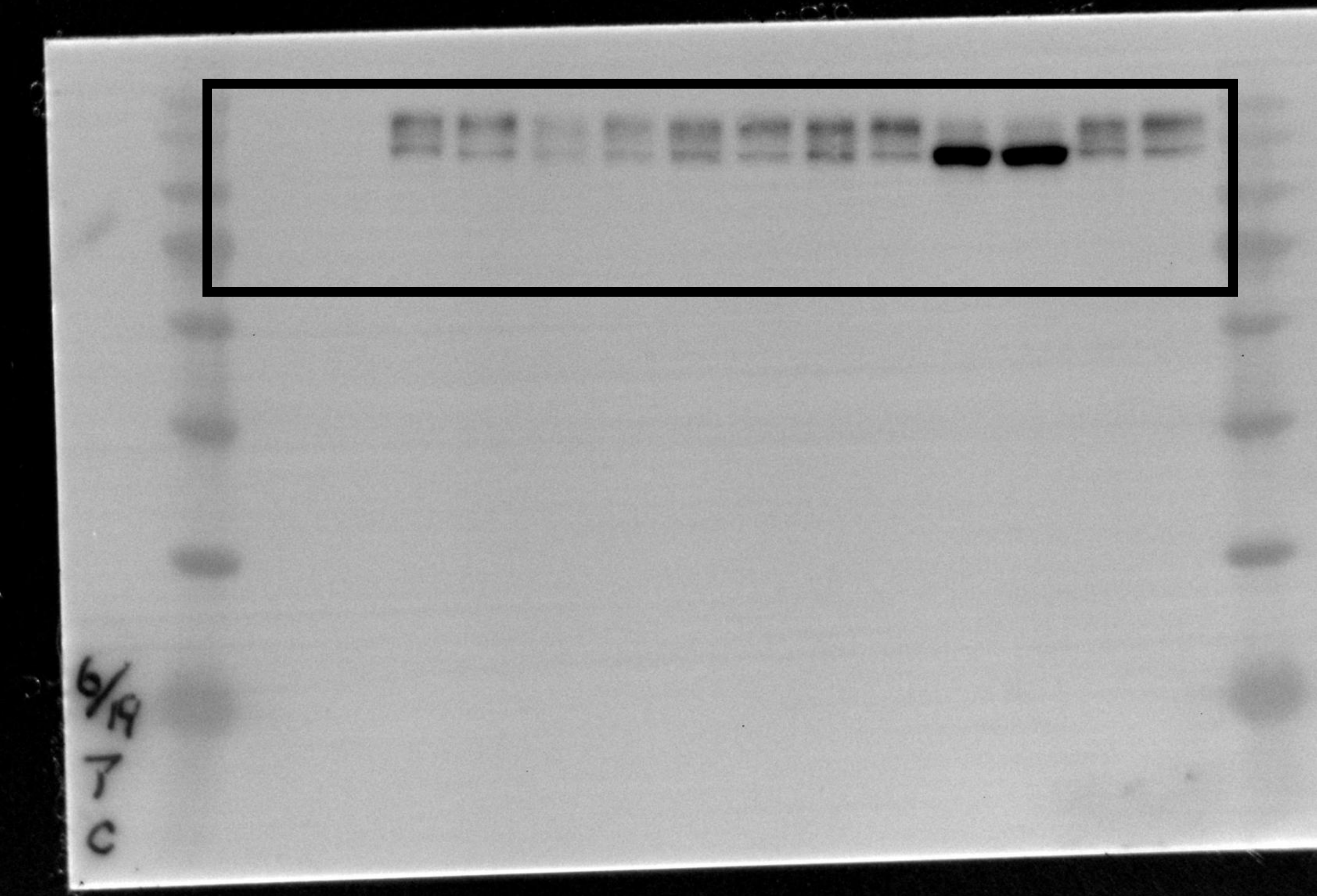

Full unedited blot for Figure S9D_cotransfection_3_β-actin

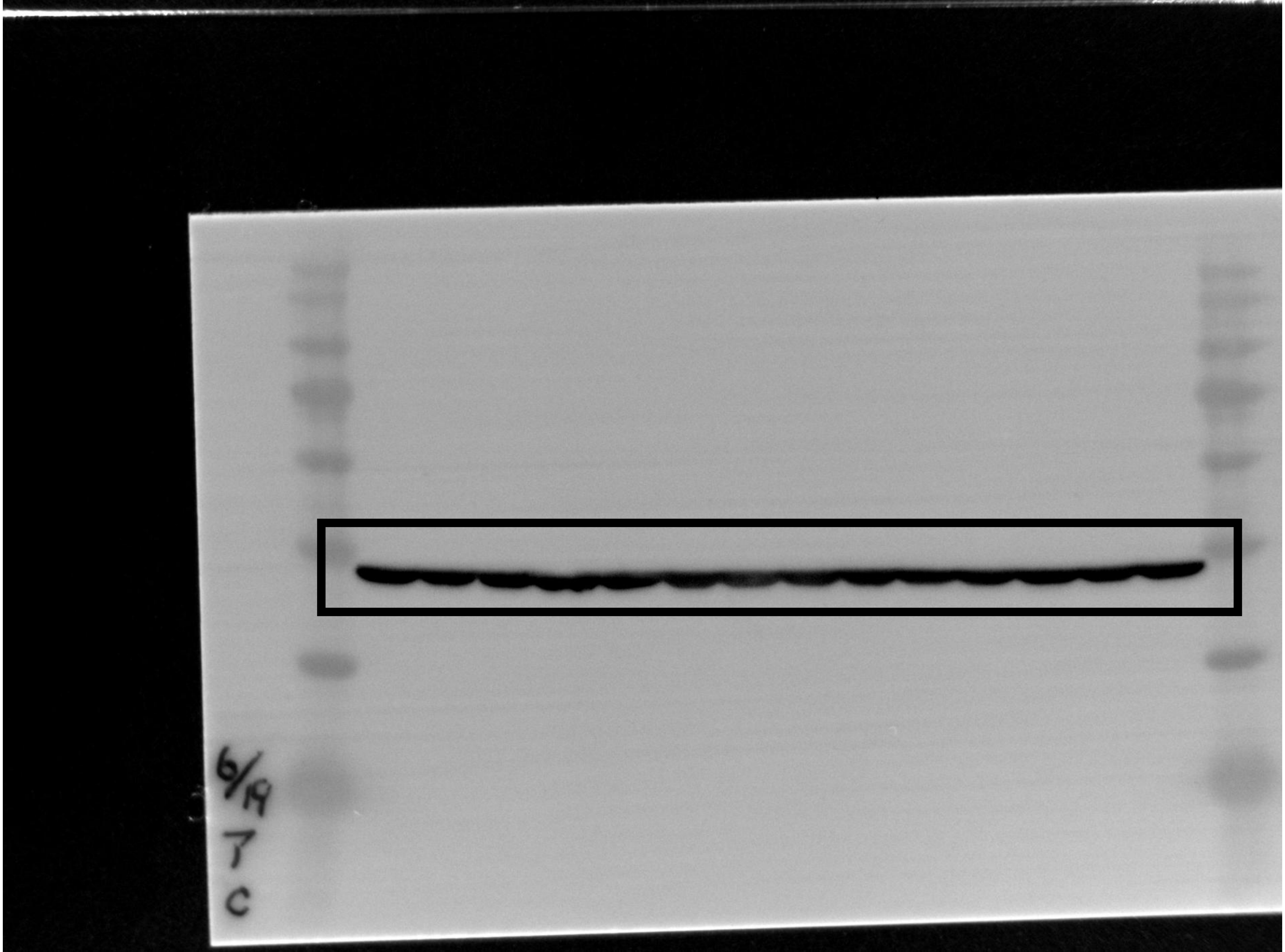

Full unedited blot for Figure S9D_cotransfection_4_pSTAT3

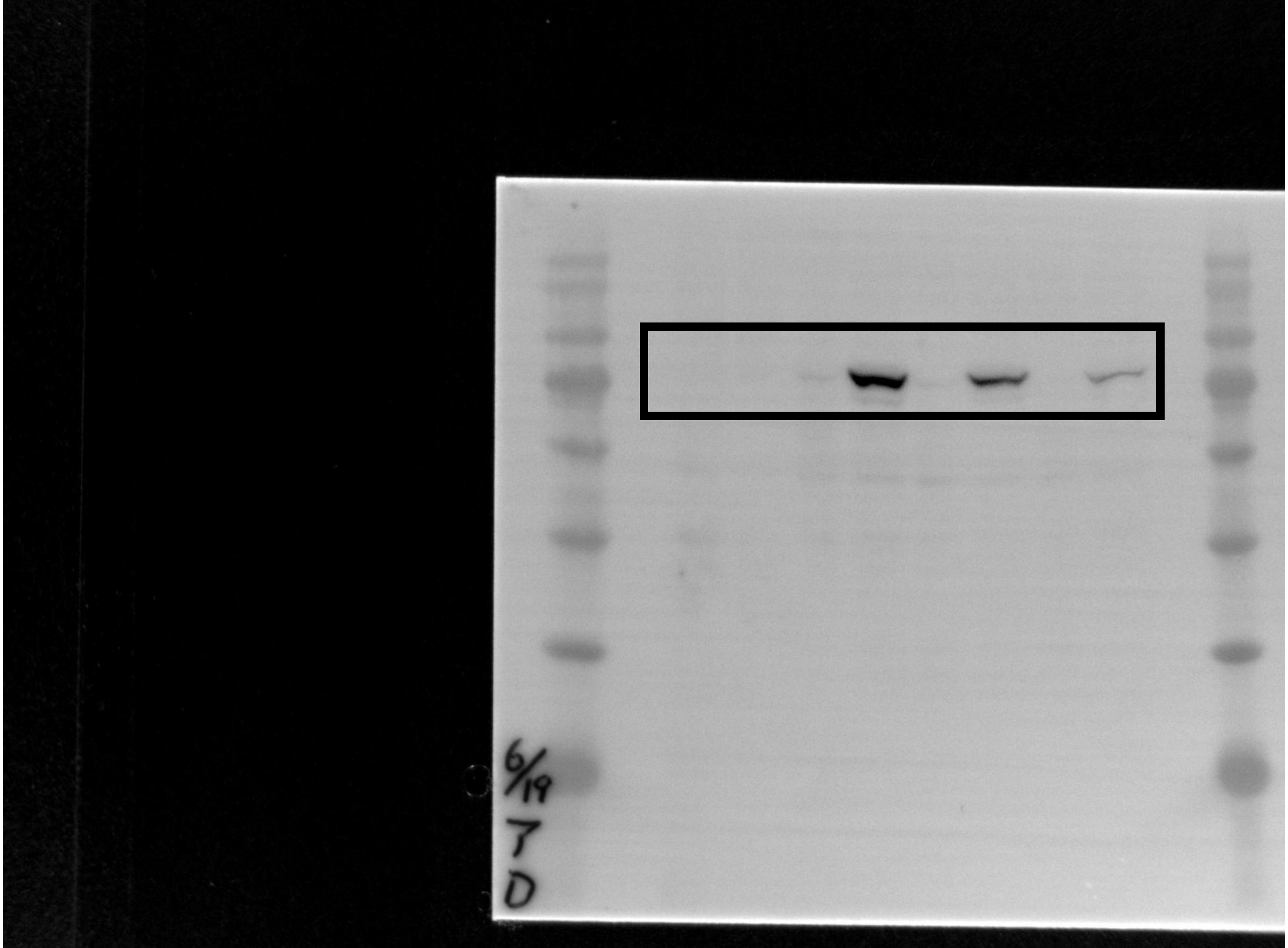

Full unedited blot for Figure S9D_cotransfection_4_STAT3

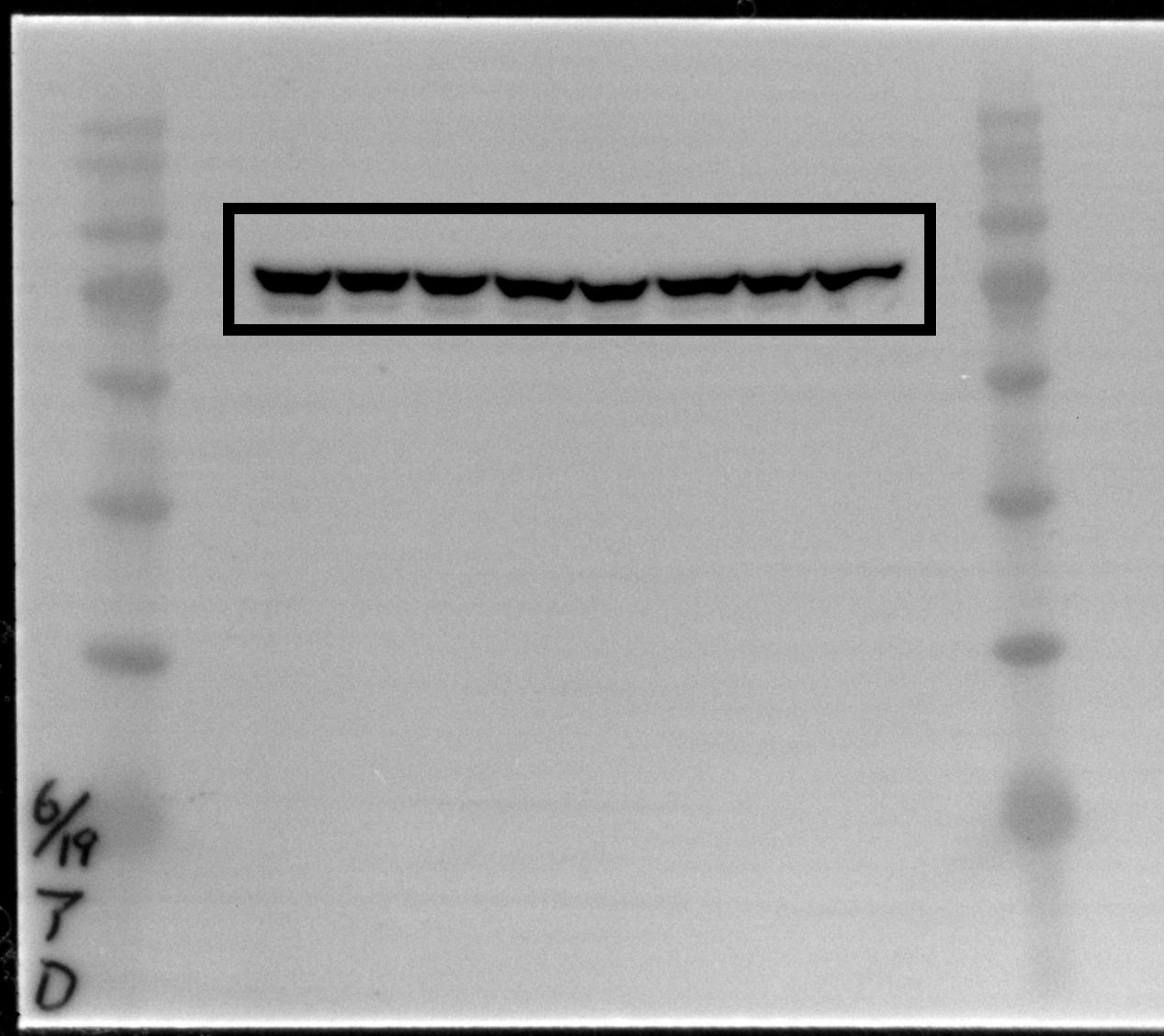

Full unedited blot for Figure S9D_cotransfection_4_GP130

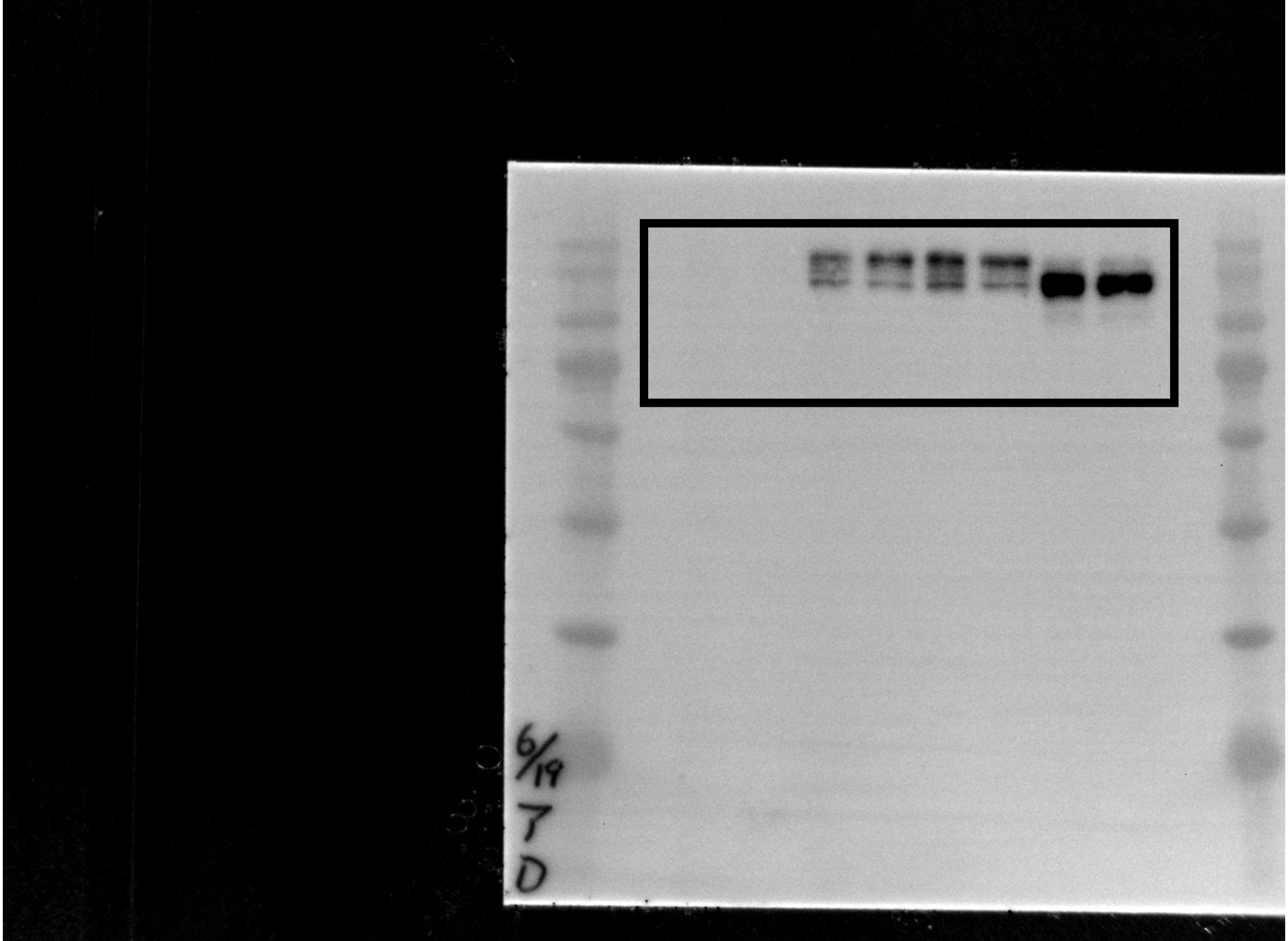

Full unedited blot for Figure S9D_cotransfection_4_β-actin

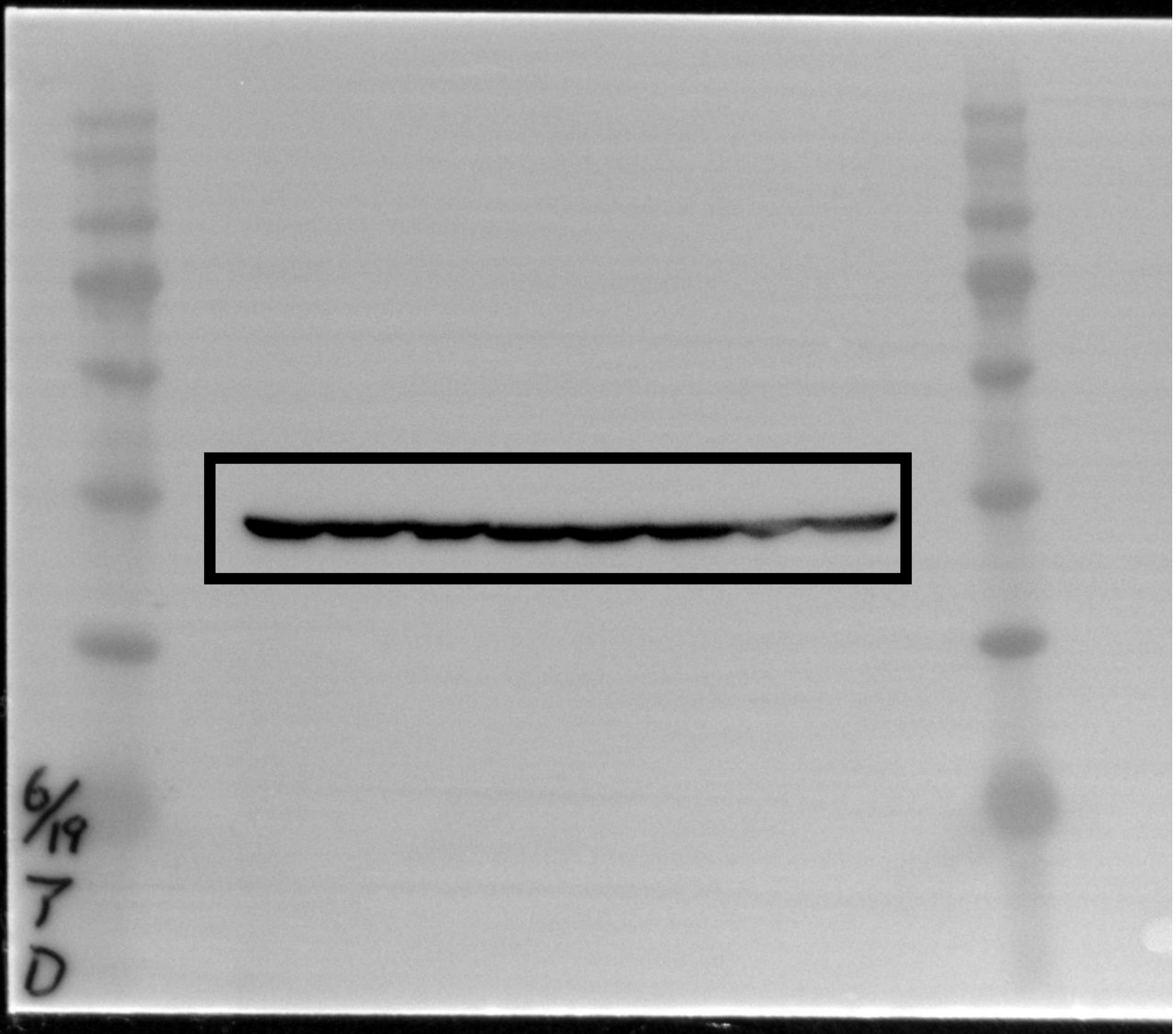

Full unedited blot for Figure S9D_single_1_GP130

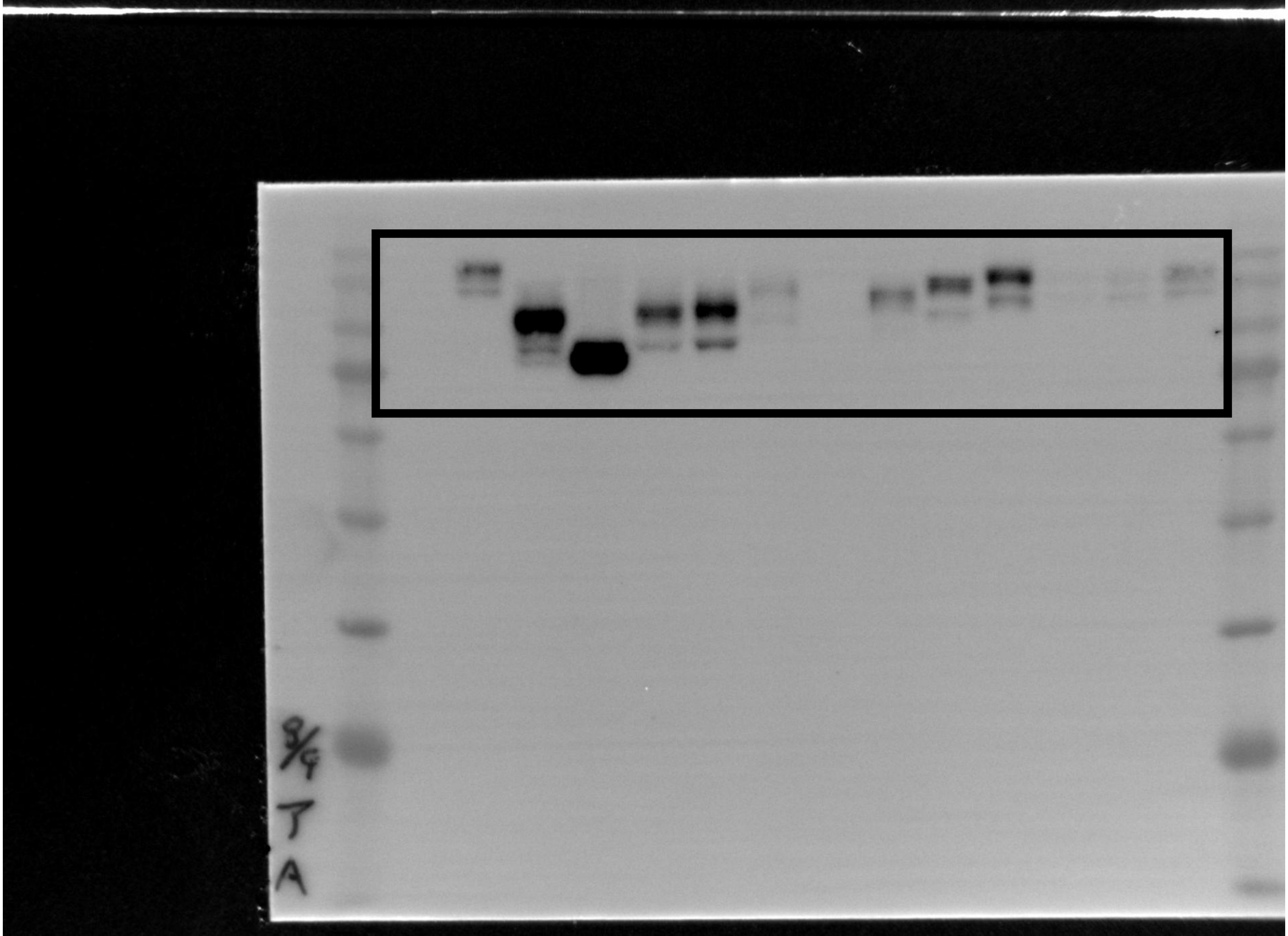

Full unedited blot for Figure S9D_single_1_β-actin

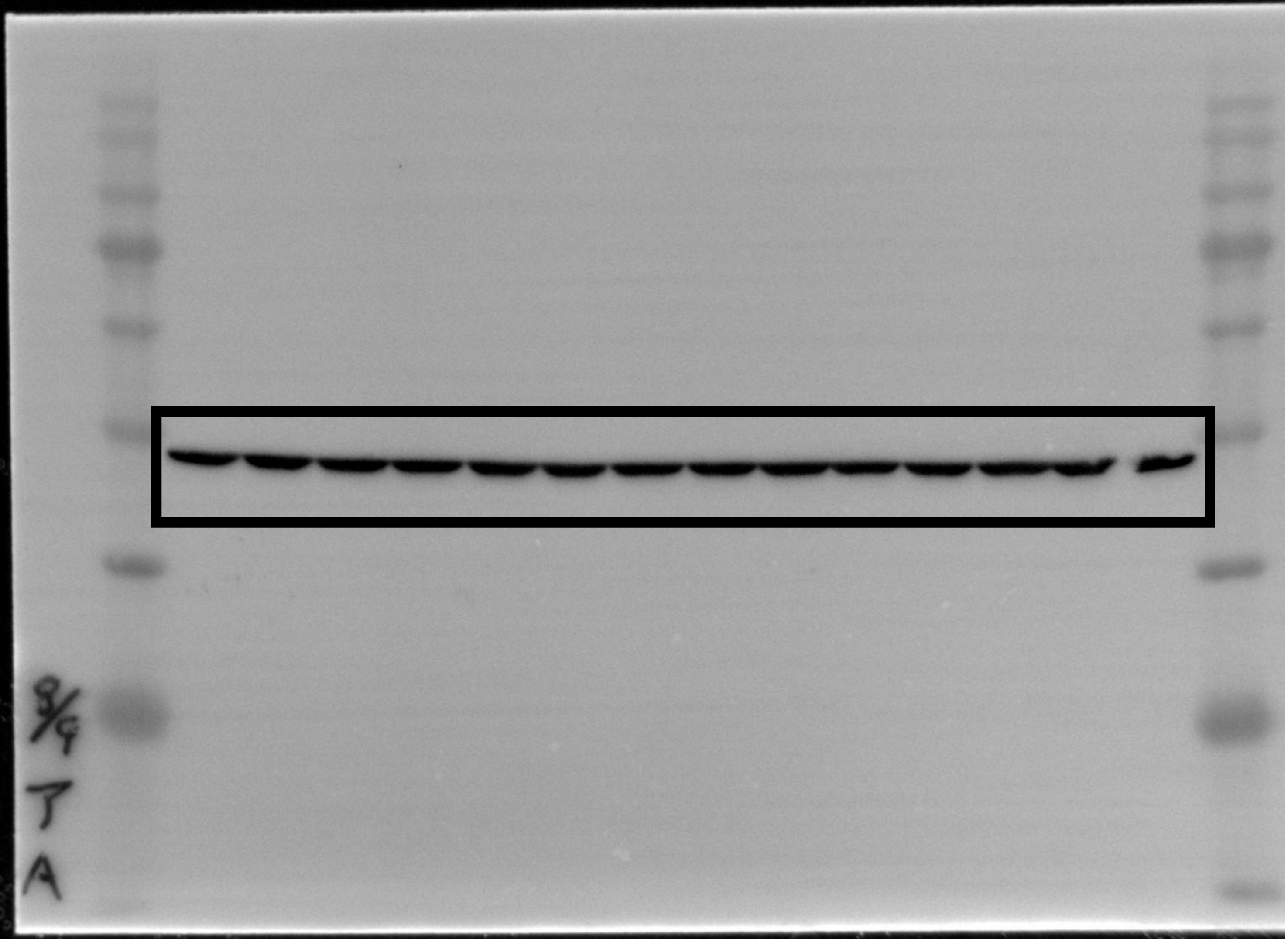

Full unedited blot for Figure S9D_single_2_GP130

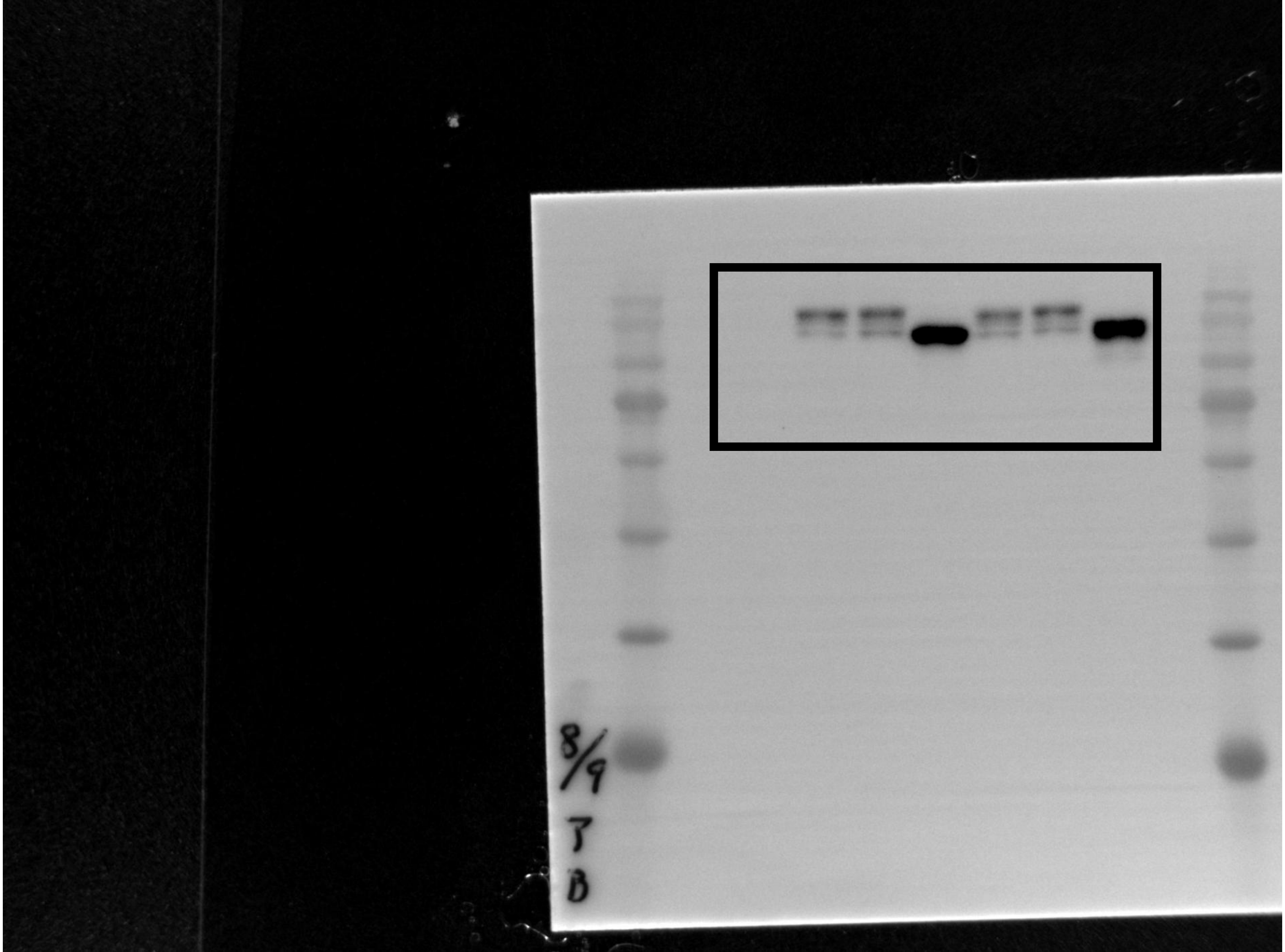

Full unedited blot for Figure S9D_single_2_β-actin

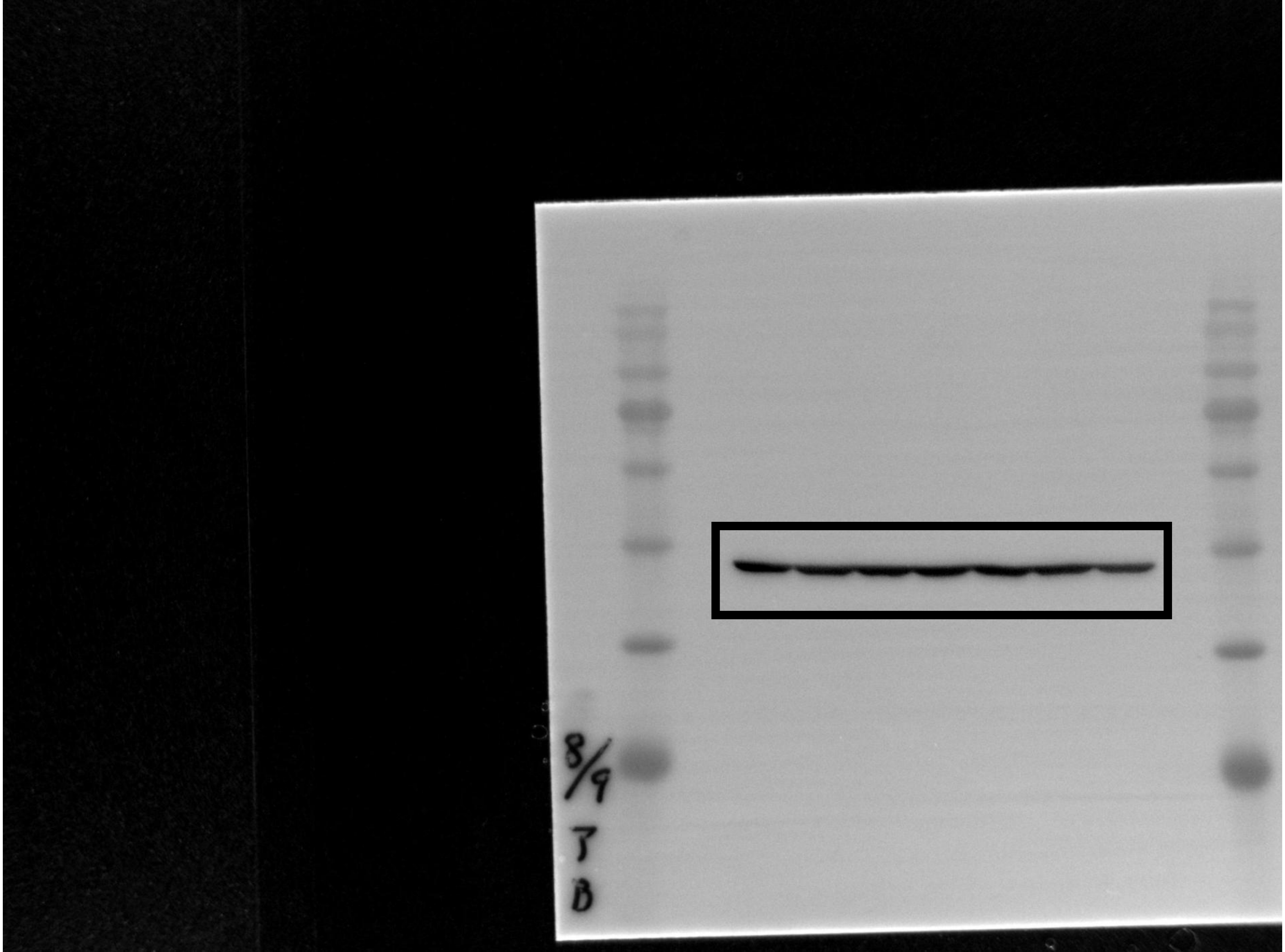

Supplement: Unedited blot and gel images [file jciinsight-10-190065-s163.pdf]
